# Tapered chiral nanoparticles as broad-spectrum thermally stable antivirals for SARS-CoV-2 variants

**DOI:** 10.1073/pnas.2310469121

**Published:** 2024-03-19

**Authors:** Rui Gao, Xinxin Xu, Prashant Kumar, Ye Liu, Hongyu Zhang, Xiao Guo, Maozhong Sun, Felippe Mariano Colombari, André F. de Moura, Changlong Hao, Jessica Ma, Emine Sumeyra Turali Emre, Minjeong Cha, Liguang Xu, Hua Kuang, Nicholas A. Kotov, Chuanlai Xu

**Affiliations:** ^a^International Joint Research Laboratory for Biointerface and Biodetection, School of Food Science and Technology, Jiangnan University, Wuxi, Jiangsu 214122, People’s Republic of China; ^b^State Key Laboratory of Food Science and Technology, Jiangnan University, Wuxi, Jiangsu 214122, People’s Republic of China; ^c^Department of Materials Science and Engineering, University of Michigan, Ann Arbor, MI 48109; ^d^Department of Chemical Engineering, University of Michigan, Ann Arbor, MI 48109; ^e^Biointerfaces Institute, University of Michigan, Ann Arbor, MI 48109; ^f^Institute of Medical Biology, Chinese Academy of Medical Sciences and Peking Union Medical College, Kunming, Yunnan 650000, People’s Republic of China; ^g^Brazilian Biorenewables National Laboratory, Brazilian Center for Research in Energy and Materials, Campinas, São Paulo 13083-100, Brazil; ^h^Department of Chemistry, Federal University of São Carlos, São Carlos, São Paulo 13565-905, Brazil; ^i^NSF Center for Complex Particles and Particle Systems (COMPASS), University of Michigan, Ann Arbor, MI 48109

**Keywords:** chiral nanoparticles, supraparticles, self-assembly, capsids, biomimetic

## Abstract

The incessant mutations of viruses, variable immune responses, and likely emergence of new viral threats necessitate multiple approaches to novel antiviral therapeutics. Furthermore, the new antiviral agents should have broad-spectrum activity and be environmentally stable. Here, we show that biocompatible tapered CuS nanoparticles (NPs) efficiently agglutinate coronaviruses with binding affinity dependent on the chirality of surface ligands and particle shape. *L-*penicillamine-stabilized NPs with left-handed curved apexes display half-maximal inhibitory concentrations (IC_50_) as low as 0.66 pM (1.4 ng/mL) and 0.57 pM (1.2 ng/mL) for pseudo-type SARS-CoV-2 viruses and wild-type Wuhan-1 SARS-CoV-2 viruses, respectively, which are about 1,100 times lower than those for antibodies (0.73 nM). Benefiting from strong NPs–protein interactions, the same particles are also effective against other strains of coronaviruses, such as HCoV-HKU1, HCoV-OC43, HCoV-NL63, and SARS-CoV-2 Omicron variants with IC_50_ values below 10 pM (21.8 ng/mL). Considering rapid response to outbreaks, exposure to elevated temperatures causes no change in the antiviral activity of NPs while antibodies are completely deactivated. Testing in mice indicates that the chirality-optimized NPs can serve as thermally stable analogs of antiviral biologics complementing the current spectrum of treatments.

## Significance

The Achilles heel of the best available antiviral mRNA vaccines and biomolecular drugs includes the loss of their potency due to viral mutations and thermal exposure. Chiral *L-* or *D-*penicillamine-coated CuS nanoparticles (NPs) provide an additional pathway to address these problems. The geometric complementary of their twisted conical shape to the spike protein of SARS-CoV-2 results in agglutination of the virus with antibody-like efficiency. Strong nanoparticle–protein interactions lead to broad-spectrum antiviral activity for several SARS-CoV-2 variants. An inhalable nano-formulation effectively protected mice from SARS-CoV-2 infection for 72 h. The combination of temperature robustness, curative capabilities, and broad activity makes possible utilization of chiral NPs as rapid-deployment antivirals for first responders and emergency biomedical stockpiles for potential pandemics.

Rapid development of Messenger RNA (mRNA) vaccines, antibody treatments, and small-molecule drugs collectively contributed to the marked reduction of infection rate and deaths from Severe Acute Respiratory Syndrome Coronavirus-2 (SARS-CoV-2). Along with scientific advances in drug delivery and design exploring nanoscale biomolecules (1–11), the COVID-19 pandemic also highlighted fundamental immunological and technological problems that remain to be solved for effective mitigation of future viral outbreaks. With respect to vaccines and biologics, these problems include rapid mutations (12), short shelf-life (13), and reversible virustatic binding (14). Thermal sensitivity and slow hydrolysis, even while in the frozen state, are the main contributors to shelf-life limitations and on-call availability. In addition to the deactivation of antibodies (15, 16), decomposition of high-molecular-weight biopolymers also causes strong and unpredictable immunological side effects observed, for example, for vaccines (17). It is fundamentally interesting and practically relevant to consider a previously underutilized set of chemical structures for the mitigation of viral infections. Altogether, the complexity and multifaceted nature of viral diseases and immune response to them underscores the necessity of developing antibody-like antivirals that would be inexpensive, versatile, thermally stable, and broadly accessible.

Water-soluble nanoparticles (NPs) have been shown to replicate many interactions of nanoscale biomolecules (18). Their exposure to biological fluids results in protein coronas (19, 20), a dynamic coating of the biomolecules around the NP surface. Complementarity of the surface chemistry, nanoscale shapes (21), and repulsive interactions can also lead to complexes between NPs and proteins (22), self-restricted supraparticles (SPs) (23), and supramolecular assemblies (24, 25). NPs can also replicate some biological functions, for instance, enzymatic catalysis (26, 27) and cell signaling (28). The biomimetic functionalities of NPs also manifest in their targeted binding to capsids of SARS-CoV-2 (29–31) and other viruses, which diminishes their ability to bind to and infect host cells (13, 32). The strong NP–virus interactions can deform proteins in viral capsids (33), and when combined with some inherent small variability in their structures, the result is inhibition and/or inactivation of viruses (34). This ability can be enhanced by multiparameter design of the NPs to fit the geometry and chemistry (35) of the capsid proteins (36), potentially leading to broad-spectrum antiviral activity (31, 33).

One of the essential components of the COVID virus is the spike protein (*S*) that received extensive attention because it is responsible for binding with the angiotensin-converting enzyme-2 (ACE2) receptor in the membranes of host cells (37). As a part of virus, the homotrimeric spike complex has a molecular weight of 195 kDa, a length of 16 nm, and a width of 12 nm (38). Although some amino acids in SARS-CoV-2 mutants may vary, the overall geometry of the spike does not. The spike protein is composed of S1 (130 kDa, 6 nm) and S2 (65 kDa, 4 nm) subunits as well as the receptor-binding domain (RBD, 35 kDa, 3 nm) (39). Macromolecular RBD segment is a part of S1 and is located on the top of the spike and interfaces with ACE2. These segments of *S1* are primarily composed of β-sheets and random coils that form a groove to create a geometrical lock-and-key match for the protruding *N*-terminal α-helices in ACE2. The S2 subunit in *S* is mainly composed of stiff α-helices (40) forming the stem of the spike.

Chirality is an essential geometrical and chemical factor for the formation of protein-protein and protein-nanoparticle complexes. Besides the chirality of amino acids, the strong circular dichroism (CD) (41) of the homotrimeric spike protein and high values of Osipov–Pickup–Dunmur chirality measures in the RBD segment interfacing with ACE2 (*SI Appendix*, Fig. S1) indicate that both the molecular and nanoscale chirality of NPs can be significant in targeting SARS-CoV-2 viruses. Based on prior studies showing the effects of multiscale chirality of NPs on their affinity to biomolecules (42–46) in protein coronas (19) and cell membranes (28), we chose to examine tapered chiral NPs for agglutination of several strains of coronaviruses.

## Results and Discussion

### Tapered CuS NPs with Multiscale Chirality.

**Fig. 1.**
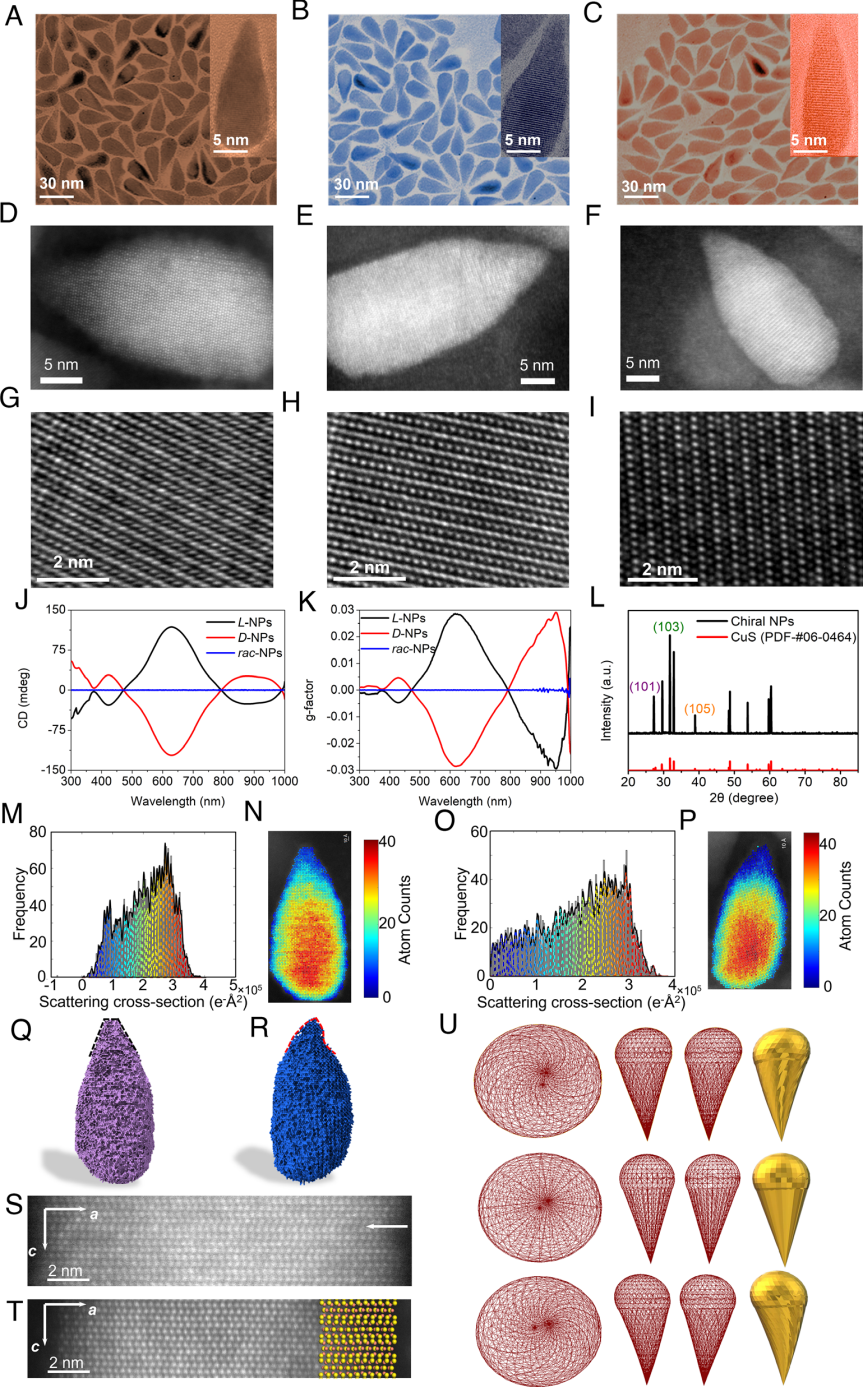
Fabrication and characterization of chiral NPs. TEM images of *L*-NPs (*A*), *D*-NPs (*B*), and *rac*-NPs (*C*). ADF-STEM of *L*-NP (*D*), *D*-NP (*E*), and *rac*-NP (*F*). HR-TEM image of *L*-NP (*G*), *D*-NP (*H*), and *rac*-NP (*I*). The CD spectra (*J*) and *g*-factor spectra (*K*) of chiral NPs. (*L*) XRD result of *L*-NPs. Scattering cross-section histogram (*M*) and estimated atom count heat map (*N*) for no chiral ligands modified NP. Scattering cross-section histogram (*O*) and estimated atom count heat map (*P*) for L-NP. 3D reconstruction of no chiral ligands modified NPs (*Q*) and *L*-NP (*R*). (*S*) ADF-STEM image of a section of no chiral ligands modified NP, highlighting the presence of planar dislocations by the white arrow. (*T*) ADF-STEM image of the section of *L*-NP, showing the stress-annealed NP structure. Atomic structure with S (yellow) and Cu (brown) is overlaid on the image. (*U*) 3D reconstruction of NPs. Three different projections of the mesh along with the solid surface representation of the reconstructed *L*-NPs (*Above* row), *rac*-NPs (*Middle* row), and *D*-NPs (*Down* row). The reconstruction is done by merging a sphere of diameter 13.5 (nm) and a cone with a base of radius 13.5 (nm) and height 26 (nm).

We employed tapered CuS NPs with a length of 30 ± 3 nm and a base diameter of 10 ± 1 nm (*SI Appendix*, Figs. S2 and S3) because the apexes of these NPs have diameters of 2.5 ± 0.2 nm, which makes them comparable in dimension and geometry to the typical grooves, pockets, and trenches in protein complexes (36). NPs with apexes also inhibit enzymes (35), which contributed to the selection of tapered NPs for this work. The standard synthesis (47) followed by hydrophobic-to-hydrophilic ligand exchange resulted in chirality in multiple scales that includes angstrom scale chirality of *L/D* enantiomeric excess on CuS surface and the nanoscale curved geometry of the NP apex (Fig. 1 *A*–*I* and *SI Appendix*, Figs. S2 and S3). Chiral tapered CuS NPs carrying penicillamine (Pen) were denoted as *D-*NPs, *L-*NPs, and *rac-*NPs when *D-, L-,* and *rac-*Pen were used as surface ligands.

The total number of Pen ligands on the surface of the *L*-NPs was approximately 320 as determined by liquid chromatography–mass spectrometry (*SI Appendix*, Fig. S3). The Pen layer imparts the overall negative charge to the particles. The *L*-, *D*-, and *rac-*NPs displayed zeta potentials of −7.9 ± 0.4 mV, −8.1 ± 0.5 mV, and −17.9 ± 0.12 mV, respectively, at physiological pH 7.4 (*SI Appendix*, Fig. S3 and Table S1). The *S* and RBD display zeta potentials of −59.94 ±1.45 mV and 2.60 ± 0.09 mV, respectively, indicating the possibility of an electrostatically controlled complex between CuS NPs and RBD under biological conditions. The CD spectra of the *D*-NPs and *L*-NPs showed broad-range optical activity from 300 nm to 1,000 nm with intense peaks at 423 nm (28 ± 2 mdeg), 630 nm (118 ± 4 mdeg), and a wide peak at 877 nm (Fig. 1*J*). The optical asymmetry parameter, that is, the *g*-factor, was 0.03 ± 0.002 for both L- and D-NPs at both 950 nm and 620 nm (Fig. 1*K*).

NPs carrying other ligands, such as citric acid, *L-*malic acid, *L-*tartaric acid, and *L-*glutathione (GSH), displayed markedly different and typically smaller optical activity than Pen-coated NPs (*SI Appendix*, Fig. S4), which prompted us to better understand how the molecular scale of Pen’s chirality gives rise to the optical bands in the red and near-infrared parts of the spectrum associated with nanoscale chirality of CuS. The crystal lattice fringes observed in Transmission Electron Microscope (TEM) images of Pen-coated NPs displayed a characteristic spacing of 0.281 nm. This corresponds to a (103) plane of the hexagonal crystal lattice (*SI Appendix*, Figs. S2 and S5), which was consistent with X-ray diffraction (XRD, Fig. 1*L*) and X-ray photoelectron spectroscopy data (XPS, *SI Appendix*, Fig. S6). We reconstructed the overall geometry and unit cell structure for different Pen-functionalized NPs from the TEM images using a Gaussian mixture model and integration classification likelihood criterion (48). (Fig. 1 *M*–*P*, *SI Appendix*, Fig. S7, and Movie S1). We found that the approximately 5-nm apex in a *L-*Pen NP is offset from the center line by ~1 nm and the tapered section is twisted in the counterclockwise and clockwise directions for *L*-NPs and *D*-NPs, respectively, while remaining untwisted in *rac*-NPs and ligand-free NPs (Fig. 1 *Q*–*T* and Movie S1). The dislocation maps (*SI Appendix*, Fig. S8) show that the twist emerges from the relaxation of the lattice stress accumulated during the nucleation and growth in the hydrophobic medium of 1-dodecanethiol and trioctylphosphine oxide. The twist deformation of the apex occurs via sliding of atomic planes; it is caused by the exchange of the original synthetic ligands to Pen (Fig. 1*U* and *SI Appendix*, Fig. S8). Since the CD spectra are quite different (*SI Appendix*, Fig. S4) and apex twists were not observed for other surface ligands (*SI Appendix*, Fig. S4), the twist dislocations are directly related to the chemistry of surface ligand binding. ^1^H NMR, Raman, and FTIR data (*SI Appendix*, Fig. S9) indicate that both amino acid residues in Pen are bonded with the NP surface via a strong S-Cu covalent bond, S–H–O hydrogen bonds, and ionic bonds between COO^−^ and Cu^2+^ on the NP surface.

### Interactions of Chiral NPs and Viral Proteins.

**Fig. 2.**
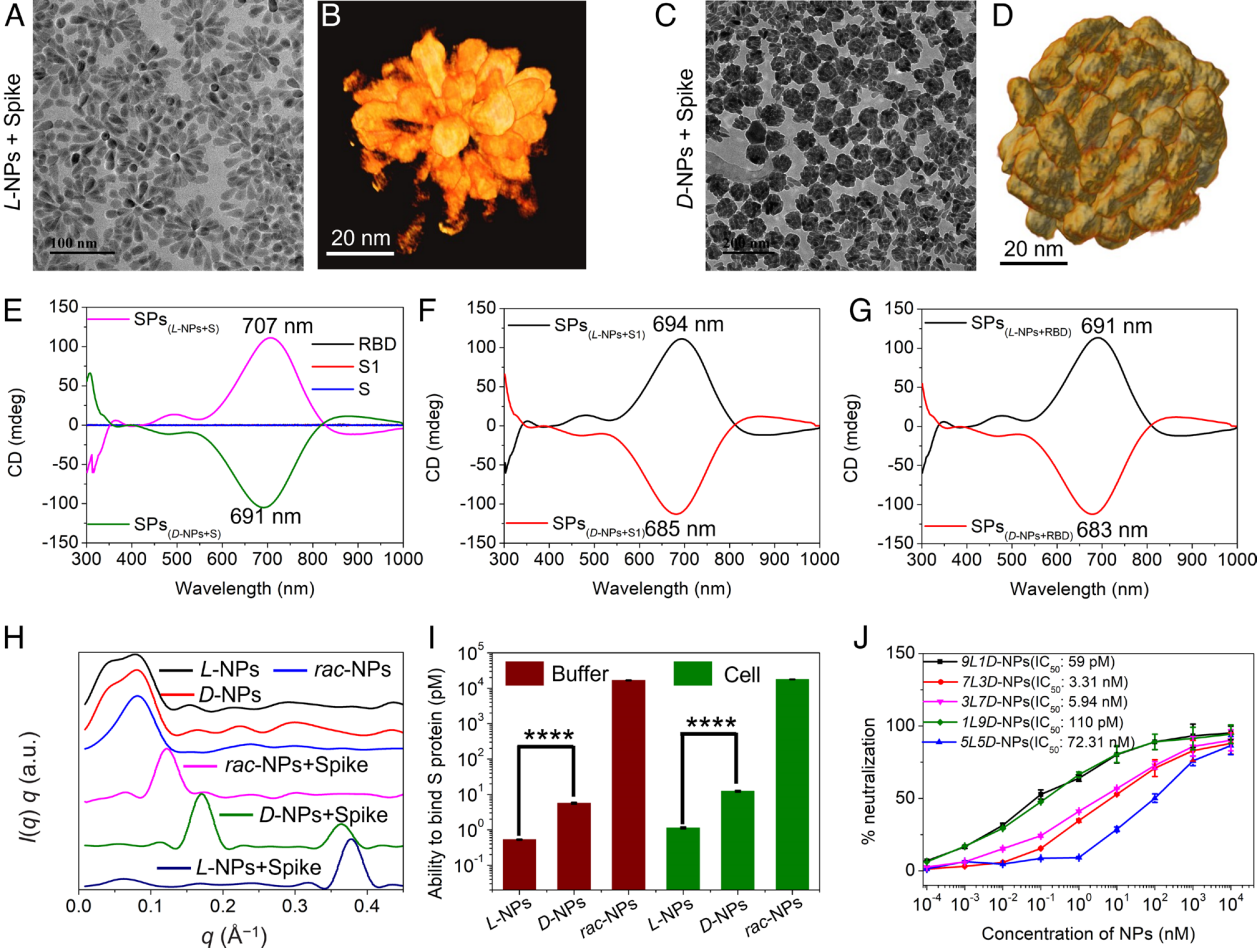
Characterization of SPs produced by chiral NPs with different handedness incubated with spike protein. TEM image (*A*) and TEM tomography image (*B*) of *L*-NPs incubated with *S* for 2 h to form SPs. TEM image (*C*) and TEM tomography image (*D*) of *D*-NPs incubated with *S* for 2 h to form SPs. (*E*) The CD spectra of three proteins and SPs _(S +_ *_L_*_-NPs)_ as well as SPs _(S +_ *_D_*_-NPs)_. (*F*) The CD spectra of SPs _(S1 +_ *_L_*_-NPs)_ and SPs _(S1 +_ *_D_*_-NPs)_. (*G*) The CD spectra of SPs _(RBD +_ *_L_*_-NPs)_ and SPs _(RBD +_ *_D_*_-NPs)_. (*H*) SAXS of chiral NPs and chiral NPs interacted with *S*. (*I*) The ability to bind *S* protein of chiral NPs in buffer and in cells. (*J*) The neutralization effect for NPs with different *L/D* ratios of Pen ligands. Data are presented as mean ± SD (n=3) with the error bar representing the standard deviation. *p<0.05, ** p<0.01, ***p<0.001, analyzed by Student’s t-test.

To assess the interactions of *L-, D-,* and *rac-*CuS NPs with viral proteins, we used the entire *S* protein, subunit S1, and recombinant RBD. Because strong attraction overcomes the electrostatic repulsion, we found that *S* incubated with *L*-NPs for 2 h formed SPs (Fig. 2*A*), where *L*-NPs acquired a predominantly radial orientation with the NP tip pointed inward (Fig. 2 *A* and *B*, *SI Appendix*, Fig. S10, and Movie S2). We note that SPs are the result of competitive restrictions on particle shape and size originating from collective electrostatic, van der Waals, hydrophobic and supramolecular interactions. Because of the electrostatic repulsion, SPs display narrow size distribution. Because of the collective effects, the organizational patterns of SPs combining order and disorder is strongly dependent on their lock-and-key complementarity that can be tuned by chirality of the surface ligands and overall geometry of NPs. When *S* was exposed to *D*-NPs for 2 h, SPs also formed; however, the *D*-NPs acquired a predominantly lateral orientation (Fig. 2 *C* and *D*, *SI Appendix*, Fig. S10, and Movie S3). Cryoelectron tomography visualized the difference in the organization of SPs with NPs of different chirality. When *L*-Pen was used as the surface ligands, a tightly interconnected network of the tapered chiral NPs and *S* was observed (Fig. 2*B*, *SI Appendix*, Fig. S10, and Movie S2). The ability of *L-*NPs and *D-*NPs to form SPs is analogous to agglutination of proteins by biomacromolecules via multivalent target–ligand interactions (13). The average number of *S* proteins that bind to a single *L-*NP is 4.3 ± 0.3.

Similar to the *S*, its subunit S1 and RBD also formed SPs with both *L-* and *D-*NPs (*SI Appendix*, Fig. S11). However, no SPs appeared when *rac*-NPs were incubated with RBD, S1, or *S* (*SI Appendix*, Fig. S12) indicating the critical importance of chirality of NPs in formation of these multicomponent NP-protein complexes. As the ratio of *L*-Pen to *D*-Pen per NP increased, the tendency to form SPs also increased (*SI Appendix*, Fig. S12). No agglomeration leading to SPs or other assemblies was observed without proteins or with nucleocapsid protein of SARS-CoV-2 (70 kDa, 5 nm), bovine serum albumin, and human serum albumin, (*SI Appendix*, Fig. S13), which indicated binding specificity. Note that the presence of other proteins in the media and formation of protein coronas does not prevent the formation of strong NP–protein complexes.

The CD spectra of the SPs formed as a result of interactions between NPs and *S* showed a 70-nm red-shift with respect to the parent NPs (Fig. 2 *E*–*G*). *L*-NPs exhibited a larger red-shift than *D*-NPs in the presence of the three proteins with the maximum red-shift of 77 nm observed complexes of *L*-NPs with *S* (Fig. 2*E*). Small Angle X-ray Scattering (SAXS) data showed that *L*-NPs, *D*-NPs, and *rac*-NPs exhibited different *q* values after interacting with spike proteins (Fig. 2*H*). Raman scattering spectra were enhanced by *L*-NPs to a greater extent than by *D*-NPs, while *rac*-NPs caused almost no enhancement, altogether correlating with the binding affinities for the same NP–protein pairs (*SI Appendix*, Fig. S14). XPS data for SPs produced by *L-* and *D-*NPs confirmed that *L*-NPs interact with *S* stronger than *D*-NPs as evidenced by the binding energy of N and O atoms shifting to lower values, which is due to N-Cu and O-Cu coordination bonds in the SPs (*SI Appendix*, Fig. S14). The SP formation monitored by dynamic light scattering, zeta potential, and TEM showed nearly identical kinetics and confirmed the rapid agglutination of *S* by *L-*NPs (*SI Appendix*, Fig. S15).

The binding of NPs to viral proteins was also evaluated using the ELISA assay and flow cytometry after NP binding to HEK293 cells expressing viral proteins in their membranes (*SI Appendix*, Figs. S16–S20). We found that the binding affinity of *L-*NPs to viral proteins in vitro and in cells is nearly the same (Fig. 2*I* and *SI Appendix*, Figs. S17 and S18 and Table S2). The binding affinity between *D-*NPs and the three proteins followed the same trend, but the values were considerably smaller than for *L-*NPs (Fig. 2*I* and *SI Appendix*, Figs. S17 and S18 and Table S2). The *rac*-NPs display even lower affinity to the three viral proteins (Fig. 2*I* and *SI Appendix*, Figs. S17 and S18 and Table S2). Compared with *L*-NPs, *D*-NPs and *rac-*NPs showed approximately 10-fold and >10^3^-fold reduced affinity for the three viral proteins (*SI Appendix*, Table S2), respectively. Confocal images of *L-* and *D-*NPs binding three viral proteins in cells also confirmed the higher affinity of *L*-NPs to viral proteins compared to *D*-NPs (*SI Appendix*, Fig. S19). As the ratio of *L*-Pen or *D*-Pen increased in the coating of the NPs, the affinity between chiral NPs and viral proteins also increased (Fig. 2*J*). Importantly, the viability of the human bronchial epithelioid cells (BEAS-2B) remained high, even at high NP concentrations (*SI Appendix*, Fig. S20).

Tapered NPs surface decorated with several other chiral and achiral surface ligands (*SI Appendix*, Fig. S4), such as glutathione (GSH), citric acid (Cit), tartaric acid (TA), and malic acid (MA), were also tested with *S*. The IC_50_ values, that is, the NP concentration needed to bind 50% of *S* proteins (35), were 11.2 nM for GSH (24.36 μg/mL), 17.5 nM for Cit (38.07 μg/mL), 27.1 nM for TA (58.95 μg/mL), and 8.8 nM for MA (19.14 μg/mL). All these IC_50_ values were about four orders of magnitude higher than those for *L*-NPs (*SI Appendix*, Fig. S21). No SPs were observed for these NPs with *S*, even after 6 h of incubation (*SI Appendix*, Fig. S21). We also tested *L*-Pen functionalized NPs with different geometrical shapes and related chemistries, such as 8-nm and 20-nm CuS nanospheres, 40-nm-long CuS nanorods, and 3-nm Cu_1.94_S nanospheres (*SI Appendix*, Fig. S22). Their IC_50_ values for *S* were 15.5 nM, 4.5 nM, 12.62 nM, and 13.81 nM, respectively, which is again multiple orders of magnitude greater than for *L*-NPs (*SI Appendix*, Fig. S23). Other nanomaterials with different morphologies and functionalized with *L*-Pen, such as ZnO and Au NPs, showed limited affinity to RBD with IC_50_ values of 9.2 mM and 10.8 μM, respectively. Again, no SPs were formed, indicating that the weak attractive interactions between them are attributed to the lack of geometrical match (*SI Appendix*, Fig. S24). Finally, *L*-Pen modified SiO_2_ NPs showed no affinity to RBD, even at very high concentrations (*SI Appendix*, Fig. S24 and Table S1).

**Fig. 3.**
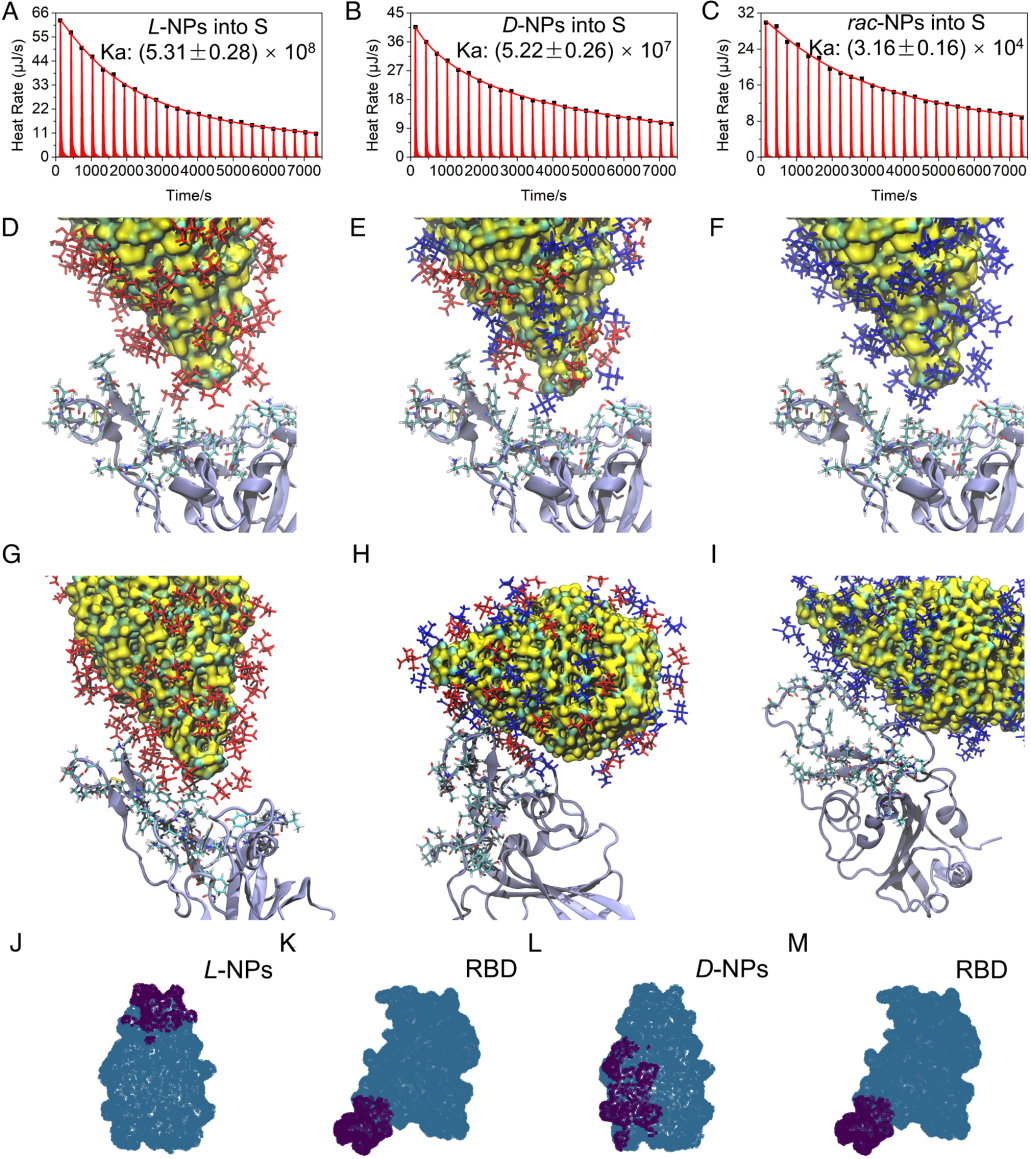
The interactions between chiral NPs and spike protein and their different computational models. The ITC trajectories for interactions of *S* with *L*-NPs (*A*), *D*-NPs (*B*), and *rac*-NPs (*C*). Initial structures of the RBD with *L*-NP (*D*), RBD with *rac*-NP (*E*), and RBD with *D*-NP (*F*). The protein is depicted as an iceblue chain, and the ligands are depicted either in red or blue for *L*-Pen and *D*-Pen, respectively. The NPs were placed with the tip in contact with the RBD portion that originally interacts with the ACE2 in the original PDB file (PDB entry 6m0j, residues 449 to 458 and 476 to 505). Final structures of the RBD with *L*-NP (*G*), RBD with *rac*-NP (*H*), and RBD with *D*-NP (*I*) after 1,000 ns of MD simulations. (*J*-*M*) Predictions for binding sites for NPs of different chirality using unified structural descriptors for NP and proteins with chemical, GT and geometric parameters, including chirality measures at multiple scales. ML predicts the binding site between *L*-NPs (*J*) and RBD (*K*) as well as *D*-NPs (*L*) and RBD (*M*).

The chirality-dependent difference in NP affinity is directly related to the thermodynamics of their interactions. Isothermal titration calorimetry (ITC) revealed that the affinity constants, *K_a_*, for the binding of *L*-NPs to *S*, the S1 subunit, and RBD were (5.31 ± 0.28) × 10^8^, (8.66 ± 0.41) × 10^7^, and (2.35 ± 0.12) × 10^8^, respectively. *K_a_* values for the *D*-NPs binding to the same three viral components were (5.22 ± 0.26) × 10^7^, (2.02 ± 0.11) × 10^7^, and (2.33 ± 0.12) × 10^7^, respectively (Fig. 3 *A*–*C* and *SI Appendix*, Fig. S25). Note that *K_a_* values for both *L*-NPs and *D*-NPs with *S* were more than two times higher than those for S1 and RBD. In parallel with the IC_50_ values, *K_a_* values for *rac*-NPs were much lower than those for *L-* or *D-*NPs: *S* − *K_a_* = (3.16 ± 0.16) × 10^4^; S1 − *K_a_* = (9.16 ± 0.48) × 10^5^; and RBD − *K_a_* = (6.52 ± 0.29) × 10^4^ (*SI Appendix*, Table S2).

### Computational Models for Chiral NPs–Protein Interactions.

Comparing quantitative and qualitative characteristics for binding between NPs and the viral proteins presented above, one can see that geometrical and chemical complementarity between NP apex and RBD leads to high affinity between NPs and spike proteins of the SARS-CoV-2 virus. This point can be substantiated by atomistic molecular dynamics (MD) simulations (Fig. 3 *D*–*I* and *SI Appendix*, Fig. S26). We observed that *L*-NPs consistently interacted with RBD in the apex area (Fig. 3 *D* and *G*), while *D*-NPs and the *rac*-NPs predominantly interacted with RBD laterally on the sides. *D*-NPs and the *rac*-NPs also changed their relative orientation with respect to RBD during the simulations to maximize side-spike contact (Fig. 3 *E*, *F*, *H*, and *I*). Such preferential orientation is consistent with the experimentally observed patterns of aggregation for the SPs with radial and lateral placement of the NPs (Fig. 2 *B* and *D*). Based on the analysis of trajectories obtained between 150 and 1,000 ns of simulation time (*SI Appendix*, Fig. S27), one *L*-NP can bind 1.9 RBDs, one *D*-NP can bind 0.17 RBDs, and one *rac*-NP can bind 0.0006 RBDs. The interaction energies of NPs with RBD were calculated as –1223.6 kJ/mol, –125.3 kJ/mol, and 0.005 kJ/mol which accurately reflects the IC_50_ and *K_a_* values in *SI Appendix*, Table S2.

We also compared MD predictions with those based on machine learning (ML) using previously developed algorithms using unified structural descriptors for NPs and proteins (49). The structural descriptors for quantitative biomimetics of NPs and proteins incorporate geometric, chemical, and Graph Theoretical (GT) parameters of NPs and proteins, including chirality at multiple scales. Chirality measures cumulatively capture the geometric complementarity while the GT parameters capture the reconfigurability of the protein. We found that *L*-NPs and RBD interact primality via the twisted apex of the NPs and the groove segment (Fig. 3 *J* and *L*). The interaction sites between *D*-NPs and RBD are very different and located on the sides of the NPs (Fig. 3 *K* and *M*), which confirms the mechanism of antiviral activity. Also importantly, these data indicate that chirality at both the molecular scale (Pen surface layer) and the nanoscale (twisted apex) affects the NP–protein interactions.

### Inhibition of Binding of Viral Proteins to ACE2 by Chiral NPs In Vitro.

The strong chirality-dependent interactions between *S* and NPs prompted us to incubate *L-, D-,* and *rac-*NPs with pseudo-type SARS-CoV-2 (in 10 mM PBS buffer) for different time points. TEM images showed different stages of agglutination that, in the case of *L*-NPs, led to complete coverage of virus by NPs after 2 h and complete destruction of the viral envelope as indicated by negative staining TEM imaging (*SI Appendix*, Fig. S28). *D-*NPs and *rac*-NPs displayed slightly lower and negligible association with this virus, respectively (*SI Appendix*, Fig. S28).

The binding between *L-*NPs and protein components of SARS-CoV-2 is strong (*SI Appendix*, Table S2). The formation of the NP–protein indeed inhibits the binding between viral proteins and ACE2 on the surface of mammalian cells. We first investigated whether chiral NPs affect the binding between viral proteins and ACE2 receptors in vitro by using ELISA. *L*-NPs effectively inhibit the binding of viral proteins to ACE2 with IC_50_ values between 0.52 pM (1.13 ng/mL) and 2.06 pM (4.48 ng/mL) for all three proteins. (*SI Appendix*, Figs. S29–S31 and Table S3). The inhibitory effect of *L*-NPs was more than 10 times higher than that of *D*-NPs. For example, *D*-NPs inhibit the binding of *S* protein to ACE2 with an IC_50_ = 5.72 pM (12.44 ng/mL), while *L*-NPs do the same with an IC_50_ = 0.52 pM (1.13 ng/mL). *Rac*-NPs also inhibit binding between viral proteins and ACE2, but only at much higher concentrations, with IC_50_ values of 32.29 nM (70.24 μg/mL) (*SI Appendix*, Figs. S29–S31 and Table S3).

The role of chirality in binding can be further substantiated by the CD spectra that characterize the change in configuration when the three viral proteins bind to ACE2. The CD peaks of ACE2 located at 209 nm and 223 nm, which are associated with its α-helices, decreased in intensity and red-shifted after binding with RBD. Importantly, the CD spectrum of ACE2 recovered to its original state when *L-*NPs were added (*SI Appendix*, Fig. S32), which showed higher affinities between NPs and viral proteins compared to ACE2 and viral proteins. As expected, CD spectra display large shifts in peaks for RBD, S1, and *S* after they interacted with *L-*NPs, while they changed only slightly after incubation with *rac*-NPs (*SI Appendix*, Fig. S32).

To independently confirm that the binding between the three viral proteins and ACE2 was blocked by chiral NPs, we labeled the RBD with fluorescein isothiocyanate (FITC-RBD) and ACE2 with Cy3 (Cy3-ACE2). Testing fluorescence resonance energy transfer of the NP–protein complexes, we mixed FITC-RBD with Cy3-ACE2 at 37 °C for 2 h, and the FITC fluorescence decreased significantly and the Cy3 signal was observed under FITC excitation wavelengths due to the binding between RBD and ACE2. The FITC fluorescence recovered, however, and no Cy3 signal was seen when the chiral NPs were added to the solution containing the RBD and ACE2 complex. These results indicate that the chiral NPs block the binding between viral proteins and ACE2 (*SI Appendix*, Fig. S33).

### Chiral NPs Inhibit SARS-CoV-2 in Living Cells.

A SARS-CoV-2 pseudovirus from a three-plasmid system containing non-Env proteins from vesicular stomatitis virus G, a GFP reporter, and the S protein from SARS-CoV-2 were used to assess the ability of chirality-optimized NPs to inhibit the attachment of pseudovirus to the ACE2 receptor on BEAS-2B. SARS-CoV-2 pseudovirus was labeled with GFP, while the fluorescence of tapered CuS NPs was white (*SI Appendix*, Figs. S34 and S35). Three treatment protocols denoted as treatments 1, 2, and 3 describing both pre-exposure prophylactic and postinfection medication were implemented: treatment 1—the pseudovirus was initially incubated with different concentrations of *L*-NPs for 2 h and then added to BEAS-2B cells and allowed to react for 24 h; treatment 2—mixtures of pseudovirus and *L*-NPs in different concentrations were added to BEAS-2B cells and allowed to react for 24 h; and treatment 3—the pseudovirus was initially incubated with BEAS-2B cells for 1 h, and then, the cells were exposed to different concentrations of *L*-NPs for 24 h.

**Fig. 4.**
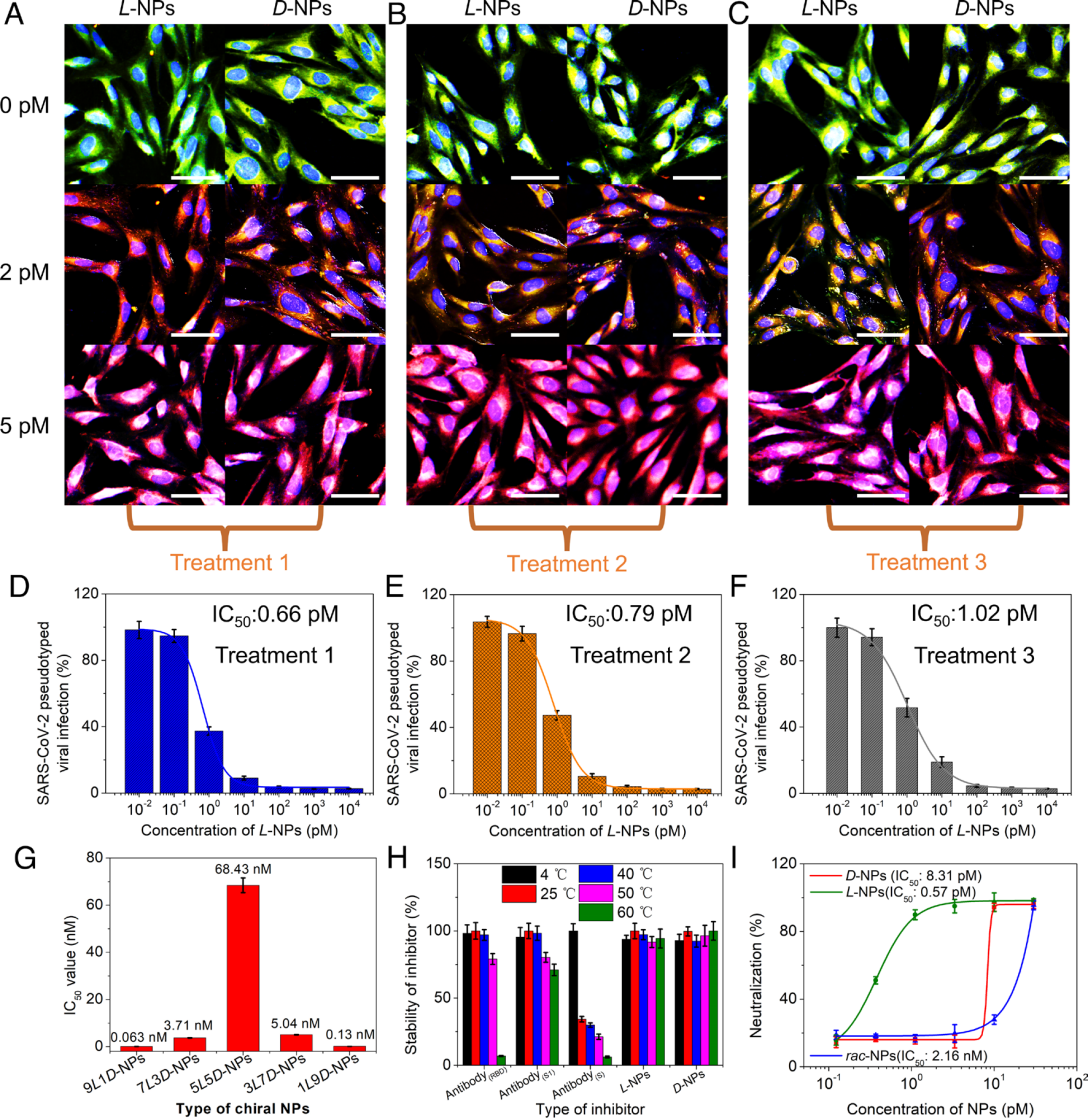
Chiral NPs inhibit the infection of SARS-CoV-2 pseudovirus to BEAS-2B cells. Confocal images of chiral NPs inhibit the infection of SARS-CoV-2 pseudovirus under treatment 1 (*A*), treatment 2 (*B*), and treatment 3 (*C*), respectively, and corresponding quantitative results in treatment 1 (*D*), treatment 2 (*E*), and treatment 3 (*F*), respectively, by flow cytometry. (Scale bars are 50 μm.) (*G*) The IC_50_ values of chiral NPs that modified with different ratios of *L*-Pen to *D*-Pen inhibiting the infection of SARS-CoV-2 pseudovirus to BEAS-2B cell. (*H*) The stability assessment of three antibodies and *L*-NPs stored at different temperatures. Thermal stability analysis of the three antibodies and chiral NPs, three antibodies (anti-RBD antibodies, anti-S1 antibodies, and anti-S antibodies) and chiral NPs were first stored at different temperatures (4 °C, 25 °C, 40 °C, 50 °C, and 60 °C) for 2 h, then using treated antibodies and chiral NPs to evaluate the binding ability to the corresponding antigen (RBD, S1, and *S*). (*I*) Authentic SARS-CoV-2 neutralization results for the chiral NPs were determined via quantizing cell viability using the cck-8 kit. All authentic virus neutralization data were repeated as n = 3 independent replicates. Treatment 1: The pseudovirus was first treated with different concentrations of *L*-NPs for 2 h, and then, pretreated pseudovirus was incubated with BEAS-2B cells for 24 h. Treatment 2: The mixture of pseudovirus and different concentrations of *L*-NPs was coincubated with BEAS-2B cells for 24 h. Treatment 3: The pseudovirus was initially added and incubated with BEAS-2B cells for 1 h, and then, the different concentrations of *L*-NPs were added into cells for another 24 h. Data are presented as mean ± SD (n = 3), with the error bar representing the SD.

As the dose of chiral NPs increased from 0 to 5 pM, their white fluorescence gradually increased. Simultaneously, the green fluorescence of the pseudoviruses gradually disappeared in treatment 1 and treatment 2, and it weakened in treatment 3 (Fig. 4 *A*–*C*). These data indicated that in all cases, the *L-*NPs inhibited the infection of the living cells. IC_50_ values of 0.66 pM (1.44 ng/mL), 0.79 pM (1.72 ng/mL), and 1.02 pM (2.22 ng/mL) were found for treatments 1, 2, and 3, respectively (Fig. 4 *D*–*F* and *SI Appendix*, Fig. S36). *D*-NPs and *rac*-NPs revealed 10 and >10^3^ times higher IC_50_ values than *L-*NPs, respectively (*SI Appendix*, Fig. S36). As a benchmark test for the role of particle chirality, NPs made with various mixtures *L*-Pen or *D*-Pen showed a tendency for lower IC_50_ values as the Pen *L/D* ratio increased (Fig. 4*G* and *SI Appendix*, Fig. S37). Remarkably, the IC_50_ value of chiral NPs inhibiting SARS-CoV-2 pseudovirus–infected cells is about 1,100 times lower than that of commercial antibody (BF04204, Biodragon, China) against the same virus (*SI Appendix*, Fig. S38).

Finally, all types of NPs were tested for their ability to inhibit authentic (as opposed to pseudovirus) wild-type Wuhan 1 virus of SARS-CoV-2. IC_50_ values of 0.57 pM (1.24 ng/mL), 8.31 pM (18.07 ng/mL), and 2.16 nM (4.70 μg/mL) were found for *L*-NPs, *D*-NPs, and *rac*-NPs, respectively, using cell viability assays (Fig. 4*I*). Very importantly, NPs also showed high stability at high temperatures compared with antibodies, a reflection of the inorganic nature of the particles (Fig. 4*H*) and indication of their fundamental advantage compared to antibodies or other treatments based on biological macromolecules, including mRNA.

### Broad-Spectrum Antiviral Activity.

Considering the multiplicity of viral strains that are active even in one season, we tested the activity of chiral NPs against other coronaviruses and the SARS-CoV-2 Omicron variant. The crystal structures of other spike proteins suggested that *L-*NPs could interact similarly and result in the effective specific agglutination of these biomolecules (*SI Appendix*, Fig. S39). Thus, spike proteins from HCoV-HKU1, HCoV-OC43, HCoV-NL63, and SARS-CoV-2 Omicron variant (B.1.1.529) showed strong binding with *L*-NPs, similar to that for SARS-CoV-2 spike (*SI Appendix*, Fig. S39). CD spectra showed conformational changes after incubation with *L*-NPs (*SI Appendix*, Fig. S40) with similar spectral shifts and kinetics as for *S*. *L*-NPs displayed similarly high binding affinities to all four spike proteins, with IC_50_ values of 1.17 pM (2.55 ng/mL) for *S* from HCoV-HKU1, 5.32 pM (11.57 ng/mL) for *S* from HCoV-OC43, 5.49 pM (11.94 ng/mL) for *S* from HCoV-NL63, and 8.56 pM (18.62 ng/mL) for *S* from SARS-CoV-2 Omicron variant (*SI Appendix*, Fig. S39). *D*-NPs displayed IC_50_ values of 10.83 pM (23.56 ng/mL) for *S* from HCoV-HKU1, 53.43 pM (116.23 ng/mL) for *S* from HCoV-OC43, 48.53 pM (105.57 ng/mL) for *S* from HCoV-NL63, and 76.63 pM (166.70 ng/mL) for *S* from SARS-CoV-2 Omicron variant (*SI Appendix*, Fig. S39). Overall, the activity of *D-*NPs against coronaviruses is about 10 times smaller than that for *L-*NPs. *Rac*-NPs displayed IC_50_ values >20 nM (43.51 μg/mL) (*SI Appendix*, Fig. S39), which is >10^3^ times higher than for *L-*NPs.

### *L-*NPs Accelerate the Clearance of SARS-CoV-2 Pseudovirus in Mice.

Animal models for SARS-CoV-2 and other coronaviruses are still being evaluated, and there is no uniform opinion about which is the suitable one (50–52). We used hACE2-expressing transgenic mice to carry out the protection/clearance of viruses by chiral NPs in vivo to answer the fundamental question of the suitability of chiral NPs for treatment of animals or humans.

**Fig. 5.**
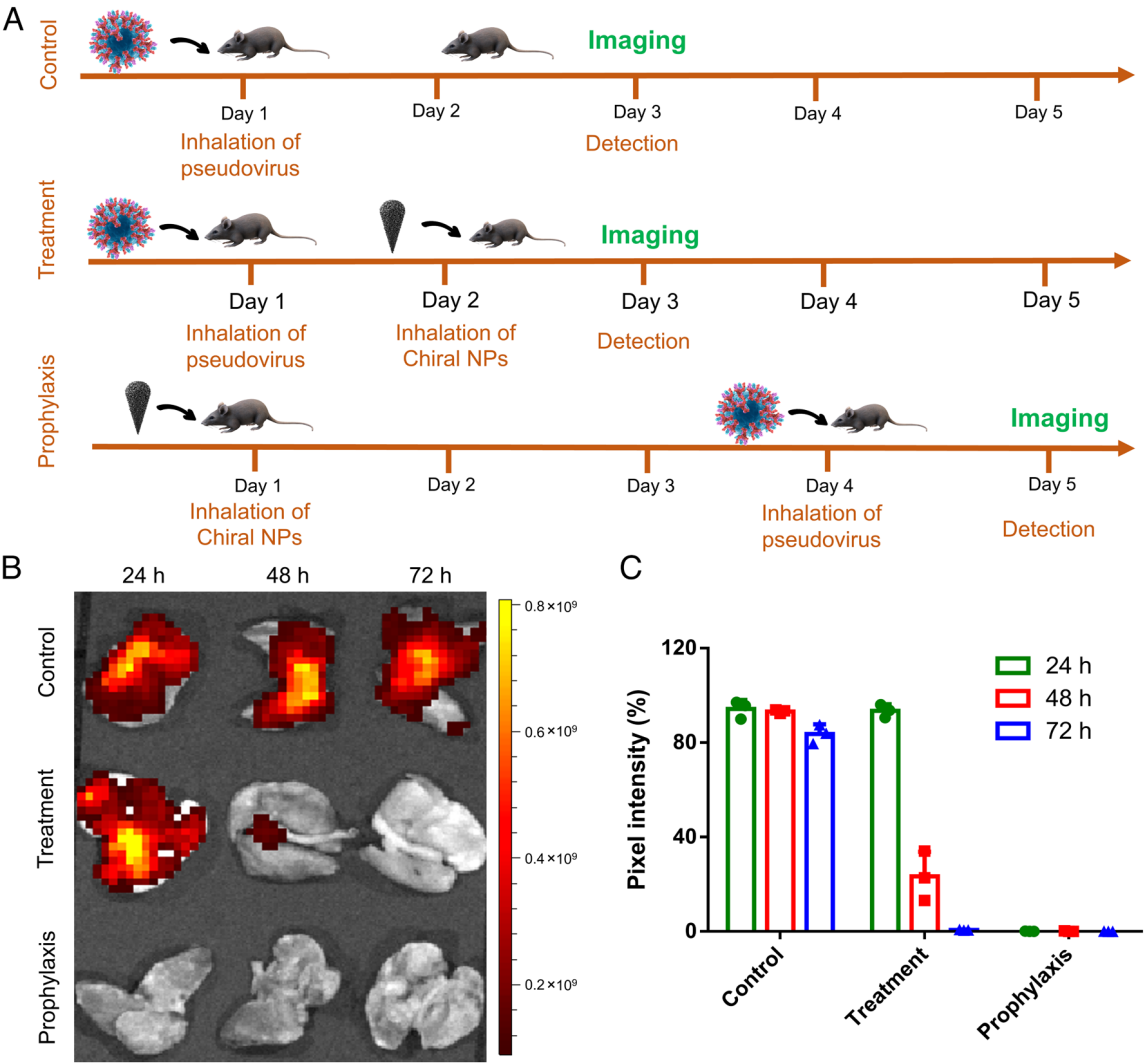
Inhibition of pseudo-type SARS-CoV-2 infection using the hACE2-expressing mouse model. (*A*) Schematic showing the animal study design. (*B*) Representative ex vivo IVIS imaging of lung tissues from mice with various treatments. n = 3 animals per group. (*C*) Quantification of fluorescence intensities of SARS-CoV-2 pseudovirus from the imaging data in (*B*). Data are shown as mean ± SD, n = 3 animals per group.

For this animal model, ACE2 is highly expressed in lung epithelial cells typically targeted by SARS-CoV-2. Following the current practices of treatment and prevention of respiratory diseases, *L*-NPs were included in the aerosol administered to mice using a commercial nebulizer. The fluorescence of *L-*NPs was detected in the lungs for up to 72 h, which was also confirmed by ICP-MS analysis of lung tissue (*SI Appendix*, Fig. S42). The NP residence time is about 1.5 times longer than the residence time for antibodies administered similarly (6). The long residence time for the lungs is suggestive of their potential prophylactic function. To better assess both prophylactic and treatment options for the *L-*NPs, transgenic hACE2 mice were infected with GFP (green fluorescing protein)-SARS-CoV-2 pseudovirus (Fig. 5*A* and *SI Appendix*, Figs. S41 and S42). In the prophylaxis group, hACE2 mice resisted SARS-CoV-2 infections, after inhaling the *L-*NPs for up to 3 d when compared with the control group. In the treatment group, 95% of the infection of pseudovirus in the lungs was cleared after treatment with *L-*NPs (Fig. 5 *B* and *C*). Murine serological indicators and hematoxylin and eosin staining showed that the *L-*NPs displayed biocompatibility with mammalian cells with no observed toxicity (53) for concentrations specific to high antiviral activity (*SI Appendix*, Figs. S42 and S43). Cytokine analysis suggested that *L-*NPs inhalation did not elevate proinflammatory cytokines with respect to the control group (*SI Appendix*, Figs. S42 and S43). We also tested the antiviral activity of NPs after storing them at temperatures up to 60 °C for 24 h, conditions that typically “killed” the biological activity of biomacromolecules exemplified by antibodies. The NPs retained the ability to agglutinate viral proteins indicating temperature tolerance hardly achievable for high-molecular-weight biologics (54).

## Conclusions

Chiral NPs with complex twisted shapes can serve as effective inhibitors of different variants of coronaviruses due to their strong binding to spike proteins. Efficient agglutination of viral particles enables treatment of antibody-resistant viral strains. Inherent structural variations of NPs retaining the geometrical match with spike proteins may contribute to broad-spectrum activity against coronaviruses. Based on the observed multistrain antiviral activity, a dedicated study of mutation resistance including demographic history and recombination probabilities will be necessary for further assessment of clinical prospects. The thermal stability of NPs combined with antibody-like curative potential opens a possibility of their utilization as rapid-deployment antiviral at the onset of pandemics.

## Methods

### Clearance of Pseudo-Typed SARS-CoV-2 In Vivo.

Human ACE2 (hACE2)-exprssing transgenic (Tg) male mice were randomly divided into three groups (control group, prophylactic group, and treatment group) for the first intratracheal administration of SARS-CoV-2, pseudovirus (1.0 × 10^6^ TU per body) was introduced into the control and treatment groups. Twenty-four hours later, *L*-NPs (5 nM per body) and an equal volume of PBS were inhaled into the treatment and control groups. After 24 h, the mice were killed, and all major organs were collected for further analysis. For the prophylactic group, *L*-NPs were first inhaled by the mice, and then, 72 h later, the pseudoviruses were inhaled. After 24 h, the mice were killed, as shown in Fig. 5*A*.

## Supplementary Material

Appendix 01 (PDF)

Movie S1. 3D reconstruction of achiral and chiral tapered NPs from ADF - STEM images. The solid spheres are locations of atomic columns based on image intensities in ADF-STEM images.

Movie S2. 3D tomography reconstruction of L-NPs incubated with Spike proteins of SARS-CoV-2 for 2 h.

Movie S3. 3D tomography reconstruction of D-NPs incubated with Spike proteins of SARS-CoV-2 for 2 h.

## Data Availability

Spectroscopy and images data have been deposited in EuropePMC (DOI: https://doi.org/10.21203/rs.3.rs-2501398/v1) (54). All other data are included in the manuscript and/or supporting information.
