# Supplementary material for: Tapered chiral nanoparticles as broad-spectrum thermally stable antivirals for SARS-CoV-2 variants
Source: Proc Natl Acad Sci U S A. 2024 Mar 19;121(13):e2310469121. doi: 10.1073/pnas.2310469121 (PMC10990083; doi:10.1073/pnas.2310469121)
Supplement: Supplementary file 1 — Appendix 01 (PDF) [file pnas.2310469121.sapp.pdf]

PNAS

Supporting Information for

Tapered Chiral Nanoparticles as Broad Spectrum Thermally Stable Antivirals for SARS-CoV-2 Variants

Rui Gao, Xinxin Xu, Prashant Kumar, Ye Liu, Hongyu Zhang, Xiao Guo, Maozhong Sun, Felippe Mariano Colombari, André F. de Moura, Changlong Hao, Jessica Ma, Emine Sumeyra Turali Emre, Minjeong Cha, Liguang Xu, Hua Kuang, Nicholas A. Kotov, Chuanlai Xu

Liguang Xu, Nicholas A. Kotov
Email: xlg@jiangnan.edu.cn, kotov@umich.edu

This PDF file includes:

- Materials and Methods
- Figures S1 to S43
- Tables S1 to S3
- Legends for Movies S1 to S3
- SI References

Other supporting materials for this manuscript include the following:

- Movies S1 to S3

Materials

Copper **(II)** acetate monohydrate (C4H6CuO4·H2O, 99.95%), copper nitrate trihydrate (Cu(NO3)2·3H2O, 99.99%), 1-octadecene (C18H36, 99.5%), 1-dodecanethiol (1-DDT, 99.5%), tert-dodecylmercaptan (tert-DDT, 98%), trioctylphosphine oxide (TOPO, 98%), sodium citrate dihydrate (C6H5Na3O7·2H2O, 99%), zinc acetate dihydrate (C4H6O4Zn·2H2O, 99.99%), potassium hydroxide (KOH, 99.99%) tetraethyl orthosilicate (TEOS, 99.99%), sodium borohydride (NaBH4, 98%), chloroauric acid (HAuCl4·3H2O), SDS solution (CH3(CH2)11OSO3Na, 99%), sodium hydroxide (NaOH, 99.9%), N,N-dimethylformamide (DMF, 99.9%), trichloromethane ($CHCl_3$, AR), ethanol (C2H6O, 99.5%), methanol (CH4O, 99.9%), isopropyl alcohol (CH3CH**(OH)**CH3, 99%), glycerin (99%), glycol (99%), sodium periodate (99.5 %), ammonium sulfate (99%), ammonia solution (H5NO, 25-28%), tartaric acid (TA, 99%), malic acid (MA, 99%), Cy3, fluorescein5-isothiocyanate (FITC, 90%), Cy5, phosphotungstic acid hydrate (H3PO40W12·XH2O, 90%) and GSH were all purchased from Sigma-Aldrich (St. Louis, MO, USA). The two enantiomers of Pen were purchased from TCI Co., Ltd (Taipei City, Taiwan). SARS-CoV-2 pseudo-viruses were purchased from Suzhou Jinweizhi Biotechnology Co., LTD (Suzhou, China) with further purification by ultrafiltration (100 kDa). 4% paraformaldehyde Fix Solution, RIPA lysis buffer IV and poly-L-lysine were purchased from Shanghai Sangon Biotech., Ltd (Shanghai, China). All cell media and buffer solutions were purchased from Thermo Fisher Scientific (Shanghai, China). CCK-8 reagent was purchased from Beyotime Biotechnology (Shanghai, China). ALT, BUN, AST, and CRE tests were carried out in the Fourth People's Hospital of Wuxi City. IL-10, IL-12, IL-1β, IFN-α, IFN-β, and IFN-γ ELISA kits were purchased from Jiangsu Jingmei Technology Co., Ltd (Yancheng, China). Milli-Q water (>18 MΩ cm) was used in all experiments. HEK293 cell and BEAS-2B cell were purchased from Kunming Cell Bank, Chinese Academy of Sciences. Pen ligands were purified using recrystallization. Specifically, two enantiomers of penicillamine were fully dissolved in a minimal amount of 0.1 M HCl. Then the solutions of the enantiomers were quickly transferred into a 4 °C water bath for crystallization. This procedure was repeated three times.

Methods

Characterization

Quantitative CD spectra were processed using OriginLab® software from Applied Photophysics Ltd. Raman spectra were obtained from confocal Raman microscope (LabRAM HR Evolution, HORIBA Jobin Yvon S.A.S.) with a 633 nm laser as the excitation wavelength. The fluorescence spectra were obtained with an FLS980 fluorescence spectrophotometer (Edinburgh Instruments, Edinburgh, UK) at a scanning speed of 0.2 nm/s. Transmission electron microscopic **(TEM)** images were obtained with a JEOL JEM-2100 operating at an acceleration voltage of 200 kV and 120 kV for biological samples. Cryo-TEM images were obtained with a Talos F200C operating at an acceleration voltage of 200 kV. Liquid chromatography-mass spectrometry was performed using a LC-MS system (Waters, UK). The high-angle annular dark-field scanning TEM and EDX elemental mapping images were created using a Tecnai G2 F30 S-Twin field-emission TEM, operating at 200 kV. Flow cytometry data were analyzed by FlowJo10.3 and GraphPad prism software (Becton, Dickinson and Company, USA). 3D geometric morphology was performed with an FEI 300 kV Titan Krios Cryo electron microscope equipped with a Gatan Ultrascan 4000 (model 895) 16-megapixel CCD and reconstructed with the Origin software. Mouse organ images obtained from IVIS Lumina XRMS Series III (Platinum Elmer, USA). XRD patterns were obtained on a Bruker XRD system with Cu-Kα radiation operated at 30 kV. Inductively coupled plasma mass spectrometry results were obtained from an ICAP TQ (Thermo Fisher, Germany). XPS measurements were used to verify the composition of the chiral NPs and carried out with an AXIS Supra by Kratos Analytical Inc **(UK)** using monochromated Al-Kα radiation (hv = 1486.6 eV, 150W) as the X-ray source. Fourier Transform Infrared Spectroscopy (FT–IR) measurements were carried out with a Nicolet 6700 FT-IR spectrometer (Thermo Scientific). Thermodynamic effects were measured with a Nano ITC Low Volume isothermal titration calorimeter (TA Instrument, USA). Confocal images were obtained with a Leica TCS SP8 confocal fluorescence microscope (Leica Microsystems, Wetzlar, Germany). Size distributions and zeta potential were determined using a Zetasizer Nano ZS system (Malvern, USA). Two-dimensional multifunctional small Angle X-ray scatterer was performed with Xeuss 3.0HR (Xenocs, France).

Synthesis of D-NPs, L- NPs, rac-NPs, Cit-NPs, GSH-NPs, MA-NPs and TA-NPs

The syntheses of D-NPs, L-NPs, and rac (racemic)-NPs were carried out by a modified version of the previously published method(1). CuS NPs were synthesized by heating a mixture of Cu(Ac)2 (0.2 g) and 1-DDT (50 mL) to 200 ℃ in TOPO (1.74 g) under N2 atmosphere. After 4 h, oil-soluble NPs were purified by centrifugation with the addition of CHCl3 and centrifuged three times and then resuspended in CHCl3 again. Then, water-soluble NPs were obtained by adding a surface ligand solution (100 mg/mL in a mixed solvent consisting of ultrapure water and ethylene glycol) followed by ultrasonication for 2 h. The production of the L-NPs involved adding L-Pen solution to the NPs resuspended in CHCl3. Similarly, D-NPs were produced by adding D-Pen solution into the NPs resuspended in CHCl3. Rac-NPs were produced by equal amounts of D-Pen and L-Pen added to the NPs resuspended in CHCl3. Cit-NPs were produced by adding sodium citrate solution (100 mg/mL) to the NPs resuspended in CHCl3. GSH-NPs were produced by adding GSH solution (25 mg/mL) to the NPs resuspended in CHCl3. MA-NPs were produced by adding malic acid solution (100 mg/mL) to the NPs resuspended in CHCl3. Finally, TA-NPs were produced by adding tartaric acid solution (100 mg/mL) to the NPs resuspended in CHCl3.

Synthesis of 8 nm CuS NPs with Pen as surface ligands

The 8 nm CuS NPs were synthesized by heating 1-DDT solution and Cu(Ac)2 under a N2 atmosphere (2). Typically, Cu(Ac)2 (0.13 g) was dispensed into 30 mL of 1-DDT under magnetic stirring in the presence of N2 gas and then heated for 20 min to 200 °C. Oil-soluble NPs were purified by centrifugation with the addition of CHCl3 and recovered three-times and resuspended in CHCl3. Then water-soluble NPs were obtained by adding ligand solution (100 mg/mL) with ultrasonic treatment for 2 h. The L-CuS NPs (8 nm) were produced by adding L-Pen solution into the above-mentioned NPs that were resuspended in CHCl3 and the D-CuS NPs (8 nm) were produced by adding D-Pen solution into the above-mentioned NPs that were resuspended in CHCl3.

Synthesis of 25 nm CuS NPs with Pen as surface ligands

The 25 nm CuS NPs were synthesized by heating 1-DDT solution and Cu(Ac)2 under a N2 atmosphere (3). Typically, of Cu(Ac)2 (0.13 g) was dispersed in 30 mL of 1-DDT under magnetic stirring in the presence of nitrogen gas and then heated to 200°C for 60 min. Oil-soluble NPs were purified by centrifugation with the addition of CHCl3 three times and then resuspended in CHCl3. The water-soluble NPs were obtained by adding ligand solution (100 mg/mL) with ultrasonic treatment for 2 h. The L-CuS NPs (25 nm) were produced by adding L-Pen solution into the above-mentioned NPs that were resuspended in CHCl3 and D-CuS, and NPs (25 nm) were produced by adding D-Pen solution into the above-mentioned NPs that were resuspended in CHCl3.

Synthesis of Cu1.94S nanospheres with Pen as surface ligands

The spherical Cu1.94S NPs with a diameter of 3 nm with Pen as surface ligand were synthesized according to previously reported methods with brief modifications (2). 1-DDT (4.5 mL) and ethanol (10 mL) were mixed in a glass vial and a separate solution containing 1.1 g of Cu(NO3)2·3H2O and 5 mL of H2O was produced. The two solutions were mixed and vigorously stirred to form a yellow-white suspension. The suspension was collected by centrifugation, washed two times with H2O and ethanol (volume ratio of 1:2) to remove the excess reagents, and then dried in a vacuum desiccator overnight to purification. A yellow-white powder was obtained by drying in a 60 ℃ vacuum dryer for 1 week, and 20 mg of powder was placed in a glass vial and heated for 30 min to 200 ℃. The dark-brown liquid was collected and dispersed in CHCl3. Purified nanodots were obtained by centrifuging at 10000 rpm for 20 min. This purification process was repeated three times. Then water-soluble NPs were obtained by addition of a Pen solution (100 mg/mL) and ultrasonic treatment for 2 h at pH 9.

Synthesis of CuS nanorods with Pen as surface ligands

The CuS nanorods (NRs) with a length of 45 nm and diameter of 4 nm with Pen as the surface ligand were synthesized according to previously reported methods with small modifications (4). Copper acetate (0.4 g) was dissolved in 1-octadecene (10 mL) and TOPO (4 g) was added. The reaction was stirred at room temperature for 30 min under a N2 atmosphere. Then, the mixture was heated to 180 ℃. At this point, tert-DDT (7.5 mL) was injected, and the reaction was allowed to

proceed for 10 min. The resulting product was then cooled to room temperature, washed several times with ethanol, and redissolved in CHCl3. Then, water-soluble NRs were obtained by the addition of Pen solution (100 mg/mL) followed by ultrasonic treatment for 2 h in the presence of SDS (10 mM).

Synthesis of SiO2 Nanospheres with Pen as the surface ligands
The spherical SiO2 NPs with Pen as surface ligand were synthesized according to previous methods with brief modifications (5). L-Pen (15 mg) was dissolved into 4 mL H2O and after stirring for 5 min, ethanol (4 mL) was added for another 5 min under stirring. TEOS (1 mL) and ammonia solution (100 μL) were added into the above solution and then the mixture was heated for 20 min at 60 °C. Finally, this reaction was left at room temperature for 10 h and the purified L-Pen modified SiO2 NPs were centrifuged (3000 rpm for 10 min).

Synthesis of ZnO pyramids with Pen as surface ligands
The ZnO pyramids with Pen as the surface ligands were synthesized according to previous methods with brief modifications (6). In a typical synthesis, C4H6O4Zn·2H2O (100 mg) and KOH (20 mg) were added into 1 mL of isopropyl alcohol, 4 mL H2O and L-Pen (15 mg) in a 20 mL round-bottom flask. The mixture was then magnetically stirred for 72 h at room temperature and the white precipitates were separated by centrifugation (7000 rpm 10 min).

Synthesis of spherical Au NPs with Pen as surface ligands
The spherical Au NPs with Pen as surface ligands were synthesized according to previous methods with brief modifications (7, 8). In a typical synthesis, L-Pen (7.5 mg) was added to a 20 mL three-necked flask containing 10 mL H2O and 60 mg of HAuCl4·3H2O under vigorous stirring for 2 min at room temperature and then quickly transferred to an oil bath (95 ℃) and heated for 30 min. After the heated solution cooled to room temperature it was placed in a refrigerator to mature for 72 h. Then, 200 μL of the above-mentioned matured solution and 3 mL of H2O were added into a 10 mL three-necked flask containing sodium borohydride (1.89 mg), trisodium citrate (14.7 mg), L-Pen (15 mg) and stirred vigorously for 2 h. Finally, the precipitates were separated by centrifugation (9000 rpm 10 min).

Assembly of Supraparticles (SPs)
Supraparticles formed by RBD + chiral NPs were obtained by mixing the RBD (0.3 mg/mL, 50 μL) in PBS (0.01 M, pH 7.4) with chiral NPs (2 nM) for 6 h at 37 ℃. SPs (S1 + chiral NPs) were obtained by mixing the S1 (0.3 mg/mL, 50 μL) in PBS (0.01 M, pH 7.4) with chiral NPs (2 nM) for 6 h at 37 ℃. SPs (S + chiral NPs) were obtained by mixing the S (0.3 mg/mL, 50 μL) in PBS (0.01 M, pH 7.4) with chiral NPs (2 nM) for 6 h at 37 ℃.

TEM imaging of chiral NPs bound to SARS-CoV-2 pseudovirus
SARS-CoV-2 pseudovirus (100 μL, 1.0 × 107 TU/mL) was first mixed with 4% paraformaldehyde fixation solution for 30 min then the 4% paraformaldehyde was removed by centrifugation (8000 rpm, 20 min, twice) and re-suspended in DPBS. Equal volumes of NPs (1 pM) and pseudovirus solution were mixed at room temperature for 6 h and finally, the chiral NP - pseudovirus complexes was collected by centrifugation (8000 rpm, 10 min). Next, 8 μL of purified sample was dropped onto glow discharge grids (200 mesh carbon-coated copper grids) for 30 min and rinsed with three drops of PBS (10 mM, pH 7.4). Then the rinsed grids were floated with phosphotungstic acid solution (10 μL, 10 mM, pH 6.0) for another 5 min, then blotted dry with filter paper. Finally, the images were captured by TEM (JEOL JEM-2100) operating at an acceleration voltage of 120 kV.

TEM imaging of SARS-CoV-2 viral proteins
SARS-CoV-2 viral proteins (20 μL of 0.05 mg/mL) were firstly fixed with a 4% paraformaldehyde solution and adsorbed onto glow discharge grids (300 mesh carbon-coated copper grids) for 5 min and rinsed with three drops of PBS (10 mM, pH 7.4). The grids were then floated onto a phosphotungstic acid solution (10 μL, 10 mM, pH 6.0) for another 5 min, and fixed with glutaraldehyde (0.25%) and blotted dry with filter paper. The images were captured by a TEM (JEOL JEM-2100) operating at an acceleration voltage of 120 kV.

Molar concentrations of NPs
1 mL of aqueous dispersion of chiral NPs prepared as described above was centrifuged at 8000 rpm for 10 min (3 times). Then, the precipitate was dried in a vacuum drying oven at 60 ℃ for 96 h to obtain the dry weight of the dispersed material (Mt, the unit is g). The shape of the tapered CuS NPs was approximated as a cone. Then, the volume of a single chiral NP (v_NP) is v_NP=1/3 πR^2 h, where R is the radius of the cone (5 nm) and h is its height (30 nm). The density of chiral CuS NPs was assumed to be that of bulk CuS ρ = 4.6 g/mL, hence the average mass of single NP (m_NP) is m_NP=1/3 πR^2 h ρ = 3.61×10-18 g. Therefore, the number of NPs dispersed in 1 mL is N_NPs=M_t/m_NP =M_t/(3.61 × 10^(-18))= Mt ×2.77×1017. Then, the number of moles of NP dispersed in 1 mL is n_NPs=N_NPs/NA , where NA is the Avogadro's constant equal to 6.022×1023, that leads to n_NPs=(M_t ×2.77×10^17)/NA = Mt × 4.60 ×10-7 mol. The molar concentration of chiral NPs (C_NPs,mol/L) can be calculated as C_NPs=n_NPs/V , where V = 1 × 10-3 L, and thus, C_NPs=n_NPs/V =(M_t × 4.60 ×10^(-7) mol)/(1×10^(-3) L)= Mt ×4.60 ×10^(-4) mol/L .

Expression of recombinant RBD
The RBD of SARS-CoV-2 was expressed using Expi293F (Thermo Fisher) (9). The RBD from Arg319 to Phe541 with an N-terminal S1 signal peptide from Met1 to Cys15 for secretion, and C-terminal with a 10× His tag for purification, was inserted into the pCMV3 vector (Sino Biological). The plasmid was transfected into Expi293F cells at a density of 4×106 cells/mL in a constant temperature shaker (37 °C, 5% CO2 and 160 rpm) using Sinofection (Sino Biological, STF02). The supernatant from the cell culture containing the secreted RBD was collected after 72 h. Then, after concentrating, the RBD was buffer exchanged to HBS (10 mM HEPES, pH 7.2, 150 mM NaCl). The RBD was then purified using a Ni-NTA resin (Sangon) and eluted with 500 mM imidazole in HBS buffer. The RBD was further purified by size-exclusion chromatography using a Superose 6 10/300 column (GE Healthcare) in a buffer containing 2 mM Tris, pH 8.0, 200 mM NaCl and 0.02% NaN3.

Expression of recombinant S1 protein
The S1 of SARS-CoV-2 was expressed using the Expi293F (Thermo Fisher) (9). The S1 from Val16 to Arg685 with an N-terminal S1 signal peptide from Met1 to Cys15 for secretion, and C-terminal with a 10× His tag for purification was inserted into the pCMV3 vector (Sino Biological). The plasmid was transfected into Expi293F cells at a density of 4×106 cells/mL in a constant temperature shaker (37 °C, 5% CO2 and 160 rpm) using Sinofection (Sino Biological, STF02). The supernatant from the cell culture containing the secreted S1 was collected after 72 h. After concentrating, the S1 was buffer exchanged to HBS (10 mM HEPES, pH 7.2, 150 mM NaCl). The S1 was then purified using Ni-NTA resin (Sangon) and eluted with 500 mM imidazole in HBS buffer. The S1 was further purified by size-exclusion chromatography using a Superose 6 10/300 column (GE Healthcare) in buffer (2 mM Tris, pH 8.0, 200 mM NaCl and 0.02% NaN3).

Expression of recombinant S-protein
The SARS-CoV-2 S-protein HexaPro Plasmid was prepared according to the reported literature (9) and then transfected into Expi293F cells (Thermo Fisher) using Sinofection (Sino Biological, STF02). The supernatant from the cell culture containing the secreted S-protein was harvested by centrifugation after four days. Then after concentrating, the S-protein was buffer exchanged to HBS (10 mM HEPES, pH 7.2, 150 mM NaCl). S-protein was then purified using a Ni-NTA resin (Sangon), eluted with 500 mM imidazole in HBS buffer, and then was further purified by size-exclusion chromatography using a Superose 6 10/300 column (GE Healthcare) in buffer (2 mM Tris, pH 8.0, 200 mM NaCl, and 0.02% NaN3).

Expression of recombinant S proteins of HCoV-HKU1, HCoV-OC43, HCoV-NL63, and SARS-CoV-2 Omicron (B.1.1.529) variant
The four spike proteins were expressed using the Expi293F (Thermo Fisher). For HCoV-HKU1 spike, the S from Met1 to Asn1276 segment was selected for for secretion, and C-terminal with a

10× His tag for purification. The R752 to R756 was replaced with G752 to G756 against cleavage by Furin (10). For HCoV-OC43 spike, the S from Met1 to Asn1263 segment was selected for peptide for secretion, and C-terminal with a 10× His tag for purification. R754 to G758 was replaced with G754 to G758 against cleavage by Furin (11). For HCoV-NL63 spike, the S from Met1 to Asn1291 with an own signal peptide for secretion, and C-terminal with a 10× His tag for purification (12). For SARS-CoV-2 Omicron (B.1.1.529) variant spike, the S from Met1 to Asp1359 with an own signal peptide for secretion, and C-terminal with a 10× His tag for purification (13). The four S proteins HexaPro Plasmid were purchased from Sangon Biotech (Shanghai) and then transfected into Expi293F cells (Thermo Fisher) using Sinofection (Sino Biological, STF02). The supernatant from the cell culture containing the secreted S proteins were harvested by centrifugation after four days. Then after concentrating, the S proteins were buffer exchanged to HBS (10 mM HEPES, pH 7.2, 150 mM NaCl). The S proteins were then purified using a Ni-NTA resin (Sangon) and eluted with 500 mM imidazole in HBS buffer. The S proteins were further purified by size-exclusion chromatography using a Superose 6 10/300 column (GE Healthcare) in buffer (2 mM Tris, pH 8.0, 200 mM NaCl and 0.02% NaN3).

Expression of recombinant N-protein

The N-protein of SARS-CoV-2 was expressed using Expi293F cells (Thermo Fisher) (14). The N-protein from Met1 to Ala419 with an N-terminal IL2 signal peptide from Met1 to Ser20 was used for secretion and the C-terminal with a 10×His tag used for purification and inserted into pCMV3 vector (Sino Biological). The plasmid was then transfected into Expi293F cells at a density of 4×106 cells/mL in a constant temperature shaker (37 °C, 5% CO2 at 160 rpm) using Sinofection (Sino Biological, STF02). The supernatant from the cell culture was collected after 72 h. After concentrating, N-protein was buffer exchanged to HBS (10 mM HEPES, pH 7.2, 150 mM NaCl). N-protein was then purified using Ni-NTA resin (Sangon), then eluted with 500 mM imidazole in HBS buffer, and after that, N-protein was further purified by size-exclusion chromatography using a Superose 6 10/300 column (GE Healthcare) in buffer (2 mM Tris, pH 8.0, 200 mM NaCl and 0.02% NaN3).

Expression of recombinant ACE2

ACE2 was expressed using Expi293F cells (15) (Thermo Fisher) and ACE2 from Gln18 to Ala614 with an N-terminal ACE2 signal peptide from Met1 to Ala17, was used for secretion, and a C-terminal with a 10×His tag for purification were inserted into pCMV3 vector (Sino Biological). The plasmid was transfected into Expi293F cells at a density of 4×106 cells/mL in a constant temperature shaker (37 °C, 5% CO2 and 160 rpm) using Sinofection (Sino Biological, STF02). The supernatant from the cell culture containing the secreted ACE2 was collected after 72 h. After concentrating, the ACE2 was buffer exchanged to HBS (10 mM HEPES, pH 7.2, 150 mM NaCl) and purified using Ni-NTA resin (Sangon) and eluted with 500 mM imidazole in HBS buffer. The ACE2 was further purified by size-exclusion chromatography using a Superose 6 10/300 column (GE Healthcare) in buffer (2 mM Tris, pH 8.0, 200 mM NaCl and 0.02% NaN3).

Expression of ACE2 in HEK293 cells

A cell line, in which full-length ACE2 was consistently overexpressed on the cell surface (15) was obtained by transfection of the plasmid pCMV-ACE2 into HEK293 cells using Lipofectamine 2000 (Invitrogen) according to the instructions of manufacturer. Three days post-transfection, cells were treated with 400 µg/mL hygromycin to screen transfected colonies. Living cells were further introduced to limited dilution for single colony selection. Generated cells were characterized using anti-ACE2 antibody.

Transient expression of the RBD, S1, and S proteins in HEK293 cells

The genes of RBD, S1, and S, without the region of signal peptide, were constructed in pCMV vector, respectively (16). Each of them were transfected into HEK293 cells using Lipofectamine 2000. Forty-eight hours post-transfection, cells were harvested and seeded into 35 mm Petri dishes for use.

Preparation of FITC-labeled antibodies

FITC (0.35 mg) was dissolved into N,N-dimethylformamide (400 μL) and the anti-RBD antibody was dissolved in carbonate buffer solution (CBS 0.02 M, 3 mL). The two solutions were then mixed and stirred for 24 h at 4 ℃ in the dark. The produced FITC-labeled anti-RBD antibody was purified by dialysis for 3 days at 4 ℃ and the dialysate changed every 3 h to remove unreacted small molecules. The FITC-labeled anti-S1 antibody, FITC-labeled anti-S antibody and FITC-labeled anti-ACE2 antibody were prepared using the same method.

Preparation of FITC-labeled antigens

FITC (0.35 mg) was dissolved into N,N-dimethylformamide (400 μL) and the RBD was dissolved into CBS (0.02 M, 3 mL). The two solutions were then mixed and stirred for 24 h at 4 ℃ in the dark. The produced FITC-labeled RBD was purified by dialysis for 3 days at 4 ℃ and the dialysate changed every 3 h to remove unreacted small molecules.

Preparation of Cy3-labeled ACE2

Cy3 (0.364 mg) was dissolved into N,N-dimethylformamide (400 μL) and the ACE2 was dissolved into CBS (0.02 M, 3 mL). The two solutions were mixed and stirred for 24 h at 4 ℃ in the dark. The produced Cy3-labeled ACE2 was then purified by dialysis for 3 days at 4 ℃ and the dialysate was changed every 3 h to remove unreacted small molecules.

Preparation of Cy5-labeled anti-ACE2 antibody

Cy5 (0.364 mg) was dissolved into N,N-dimethylformamide (400 μL) and the anti-ACE2 antibody was obtained from immunogenic animals and dissolved into CBS (0.02 M, 3 mL). The two solutions were then mixed and stirred for 24 h at 4 ℃ in the dark. The produced Cy5-labeled ACE2 was purified by dialysis for 3 days at 4 ℃ and the dialysate was changed every 3 h to remove unreacted small molecules.

Preparation of HRP-conjugated antibodies

In a brown reaction vessel, HRP (0.2 mL, 10 mg/mL) was added to sodium periodate (0.2 mL, 0.06 M) for 30 min at 4 ℃. Next, glycol (0.2 mL, 0.16 M) was added for 1 h at room temperature to eliminate the redundant sodium periodate. Then 2 mg of antibody was added, and the solution was set to pH 9 using CBS buffer (0.05 M) for 24 h at 4 ℃. Sodium borohydride was then quickly added (0.08 mg/mL, 5 mL) for 2 h at 4 ℃ and an equal volume of saturated ammonium sulfate solution was added for 1 h at 4 ℃ and the HRP-conjugated antibodies were purified by centrifugation (5000 rpm, 10 min). The precipitate was then collected and re-suspended in PBS (0.1 M) and dialyzed in the dark for 3 days and the purified HRP-conjugated antibodies were stored with 50 % glycerin at −20 ℃.

TEM imaging of chiral CuS NPs

NPs were dropped cast for TEM measurements on TEM grids (ultrathin carbon film on holey carbon support film, 200 mesh Cu, Ted Pella). The grids were sealed under vacuum at ~ 40 ℃ in an in-house developed decontamination chamber for > 5 hours before insertion into the TEM to avoid contamination buildup. Annular dark field scanning transmission electron microscopy (ADF-STEM) was performed using Thermo Fisher Scientific Talos F200X G2 S/TEM equipped with a Super-X EDX detector. STEM probe was aligned at 200 keV electron voltage and 10.5 mrad semi-convergence angle at spot size 6 to minimize electron beam damage. Images were acquired with dwell time 2 μs, at a camera length of 98 mm, corresponding to collection angles of bright-field **(BF)** detector as 8 mrad, dark field (DF2) detector 2 as 11-19 mrad, dark field detector 4 (DF4) as 22-52 mrad and high-angle ADF **(HAADF)** detector as 56-200 mrad. Pixel size varied between 0.23 Å × 0.23 Å to 0.081 Å × 0.081 Å for acquired atomic resolution images.

ADF-STEM image analysis

Acquired ADF-STEM images were analyzed in MATLAB. The individual local peaks corresponding to atom locations were estimated for NPs without ligands (0.23 Å/pix) and with L-Pen (0.08 Å/pix) using an estimated atom radius of 5 pix and 15 pix with minimum distance between atoms set as 15 pix and 30 pix, respectively. Peaks identified outside the NP were manually deleted. Using the initially determined location of atoms, a 2D gaussian function was fitted across each projected atomic column and the intensities of each column were plotted as scattering cross-sections in the histogram. To generate a 3D reconstruction from the ADF-STEM image, it is assumed that the bright columns arise due to scattering from Cu atoms in CuS structure. Using integration classification likelihood criterion in combination with gaussian mixture model estimation, the number of atoms and their locations can be found. An upper limit of 45 was given for the number of atoms based on the rotational symmetry of the thickest region of the NP. Gaussian mixture models are fitted up to the maximum number of atoms to histogram of scattering cross-sections (17-20). The corresponding thickness maps are generated from the images and the reconstruction is shown (Supplementary video). The displacement maps account for strain in lattice from an ideal projected unit cell calculated from FFT.

***Multislice* simulations**

Bright-field TEM images were simulated using the Multislice algorithm (21). Covelite (CuS) unit cell with lattice parameters as a,b,c = 3.796 Å, 3.796 Å, 16.382 Å, α ,β ,γ = 90°, 90°, 120° was symmetry transformed into a unit cell with a,b,c = 6.5748 Å, 16.382 Å, 3.796 Å, α ,β ,γ = 90°, 90°, 90°. The transformation was performed to orient the crystal along the c-direction. Supercell of lateral dimension 150 Å × 150 Å and varying thickness along c-direction, with 2048 × 2048-pixel size was used for simulating the projected atomic potential with a probe of 200 keV, Cs3 = 2 mm, Objective aperture size = 10 mrad and astigmatism = 0. Defocus and tilt maps were simulated in the range of -200 to 200 nm and 0 to 100 mrad about a- and b-axis.

Detection of SARS-CoV-2 mimics by ELISA

RBD detection: RBD was diluted to 0.3 μg/mL in CBS, (0.1 M, pH 9.7) and 100 μL was added into a 96-well plate. Then different concentrations of chiral NPs (50 μL of 0.01 pM, 0.1 pM, 1 pM, 10 pM, 100 pM, 1000 pM, and 10000 pM) were added into the wells and incubated for 2 h at 37 ℃ (0.1 M PBS was used as control). The blocking buffer (200 μL) was added after three washes (washing buffer: phosphate-buffered saline, 0.05% Tween-20) and incubated for 2 h at 37 ℃. After repeating the washing steps, HRP-conjugated anti-RBD antibody (100 μL, 0.5 μg) was added into every well and incubated for 1 h at 37 ℃. After a final washing step, a colorimetric signal developed due to the enzymatic reaction of HRP with the chromogenic substrate, TMB after incubation for 15 min at 37 ℃. Finally, 50 μL of stop buffer was added to terminate the reaction, and an absorbance reading at 450 nm was acquired using a microplate reader (BioTek). For rac-NPs, the concentration is set to 0.1 nM, 1 nM, 10 nM, 100 nM, 1000 nM, 10000 nM, and 100000 nM.

S1 protein detection: S1 was diluted to 0.3 μg/mL in CBS (0.1 M, pH 9.7) and 100 μL was added to each well of a 96-well plate. Next, different concentrations of NPs (50 μL of 0.01 pM, 0.1 pM, 1 pM, 10 pM, 100 pM, 1000 pM, and 10000 pM) were added into the wells and co-incubated for 2 h at 37 ℃ (and 50 μL of 0.1 M PBS was used as control). Blocking buffer (200 μL) was added and after three washes (washing buffer: phosphate-buffered saline, 0.05% Tween-20) the plates were incubated for 2 h at 37 ℃. Then after repeat washing, the HRP-conjugated anti-S1 antibody (100 μL, 1 μg) was added into every well and incubated for 1 h at 37 ℃. After further washing steps and an incubation for 15 min at 37 ℃, a colorimetric signal developed due to the enzymatic reaction between HRP and the chromogenic substrate, TMB. Finally, 50 μL of stop buffer was added to terminate the reaction, and absorbance readings at 450 nm were acquired using a microplate reader (BioTek). For rac-NPs, the concentration is set to 0.1 nM, 1 nM, 10 nM, 100 nM, 1000 nM, 10000 nM, and 100000 nM.

S protein detection: S was diluted to 0.3 μg/mL in CBS (0.1 M, pH 9.7) and 100 μL was added into a 96-well plate and then different concentrations of NPs (50 μL of 0.01 pM, 0.1 pM, 1 pM, 10 pM, 100 pM, 1000 pM, and 10000 pM) were added into the wells and co-incubated for 2 h at 37 ℃ (and 50 μL of 0.1M PBS was used as control). Blocking buffer (200 μL) was added and after three washes (washing buffer: phosphate-buffered saline, 0.05% Tween-20) the plate was incubated for

2 h at 37 ℃. After repeat washing steps, the HRP-conjugated anti-S antibody (100 μL, 1 μg) was added into every well and incubated for 1 h at 37 ℃. After further washing a colorimetric signal was developed based on the enzymatic reaction between HRP and a chromogenic substrate, TMB, which were incubated for 15 min at 37 ℃. Finally, 50 μL of stop buffer was added to terminate the reaction, and absorbance readings at 450 nm were acquired using a microplate reader (BioTek). For rac-NPs, the concentration is set to 0.1 nM, 1 nM, 10 nM, 100 nM, 1000 nM, 10000 nM, and 100000 nM.

Determination of the binding between SARS-CoV-2 mimics and ACE2 using ELISA
ACE2 was diluted to 0.3 μg/mL in CBS (0.1 M, pH 9.7) and 100 μL was added into a 96-well plate for 2 h at 37 °C, and this was followed by blocking with blocking buffer (200 μL) and incubation for 2 h at 37 ℃ and three washes. Then the plate was subjected to several treatments.
Treatment 1: the RBD (100 μL, 0.3 μg/mL) was first incubated with different concentrations of L-NPs (50 μL of 0.01 pM, 0.1 pM, 1 pM, 10 pM, 100 pM, 1000 pM, and 10000 pM) for 2 h and then the pre-treated RBD was added into the ELISA plate coated with ACE2 for 2 h at 37 ℃. HRP-conjugated anti-RBD antibody was then added to the plate for 1 h at 37 ℃ and unbound HRP-conjugated anti-RBD antibody was washed away, and a colorimetric signal was developed based on the enzymatic reaction between HRP with the chromogenic substrate, TMB, after incubation for 15 min at 37 ℃. Finally, 50 μL of stop buffer was added to terminate the reaction, and the absorbance read at 450 nm using a microplate reader (BioTek).
Treatment 2: the RBD (100 μL, 0.3 μg/mL) was mixed with different concentrations of L-NPs (50 μL of 0.01 pM, 0.1 pM, 1 pM, 10 pM, 100 pM, 1000 pM, and 10000 pM) and immediately added into the ELISA plate coated with ACE2 for 2 h at 37 ℃. HRP-conjugated anti-RBD antibody was added to the plate for 1 h at 37 ℃. Then unbound HRP-conjugated anti-RBD antibody was washed, and a colorimetric signal was developed based on the enzymatic reaction of HRP with a chromogenic substrate, TMB, and incubated for 15 min at 37 ℃. Finally, 50 μL stop buffer was added to terminate the reaction, and absorbance readings at 450 nm were acquired using a microplate reader (BioTek).
Treatment 3: the RBD (100 μL, 0.3 μg/mL) was first added into the ELISA plate coated with ACE2 for 1 h at 37℃. Then different concentrations of L-NPs (50 μL of 0.01 pM, 0.1 pM, 1 pM, 10 pM, 100 pM, 1000 pM, and 10000 pM) were added into ELISA plate for another 1 h at 37 ℃. HRP-conjugated anti-RBD antibody was then added to the plate for 1 h at 37 ℃. Unbound HRP-conjugated anti-RBD antibody was washed away, and a colorimetric signal was developed based on the enzymatic reaction between HRP with a chromogenic substrate, TMB, which had been incubated for 15 min at 37 ℃. Finally, 50 μL of stop buffer was added to terminate the reaction, and the absorbance reading at 450 nm were acquired using a microplate reader (BioTek). Assessment of binding between the other two SARS-CoV-2 mimics and ACE2 were conducted using a similar procedure but HRP-labeled anti-S1 antibody and anti-S antibody were used instead.

CD and UV measurements
After the reactions were complete, the samples were directly characterized using a quartz cuvette. The temperature was maintained at 25 °C for all measurements, and the scanning range was 300 to 1000 nm for the chiral NPs with a scan speed of 0.2 nm/s and 190 to 260 nm for organic matter and a scan speed of 0.005 nm/s under high-purity nitrogen. At the same time, the CD spectra of the air and solvents were obtained and used as the baseline. It should be noted that when using CD spectroscopy to monitor the interaction of chiral NPs inhibiting the interactions between viral surface protein (RBD/S1/S) and ACE2, we first separated the supernatant from the precipitate by centrifugation (7000 rpm, 10 min), and then tested the CD signal of the supernatant as the chiral NPs form SPs with the viral surface proteins.

FT-IR measurements
The samples were prepared using the potassium bromide tablet method (wavelength range from 400 to 4000 cm^{-1}) and the analysis was performed with a Nicolet IS10 FT–IR spectrometer (Thermo fisher, USA).

ITC measurements
The NPs were purified by centrifugation (8000 rpm, 10 min) and re-suspended in PBS (10 mM, pH 7.4) at a final concentration of 10 nM of NPs. RBD/S1/S were injected into the sample cell at a concentration of 1.5 µg/mL with an injection volume of 50 µL, and 2 µL per injection (25 injections in total) at an injection interval of 300 s. The equilibrium time before the first injection was 600 s at 25 °C with a stirring rate maintained at 300 rpm during measurements. The original NanoAnalyze software (TA Instruments, USA) was used to analyze the data and to model the system with constant blank and independent models.

In vitro experiments
Cell viability assessment: To evaluate the viability of BEAS-2B cells treated with different conditions, we first tested the biocompatibility of the chiral NPs. BEAS-2B cells were seeded into 96-well plates at a density of 2×105 cells per well and cultured in DMEM supplemented with 10% fetal bovine serum, 50 units/mL penicillin, and 50 ug/mL streptomycin at 37 °C in a humidified incubator ventilated with 5% CO2. After 24 h, different concentrations of chiral NPs (0.05 nM, 0.2 nM, 1 nM, 20 nM, 100 nM, and 500 nM) were added into the 96-well plates and cultured for a further 12 h with PBS used as a control. CCK-8 reagent was added into the abovementioned 96-wells plate at a volume of 20 µL per well and incubated for 1 h. After this, the cells were washed with DPBS three times and a Multiskan™ MK3 microplate reader (Thermo Fisher Scientific Inc) was used to determine cell viability.

Optimized time for pseudovirus infections of BEAS-2B cells overexpressing hACE2
BEAS-2B cells (1×106) overexpressing ACE2 were seeded into 35 mm Petri dishes and cultured in DMEM supplemented with 10% fetal bovine serum, 50 units/mL penicillin, and 50 ug/mL streptomycin for 24 h. Next the SARS-CoV-2 pseudovirus (10 µL, 1.0 × 107 TU/mL) was added into the cells for various times (1, 2, 4, and 8 h). and the cells were washed with DPBS three times and fixed with 4% paraformaldehyde. After washing and fixation, immunofluorescence staining was performed with the anti-ACE2 antibody for 30 min followed by DAPI staining for 30 min. Finally, the cells were washed with DPBS three times and prepared for confocal microscopy and imaging.

Optimized time for the chiral NPs to enter BEAS-2B cells
BEAS-2B cells (1 × 106) overexpressing ACE2 were seeded into 35 mm petri dishes and cultured in DMEM supplemented with 10% fetal bovine serum, 50 units/mL penicillin, and 50 ug/mL streptomycin for 24 h. The chiral NPs (2 pM) were then added into the cells and cultured for various times (0, 6, 12, 24, and 48 h). At different time points, the cells were washed with DPBS three times and then fixed with 4% paraformaldehyde. After washing with DPBS for a further three times they were prepared for confocal microscopy imaging.

Inhibition SARS-CoV-2 pseudovirus infections by chiral NPs
Treatment 1 (Confocal Microscopy). The SARS-CoV-2 pseudovirus (10 µL of 1.0 × 107 TU/mL) was firstly incubated with different concentrations of L-NPs or D-NPs (0 pM, 2 pM, and 5 pM) for 2 h and then the pre-treated pseudovirus was added into BEAS-2B cells (2 × 105) cultured in DMEM supplemented with 10% fetal bovine serum, 50 units/mL penicillin, and 50 ug/mL streptomycin overexpressing ACE2 for 24 h. The cells were then washed with DPBS three time and then fixed with 4% paraformaldehyde solution for 30 min. Next the cells were washed with DPBS once and immunofluorescence staining was performed with the anti-ACE2 antibody for 30 min. Finally, the cells were washed with DPBS three times and prepared for confocal microscopy imaging.
Treatment 1 (Flow Cytometry). This was similar to the procedure used for confocal microcopy but with an increased number of concentrations (0.01 pM, 0.1 pM, 1 pM, 10 pM, 100 pM, 1000 pM, and 10000 pM).
Treatment 2 (Confocal Microcopy). SARS-CoV-2 pseudovirus (10 µL at 1.0 × 107 TU/mL) incubated with different concentrations of L-NPs or D-NPs (0 pM, 2 pM, and 5 pM) was added into BEAS-2B cells (2 × 105), and immediately cultured in DMEM supplemented with 10% fetal bovine serum, 50 units/mL penicillin, and 50 ug/mL streptomycin overexpressing ACE2 for 24 h. Then the cells were washed with DPBS three times and fixed with 4% paraformaldehyde solution for 30 min. After this, the fixed cells were washed with DPBS once and immunofluorescence staining was

performed with the anti-ACE2 antibody for 30 min. Finally, the cells were washed with DPBS three times and prepared for confocal microcopy imaging.

Treatment 2 (Flow Cytometry). This procedure was similar to that used for confocal microcopy except that an increased number of concentrations were used (0.01 pM, 0.1 pM, 1 pM, 10 pM, 100 pM, 1000 pM, and 10000 pM).

Treatment 3 (Confocal Microscopy). The SARS-CoV-2 pseudovirus (10 μL of 1.0 × 107 TU/mL) was firstly added into BEAS-2B cells (2 × 105) in DMEM supplemented with 10% fetal bovine serum, 50 units/mL penicillin, and 50 ug/mL streptomycin overexpressing ACE2 for 1 h. Next, different concentrations of L-NPs or D-NPs (0 pM, 2 pM, and 5 pM) were added into the infected BEAS-2B cells for 24 h. After this the cells were washed with DPBS three times and then fixed with 4% paraformaldehyde solution for 30 min. Then the fixed cells were washed with DPBS once and immunofluorescence staining was performed with the anti-ACE2 antibody for 30 min. Finally, the cells were washed with DPBS three times and prepared confocal microscopy imaging.

Treatment 3 (Flow Cytometry). This procedure was similar to the confocal procedure except more concentrations were used (0.01 pM, 0.1 pM, 1 pM, 10 pM, 100 pM, 1000 pM, and 10000 pM). For rac-NPs, the concentration is set to 0.1 nM, 1 nM, 10 nM, 100 nM, 1000 nM, 10000 nM, and 100000 nM.

Inhibition of the infection of SARS-CoV-2 mimics by chiral NPs

For RBD: HEK293 cells overexpressing RBD were cultured in 6-well plates (5.0 × 105) with DMEM supplemented with 10% fetal bovine serum, 50 units/mL penicillin, and 50 ug/mL streptomycin at 37 °C. Different concentrations of L-NPs (0.01 pM, 0.1 pM, 1 pM, 10 pM, 100 pM, 1000 pM, and 10000 pM) were then added into the 6-well plates for 18 h after an initial 24 h to allow cell for attachment. Next the cells were collected via centrifugation (1200 rpm, 3 min) for flow cytometry and quantification using the FITC-labeled anti-RBD antibody (Signals collected from 1.0 × 105 cells).

For S1 protein: HEK293 cells overexpressing S1 protein were cultured in 6-well plates (5.0 × 105) in DMEM supplemented with 10% fetal bovine serum, 50 units/mL penicillin, and 50 ug/mL streptomycin at 37 °C. Then different concentrations of L-NPs (0.01 pM, 0.1 pM, 1 pM, 10 pM, 100 pM, 1000 pM, and 10000 pM) were added into the 6-well plates for 18 h after an initial 24 h to allow cell for attachment. After this the cells were collected by centrifugation (1200 rpm, 3 min) for quantification by flow cytometry using the FITC-labeled anti-S1 antibody (signals collected from 1.0 × 105 cells).

For S protein: HEK293 cells overexpressing S protein were cultured in 6-well plates (5.0 × 105) in DMEM supplemented with 10% fetal bovine serum, 50 units/mL penicillin, and 50 ug/mL streptomycin at 37 °C. Different concentrations of L-NPs (0.01 pM, 0.1 pM, 1 pM, 10 pM, 100 pM, 1000 pM, and 10000 pM) were then added into the 6-well plates for 18 h after an initial 24 h to allow cell for attachment. Next the cells were collected by centrifugation (1200 rpm, 3 min) for quantification by flow cytometry using the FITC-labeled anti-S antibody (signals collected from 1.0 × 105 cells). For rac-NPs, the concentration is set to 0.1 nM, 1 nM, 10 nM, 100 nM, 1000 nM, 10000 nM, and 100000 nM.

Comparison of inhibitory effects of chiral NPs and antibodies

ELISA: The SARS-CoV-2 pseudovirus (100 μL of 1.0 × 104 TU/mL) was seeded into 96-well plates for 2 h at 37 ℃. Then different concentrations of chiral NPs (50 μL at 0.01 pM, 0.1 pM, 1 pM, 10 pM, 100 pM, 1000 pM, and 10000 pM) were added into ELISA plate for another 1 h at 37 ℃. ACE2 was then added to the plate for 1 h at 37 ℃. Finally, HRP-labeled anti-ACE2 antibody was then added, and unbound antibody was washed away, and a colorimetric signal was developed based on the enzymatic reaction between HRP with a chromogenic substrate, TMB, which had been incubated for 15 min at 37 ℃. Finally, 50 μL of stop buffer was added to terminate the reaction, and the absorbance readings at 450 nm were acquired using a microplate reader (BioTek). Assessment of the inhibitory effects of rac-NPs and antibody (Biodragon, BF04204) were conducted using a similar procedure but with different concentrations (antibody: 0.001 nM, 0.01 nM, 0.1 nM, 1 nM, 10 nM, 100 nM, and 1000 nM; rac-NPs: 0.1 nM, 1 nM, 10 nM, 100 nM, 1000 nM, 10000 nM, and 100000 nM).

Flow Cytometry: The SARS-CoV-2 pseudovirus (10 μL of 1.0 × 107 TU/mL) was firstly incubated with different concentrations of chiral NPs, rac-NPs and antibody for 2 h and then the pre-treated pseudovirus was added into BEAS-2B cells (2 × 105) overexpressing ACE2 for 48 h. The cells were then washed with DPBS three times and then fixed with 4% paraformaldehyde solution for 30 min. Next the cells were washed with DPBS once and immunofluorescence staining was performed with the anti-ACE2 antibody for 30 min. Finally, the cells were washed with DPBS three times and prepared for flow cytometry.

Authentic viral inhibition assays

Authentic viral neutralization assays were conducted in BSL3 of Kunming Institute of Zoology, CAS. Since SARS-CoV-2 infection causes a significantly decreased activity in Vero E6 cell, we calculated the viral inhibition rate by testing host cell activity using CCK-8 method. Detailed protocol followed as: First, Vero E6 cells (2×105 cells/well) were inoculated to a 96-well plate and cultured overnight at 37 °C with 5% CO2. The different concentrations of L-NPs (100 μL, 30 pM, 10 pM, 3.33 pM, 1.11 pM, 0.37 pM, and 0.123 pM), resuspended in EMEM (0% FBS) with three repeated wells were mixed with SARS-COV-2 (100 μL, resuspended in EMEM with 0% FBS) that multiplicity of infection **(MOI)** was 0.1, and incubate at 37 °C, 5% CO2 for 1h. A positive control without L-NPs (virus group) and a negative control without SARS-COV-2 (chiral NPs group) are set at the same time. The above-mentioned mixture was added into 96-wells plate (200 μL/well) at 37°C, 5% CO2 and incubated for three days, after the culture medium was removed from cell culture plate. The CCK-8 kit was used to detect the cell viability, and the Bio-Tek microplate detector was used to determine the OD value. The measurement wavelength was 450 nm, and the reference wavelength was 630 nm. Calculate the inhibition rate of the L-NPs on virus replication and determine the antiviral effect of L-NPs.

Animal experiments

Male BALB/c mice (aged 7 weeks) purchased from Beijing Vital River Laboratory Animal Technology Co; Ltd. hACE2-expressing TG male mice (aged 6 weeks) purchased from Zhishan (Beijing) Healthcare Research Institute Ltd. All mice used in this study are in good health and were housed under SPF conditions with a 12-hour light and dark cycle. All animal handling and procedures were performed according to approved guidelines and in accordance with the Institutional Animal Care and Use Committee of Nanjing Agricultural University, Nanjing, China, under the permit number NJAU. No20220821013.

Toxicity studies in mice

Male BALB/c mice were treated with PBS and *L*-NPs (5 nM per animal) via inhalation. After 7 days and sacrifice, blood (blood tests) and major organs (H&E staining) were collected for toxicity evaluation.

Biodistribution of the NPs in mice

L-NPs (5 nM per body) were delivered to BALB/c mice via inhalation and then the mice were sacrificed at 0, 24, 48, and 72 h. All major organs were collected for further analysis of the chiral NPs post-inhalation.

LC-MS measurements

Liquid chromatography-mass spectrometry was performed using an LC-MS system (Waters). Samples of 0.3 μL were aspirated using an auto-sampler and bound to a C18 capture column (5 μm, 5 × 0.3 mm), and then eluted into an analytical column (2.1 × 150 mm, 1.7 μm) for separation and a 12-min analytical gradient (0 min of 2% B, 5 min of 10% B, 8 min of 30% B, 40% B for 10 min, and 12 min of 80% B, and A represents the stationary phase, and B represents the mobile phase) was established. The flow rate of the liquid phase was set to 0.3 mL/min. In MS ESI+ mode analysis, each scan cycle included a full MS scan (m/z range 20–1500, ion accumulation time 250 ms), and 40 subsequent MS/MS scans (m/z range 100–1500, ion accumulation time 50 ms). The MS/MS acquisition conditions were set to a precursor ion signal greater than 120 cps and a charge number of +1 to +10. The ion repeat acquisition exclusion time was set to 18 s.

Computer simulations

Pen-NP parameterization

The molecular dynamics simulations of the interactions between NPs functionalized with penicillamine and the RBD protein required the parameterization of the force field, which lacks proper parameters for the ceramic, using quantum chemistry calculations, as described below.

The structure of the model CuS nanoparticle was drawn using the VESTA 3.4.4 software (22), by the replication of the covellite (CuS) unit cell (Crystallography Open Database, ID 9000523) (23) into a supercell with 25×23×13 unit cells in the a, b, and c directions, respectively. The NP was cut from the supercell using the crystallographic planes observed experimentally, (101), (102), (103), (105), (111), (112), (113), (115), and (006), yielding a bare NP with ca. 4 nm in length and a stoichiometry $Cu_{597}S_{711}$.

The model NP had 78 S atoms with low coordination numbers, which were replaced by the terminal S atom of the Pen ligands, yielding a surface density of 1.0 molecule per nm^2, close to the value of 1.2 molecules per nm^2 obtained experimentally. The experimental results also indicated that the NP presented a net negative charge, which was estimated to be q = –14 e in the model system (this charge yielded the lowest energy for the bare NP with the low coordination S atoms saturated with a hydrogen atom, computing the energies with the GFN1-xTB semiempirical Hamiltonian (24) as implemented in the xTB 6.4.0 software for charges between 0 and -100 e, in 2 e intervals).

Two model systems were built, one named L-NP bearing 78 L-Pen ligands and the other named rac-NP with 39 L-Pen and 39 D-Pen added alternately (the D-NP was not drawn since it is by definition the mirror image of the L-NP and as such it does not require a specific parameterization). The ligands were added with random orientations and were relaxed keeping the Cu and S atoms frozen at their crystallographic positions, using a force field with a set of generic partial charges. After the geometry optimization, the structure was subjected to an MD simulation with a temperature ramp from 3000 K to 0 K in 1 ns. The structures with the frozen Cu and S core were then fully optimized at the DFT level using the GFN1-xTB semiempirical Hamiltonian with the ALPB implicit solvation model for water and an electronic temperature of 1000 K. After 500 cycles of energy minimization, the structures were further relaxed by 100 fs of molecular dynamics at T = 473 K (the same temperature of synthesis) at the same level of theory, with an integration time step of 0.5 fs. Finally, another geometry optimization round with 400 cycles was performed, resulting in an energy minimization with an energy change below 1×10^{-4} Eh and a final gradient below 2×10^{-2} Eh/bohr. The final set of interatomic distances were used to construct a list of all Cu and S atoms closer than 0.35 nm, which were considered as bonded and were kept at the geometry obtained from the DFT calculations by means of a force constant of 3×10^5 kJ mol-1 nm^{-2}. This setup enforces that all Cu and S atoms of the NP remain vibrating around the minimum obtained quantum chemically, similarly to another paper published previously (25), ensuring that the chiral distortions imparted by the ligands remain stable in the subsequent MD simulations. The Lennard-Jones parameters for S atoms (both in the disulphide and thiolate atom types) were taken from the July/2021 update of the Charmm36 force field (26), whereas those for Cu atoms were taken from the literature (27). Bonded parameters and Lennard-Jones parameters for ligands were taken from Charmm36 force field using the SwissParam server (28). The atomic partial charges for all atoms comprising the NPs decorated with the ligands were described using CM5 population calculated with the GFN1-xTB semiempirical Hamiltonian, averaging the charges for the ligands but keeping all Cu and S atoms with the charges from the DFT calculation.

Molecular dynamics (MD) simulations

Three independent MD simulations were carried out for the L-NP, D-NP, and rac-NP interacting with one unit of the RBD protein, as follows: i) the RBD protein (PDB code 6m0j) (29) was centered in a periodic cubic box with 13 nm of edge length; ii) one NP (either D, L, or rac) was placed in contact with the RBD portion that interacts with the human ACE-2 in the original PDB file (residues 449-458 and 476-505 in **Figure S36**); iii) atoms from all Pen ligands and also from RBD structure were allowed to relax (in vacuum) to eliminate bad initial contacts and also to maximize favorable NP-Pen-RBD interactions; iv) a total of 5 x 10^4 water molecules were added along with 12 sodium ions to neutralize the system charge. Energy minimization steps were carried out to remove repulsive contacts and the resulting structures were equilibrated for 2 ns with a position restraint potential for all copper atoms of the NPs at the NpT ensemble (T = 300 K, p = 1 bar) using Berendsen weak-coupling algorithms to control both temperature and pressure (30), followed by 5 ns of MD simulation without position restraints. Data collection runs corresponded to 1000 ns at the NpT ensemble (T = 300 K, p = 1 bar) using Nosè-Hoover thermostat (31, 32) and Parrinello-Rahman barostat (33). All MD simulations were carried out using a cutoff of 1.2 nm for both Lennard-Jones and Coulomb potentials. For the former, a force-switch was applied from 1.0 nm to 1.2 nm, while the truncation errors for latter were corrected by means of the Particle-Mesh Ewald algorithm (34, 35). The LINCS algorithm (36) was used to keep all bonds containing hydrogen atoms rigid along their equilibrium geometry along the dynamics, allowing the use of a time step of 2 fs. The energy minimization and molecular dynamics simulations/analysis were performed using the Gromacs 2021.2 package (37-39).

Machine Learning (ML) Predictions

PDB files were obtained from MD for NPs. The PDB files for proteins were obtained from RCSB following the general protocol and utilizing the database described in a published paper (40). The solvent accessible surface and normal vectors were extracted using PyMOL. The ML model based on Pointnet++ was trained using protein-protein interactions. Interacting residues were defined as any residue in one protein that had non-hydrogen atoms within 4 Å of a non-hydrogen atom in the other protein. These interacting residues were mapped onto the surface based on the surface points nearest to these residues.

Statistical Methods and Reproducibility

All the experimental data are presented as mean ± SD (n = 3) with the error bar representing the standard deviation. Significant differences were assessed by Student's t-test, and the criterion was *P < 0.05, **P < 0.01, ***P < 0.001, respectively. All the micrographs obtained from experiments showed in main text and supplementary information were the representative experimental results from more than three independent experiments (at least n=3). And the results of these independent experiments remain highly consistent.

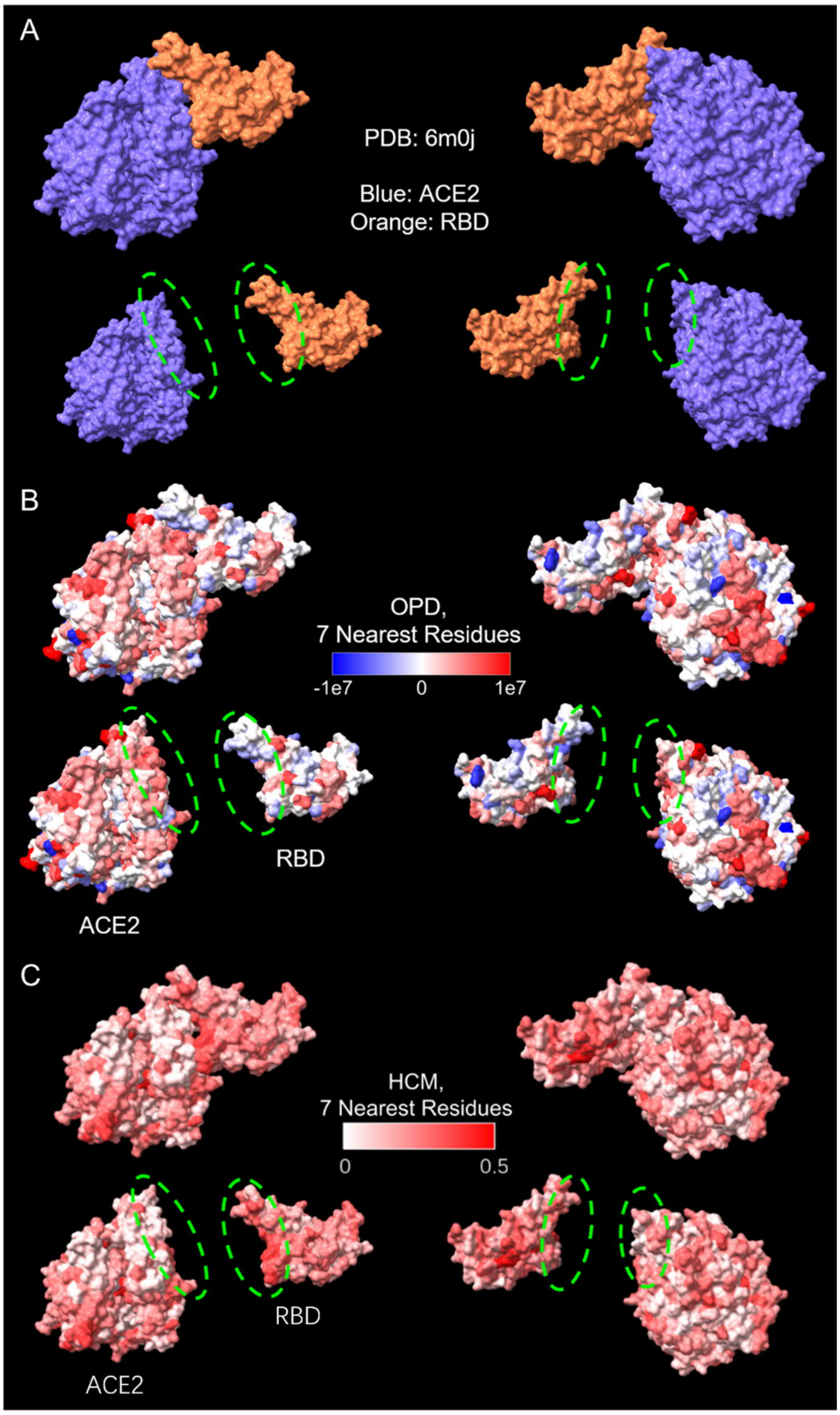

Fig. S1. (A) 3D representation of the protein complex between ACE2 and RBD at different views; **(B)** Color map for the Osipov-Pickup-Dunmur **(OPD)** chirality measures for ACE2 and RBD. The circled area is the interface between ACE2 and RBD. **(C)** Color map for the Hausdorff Chirality Measure (HCM, aka Hausdorff distance) for ACE2 and RBD. The circled area is the interface between ACE2 and RBD.

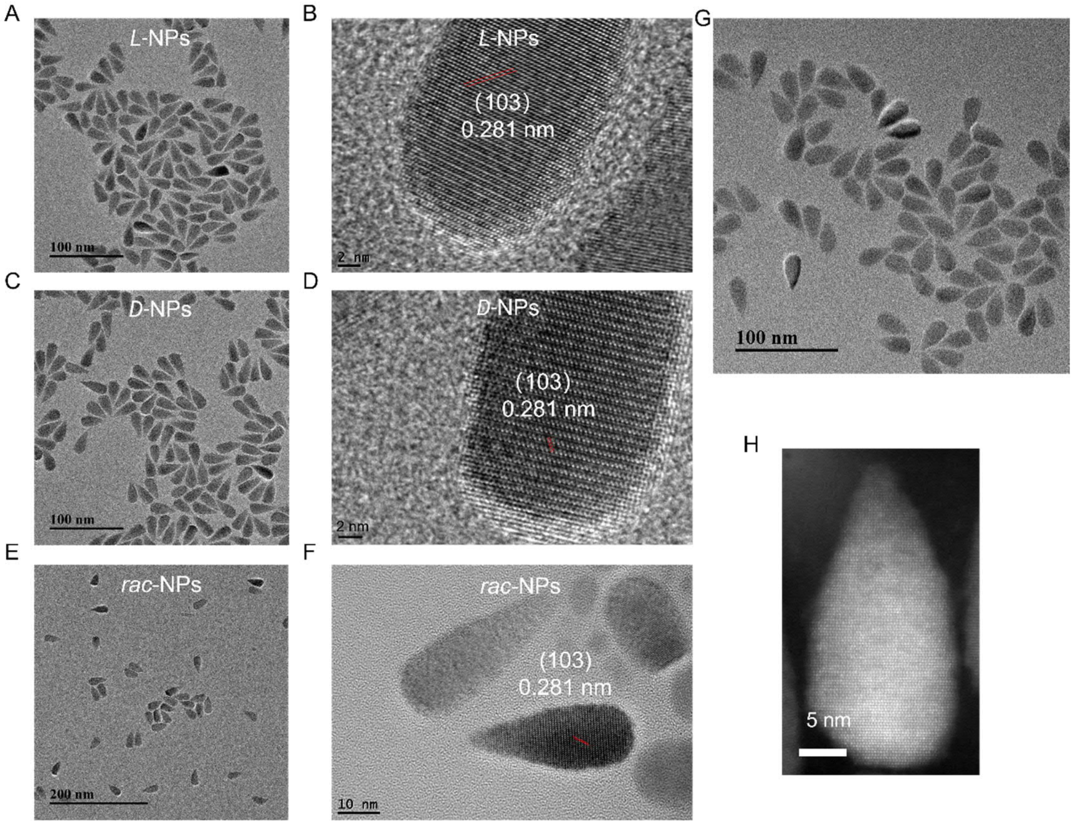

Fig. S2. The TEM **(A),** HR-TEM **(B)** image of L-NPs. The TEM **(C),** HR-TEM **(D)** image of D-NPs. TEM **(E)** and HR-TEM **(F)** images of rac-Pen-modified NPs. The TEM image **(G)** and ADF-STEM **(H)** of no chiral ligands modified NPs.

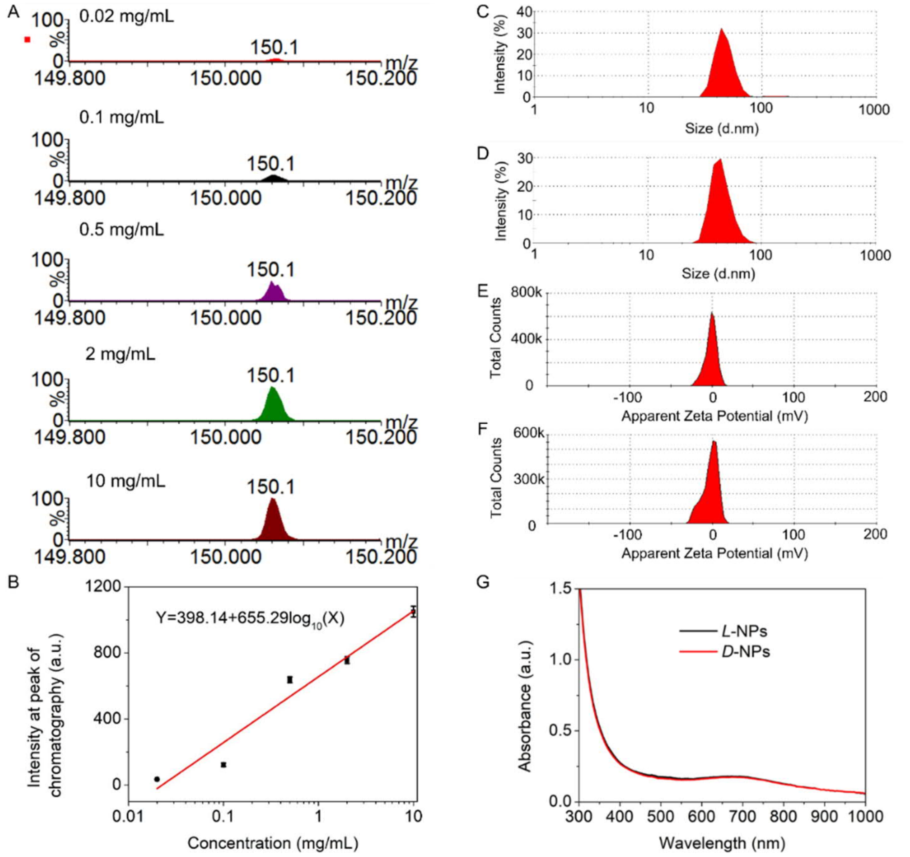

Fig. S3. (A) The LC-MS data of the number of ligands on the surface of NPs. **(B)** The standard curve established by different concentrations of L-Pen and corresponding mass spectrum peak integrated area. Data are presented as mean ± s.d. (n = 3). The error bars in **Fig. S3B** calculated from standard deviation from three independent experiments. The size distribution of L-NPs **(C)** and D-NPs **(D)**. The zeta potential of L-NPs **(E)** and D- NPs **(F)**. (The hydrate particle size of chiral NPs was ≈ 40 nm and zeta potential about -8 mV). The absorbance **(G)** spectra of chiral NPs.

Comments: The number of ligands of single chiral NPs was determined by using LC-MS. We first established a standard curve of Pen ligands (**Figs. S3A-B**). Then, 1 mg of L-NPs (Mt = 1 ×10-3 g) were first treated with 1 M HCl (1 mL) for 1 hour to degrade NPs to free the Pen ligands before LC-MS testing. According to the standard curve (**Fig. S3B**), we thus obtained the concentration (0.0214 mg/mL) and mass (0.0214 mg) of Pen ligands in chiral NPs. The number of moles (n) of Pen ligands (n_Pen) present in the analyte is n=m/M , where m is the mass of the Pen ligands, and M is the relative molecular mass of Pen ligands (149.21). Thus, the n of Pen (n_Pen) = m/M= (0.214×10^(-4))/149.21=1.434 × 10-7 mol, and the total number of Pen molecules (NPen) = n_Pen×NA= 1.434 × 10-7 × NA, where NA is the Avogadro's constant equal to 6.022×1023. Thus, the NPen= n_Pen×NA= 1.434 × 10-7 × 6.022 × 1023 = 8.637 × 1016 for the entire sample. The number of moles of NPs in the 1mL sample is n_NPs=(M_t ×2.77×10^17)/NA = Mt × 4.60 ×10-7 mol, where Mt is 1 ×10-3 g (see above). Thus, the number of chiral NPs per sample NNPs = n_NPs × NA = 4.60 ×10-10 × 6.022×1023 = 2.7 × 1014. One can calculate the number of ligands on the surface of a single NP as N_Pen/N_NP = 〖8.637×10〗^16/〖2.7×10〗^14 ≈320.

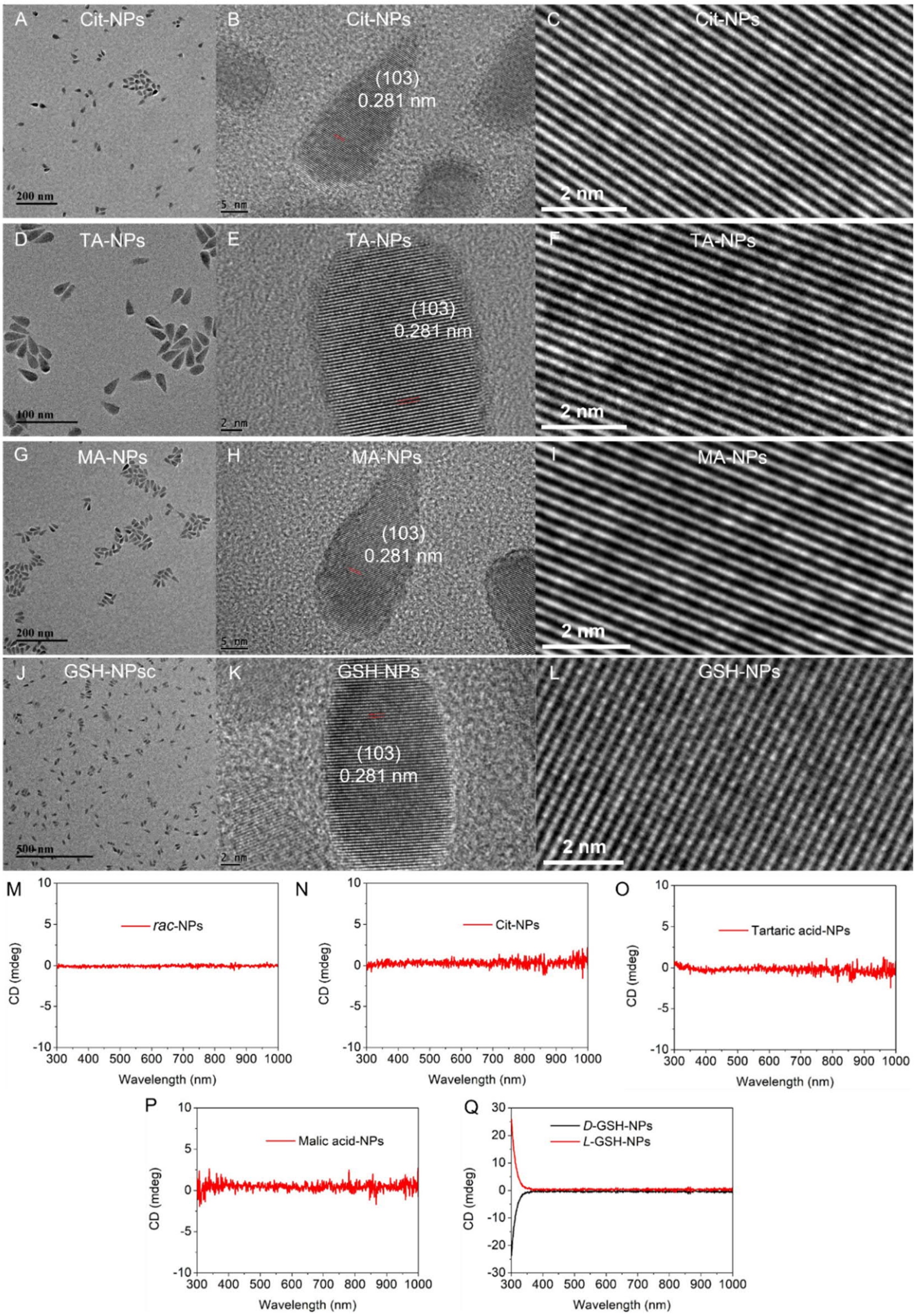

Fig. S4. The TEM **(A)** and HR-TEM **(B)** of Cit-NPs and magnified area **(C)** in B. The TEM **(D)** and HR-TEM **(E)** of TA-NPs and magnified area **(F)** in E. The TEM **(G)** and HR-TEM **(H)** of MA-NPs and magnified area **(I)** in H. The TEM **(J)** and HR-TEM **(K)** of GSH-NPs and magnified area **(L)** in K. The CD spectra of rac-NPs **(M)**, Cit-NPs **(N)**, TA-NPs **(O)**, MA-NPs **(P)** and GSH-NPs **(Q)**. Rac-NPs: racemic Pen modified the CuS NPs; Cit-NPs: citric acid modified the CuS NPs; TA-NPs: tartaric acid modified the CuS NPs; MA-NPs: malic acid modified the CuS NPs.

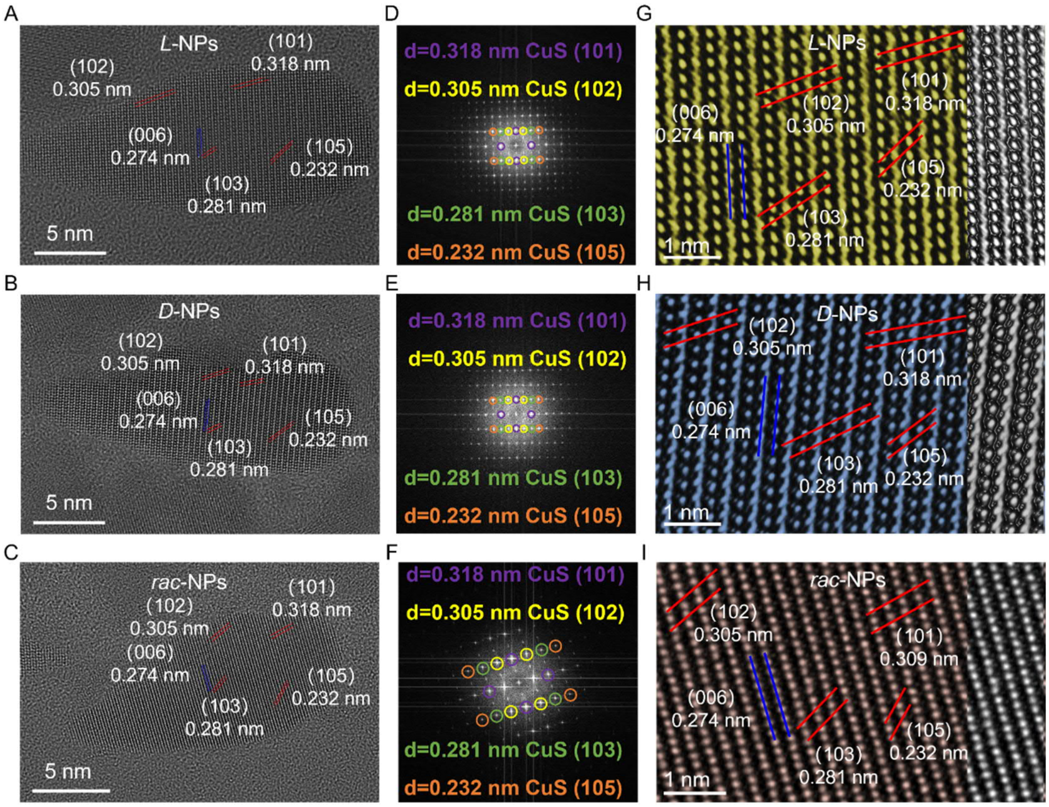

Fig. S5. The spherical aberration correction TEM image of L-NPs **(A)**, D-NPs **(B)** and rac-NPs **(C)**. Fast Fourier Transform diffraction analysis of spherical aberration correction TEM images of L-NPs **(D)**, D-NPs **(E)** and rac-NPs **(F)**. The HR-SAC-TEM image of L-NPs **(G)**, D-NPs **(H)** and rac-NPs **(I)**.

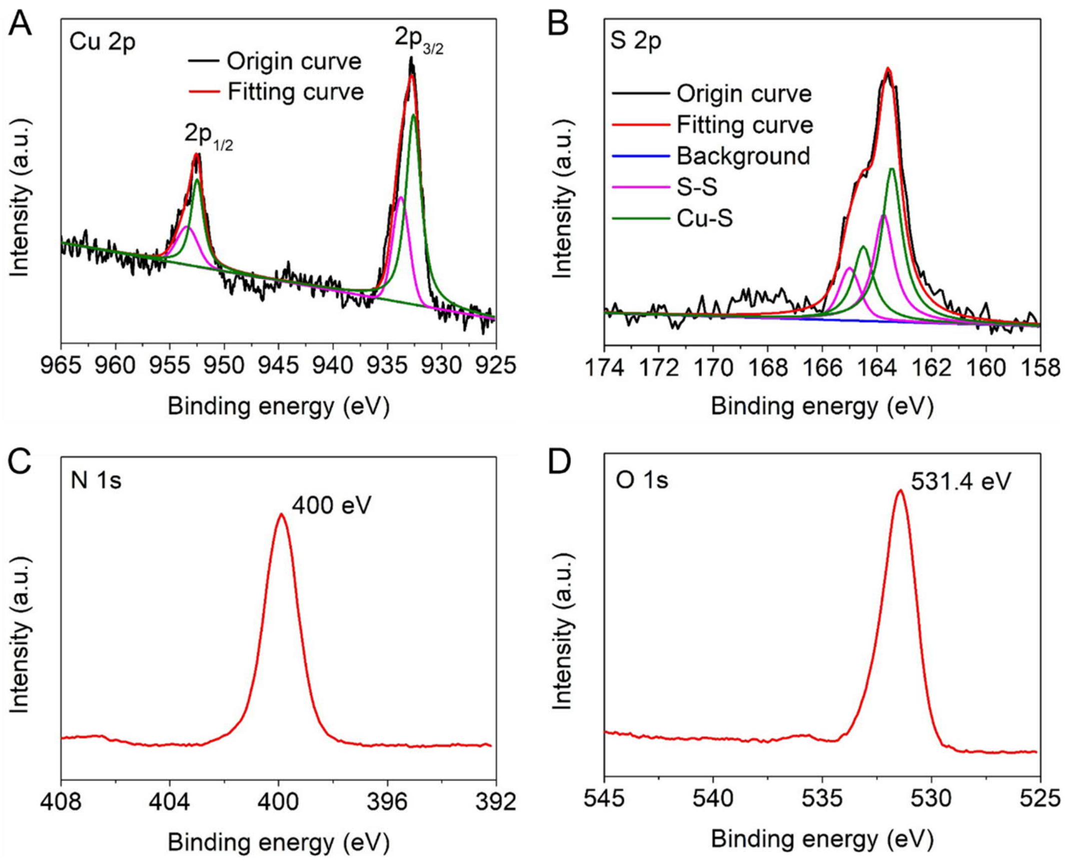

Fig. S6. The binding energy of Cu **(A)**, S **(B)**, N **(C)**, and O **(D)** of L-NPs.
Comments: The peaks of binding energy at 952.4 eV and 932.6 eV represents Cu+ in 2p1/2 and 2p3/2 of CuS (covellite). The peaks of binding energy at 953.4 eV and 933.8 eV represents oxidation of Cu+ to produce Cu2+ due to Pen binding the surface of CuS NPs by sulfhydryl. The peaks of binding energy at 163.3 and 163.9 eV of S represented Cu-S in 2p3/2, 164.4 and 165.1 eV of S represented S-S in 2p1/2, respectively in the XPS spectra.

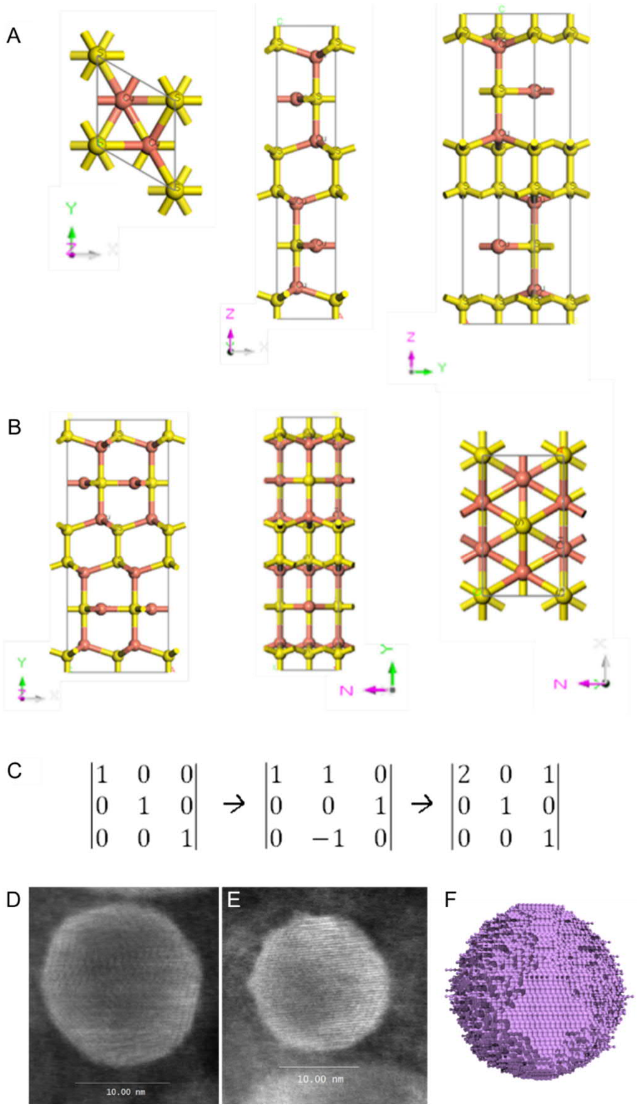

Fig. S7. Conversion from hexagonal to orthorhombic CuS lattice. **(A)** Three different projections of hexagonal CuS lattice with parameters a,b,c = 3.796 Å, 3.796 Å, 16.382 Å, α ,β ,ϒ = 90°, 90°, 120° are shown. **(B)** Orthorhombic lattice derived from hexagonal lattice is shown along three different projections with lattice parameters a,b,c = 6.5748 Å, 16.382 Å, 3.796 Å, α ,β ,ϒ = 90°, 90°, 90°. **(C)** The symmetry operations applied for the above conversion are listed. The conversion is done to orient the crystal such that the direction of propagation of electron beam is along z-direction and the simulated FFT matches the experimentally observed FFTs of the crystal. Top view of NPs (D-E) ADF-STEM of rac-NPs. **(F)** Projection of a reconstructed without ligands modified NP shown in **Fig. 1K**. ADF-STEM images show hexagonal bounding faces indicated the crystalline nature of the particle. The more pattern of fringes inside the particle indicates the presence of dislocations within the crystal.

Comment: NPs grew preferentially along the c-axis of a CuS unit cell and there were dislocations within the NPs before being modified with Pen. These dislocations were one-dimensional in nature and extended along the a-direction (**Figs. 1M**) suggested stacking faults that end as kink sites on the edge of the NP. Upon modified with Pen ligands, NPs showed significantly lower one-dimensional dislocation extending along a-direction (**Figs. 1N**).

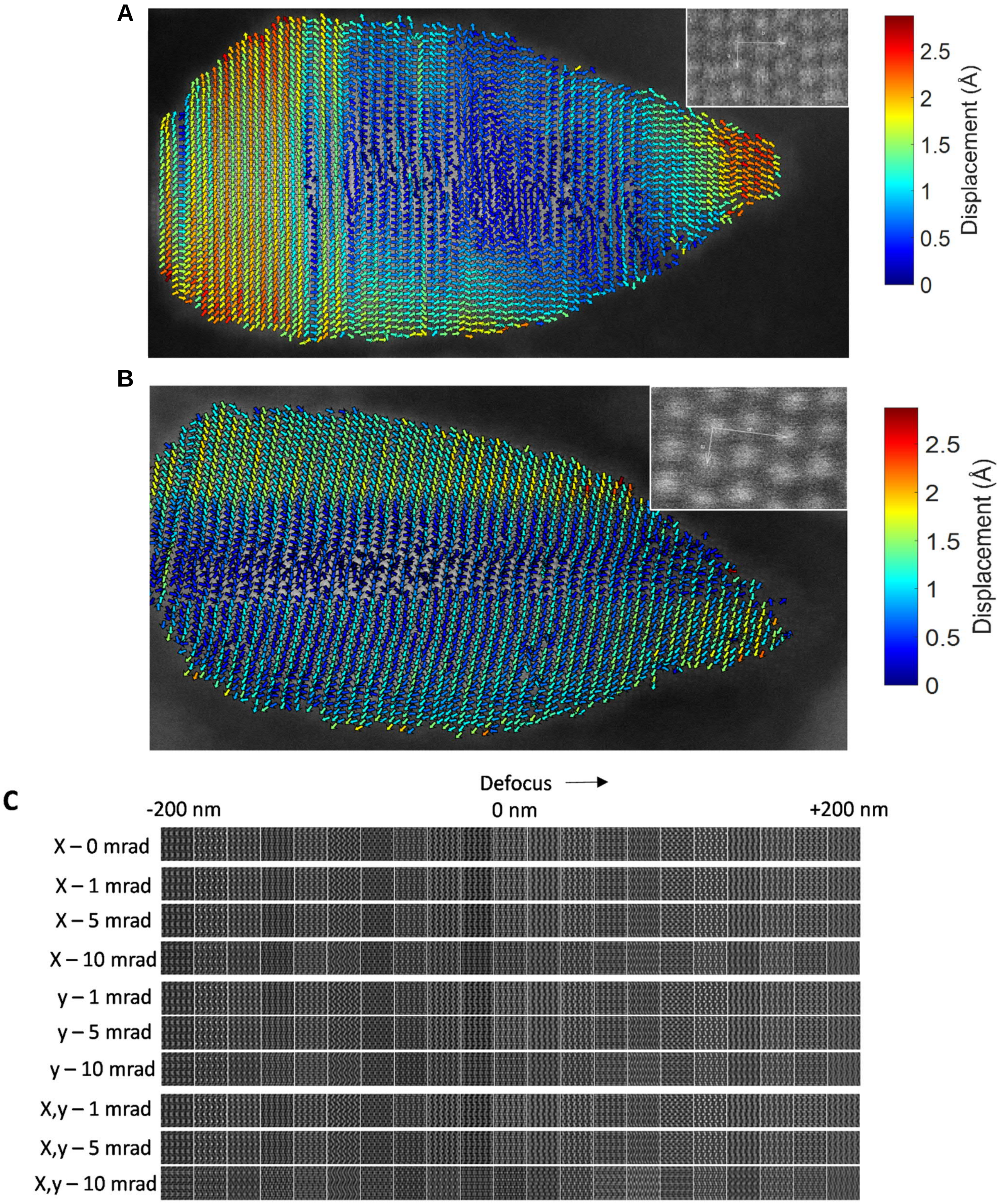
A
Displacement (Å)
0
0.5
1
1.5
2
2.5
B
Displacement (Å)
0
0.5
1
1.5
2
2.5
C
Defocus
-200 nm
0 nm
+200 nm
X – 0 mrad
X – 1 mrad
X – 5 mrad
X – 10 mrad
y – 1 mrad
y – 5 mrad
y – 10 mrad
X,y – 1 mrad
X,y – 5 mrad
X,y – 10 mrad

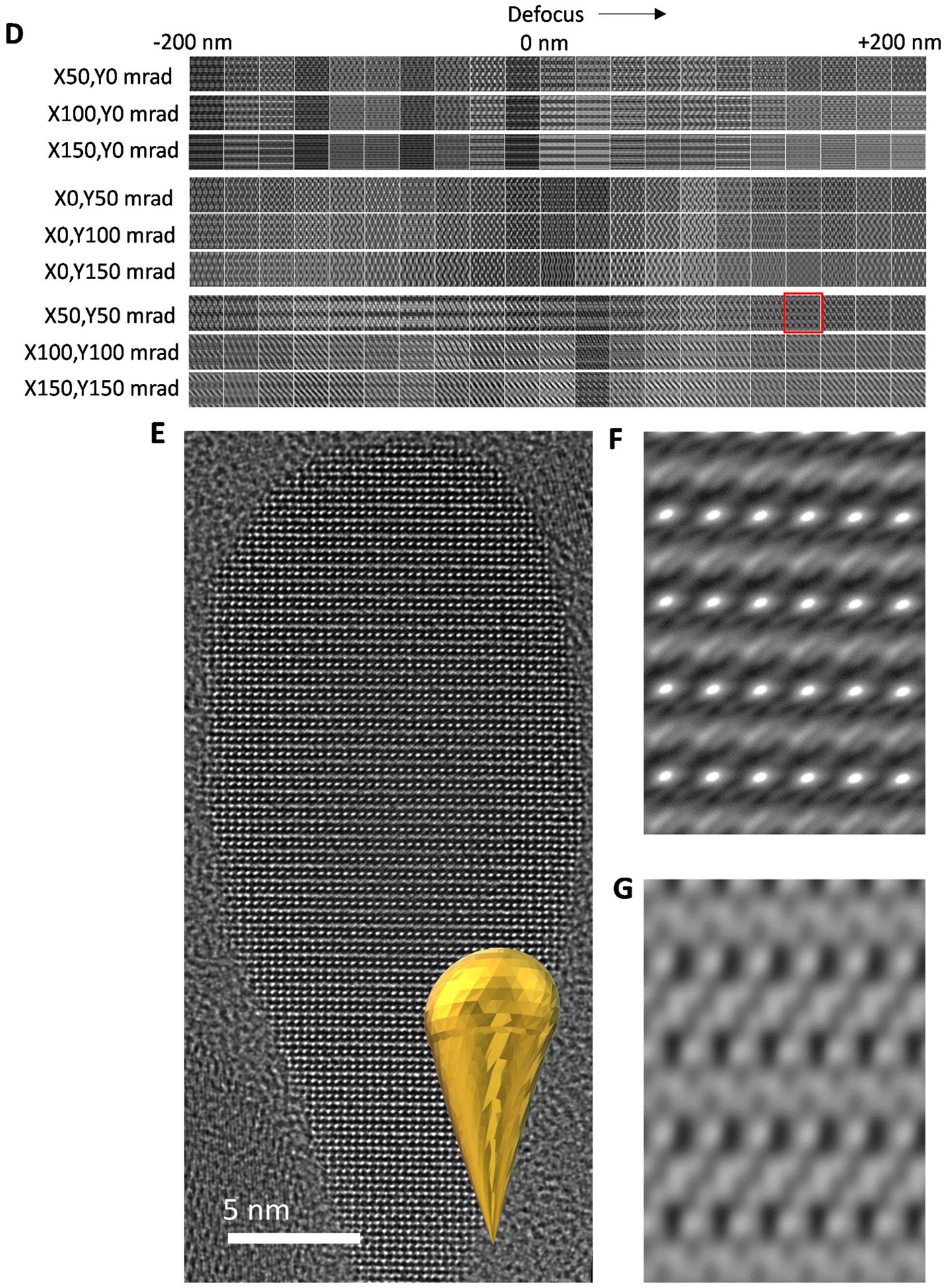
D
Defocus
-200 nm
0 nm
+200 nm
X50,Y0 mrad
X100,Y0 mrad
X150,Y0 mrad
X0,Y50 mrad
X0,Y100 mrad
X0,Y150 mrad
X50,Y50 mrad
X100,Y100 mrad
X150,Y150 mrad
E
5 nm
F
G

Fig. S8. Displacement map of atomic positions from a projected unit cell of a = 3.3 Å, b = 6.8 Å, α = 90° shown in inset and calculated from fast Fourier transform of images is shown for no chiral ligands modified NP **(A)** and L-Pen modified the NP **(B)**. The arrows at each atomic column indicate the direction in which the lattice is strained as compared to the projected unit cell and the colors indicate the magnitude of displacement. BF-TEM image of no ligands modified NPs **(C)** and L-Pen modified NPs **(D)** simulation of tilt-defocus maps. Orthorhombic lattice oriented along z-direction with a 150 Å × 150 Å × 100 Å thick crystal is simulated under BF-TEM imaging conditions with V = 200 keV, Cs3 = 2 mm, Objective aperture size = 10 mrad, and Astigmatism = 0 with a resolution of 0.07 Å/pixel. Thickness of 10 nm is estimated from experimental TEM images of NPs with the largest lateral dimension. Simulated image highlighted in red corresponds to the closest visual match with experimental motifs. (E-G) Comparison between simulated and experimental BF-TEM images. **(E)** BF-TEM of L-NPs showing the lattice fringes that are shifted when compared between the bulb and the tip of the NPs. **(F)** Small section from panel a is magnified and compared with **(G)**, simulated BF-TEM image marked by red box in **Fig. S8D**.

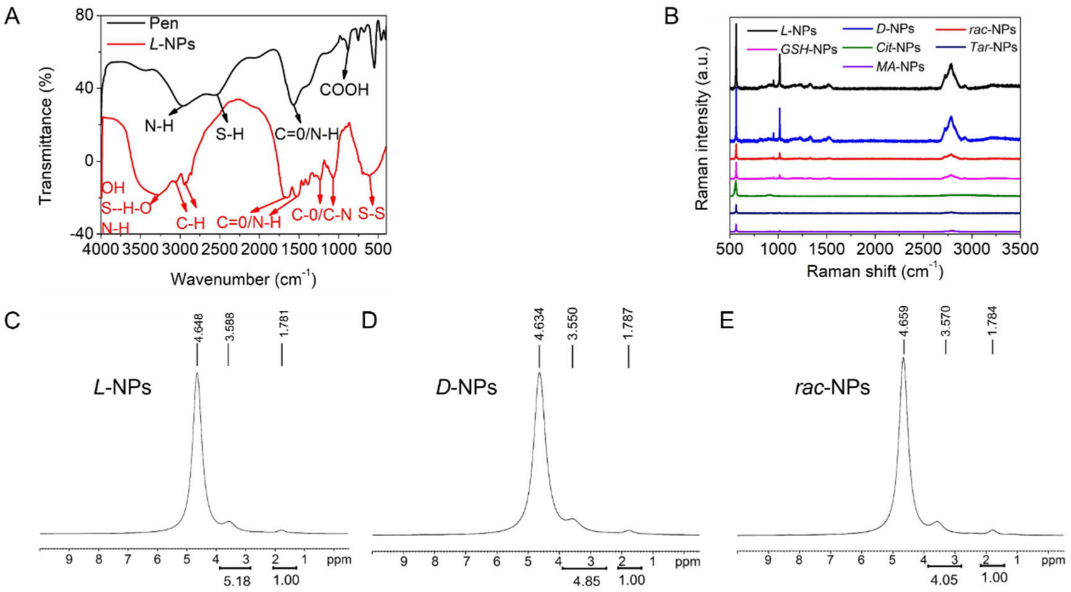

Fig. S9. (A) FT-IR spectra of chiral NPs. **(B)** The Raman spectra of different ligand modified NPs. 1H-NMR (700 MHz, Bruker AVANCE NEO) of L-NPs **(C)**, D-NPs **(D)** and rac-NPs **(E)** in deuteroxide.

Comments: 521 cm-1 represents Cu-S bond; 987 cm-1 represents COOH group; 1056 cm-1 represents C-O; 1367 cm-1 and 1601 cm-1 represent amide bond; 2800 cm-1 to 3300 cm-1 represents -C-H and hydrogen bond. The intensity of the peaks from sulfhydryl and carboxyl groups in Fourier-transform infrared (FT-IR) spectra was significantly reduced while the intensity of the peak from hydrogen bonds and hydroxyl groups increased in NPs compared to free Pen. This finding is consistent with the XPS data that binding energy of O in NPs shifted to the low energy area, due to the ionic bonds formed between Cu and the carboxyl group (**Fig. S9**). FTIR results showed that the Pen ligands bonded with NPs surface via strong S-Cu covalent bond and COOCu ionic bond and weak S--H-O hydrogen bonds and the surface of NPs coordinated with hydroxy (-OH) due to the terminal PH was at physiologically pH). 1H NMR spectra showed that the D-NPs and L-NPs have almost identical chemical shifts, but they were significantly different than those for rac-NPs (**Fig. S9**). Raman scattering peaks were observed when L-NPs and D-NPs were used compared with other ligands stabilized NPs (**Fig. S9**).

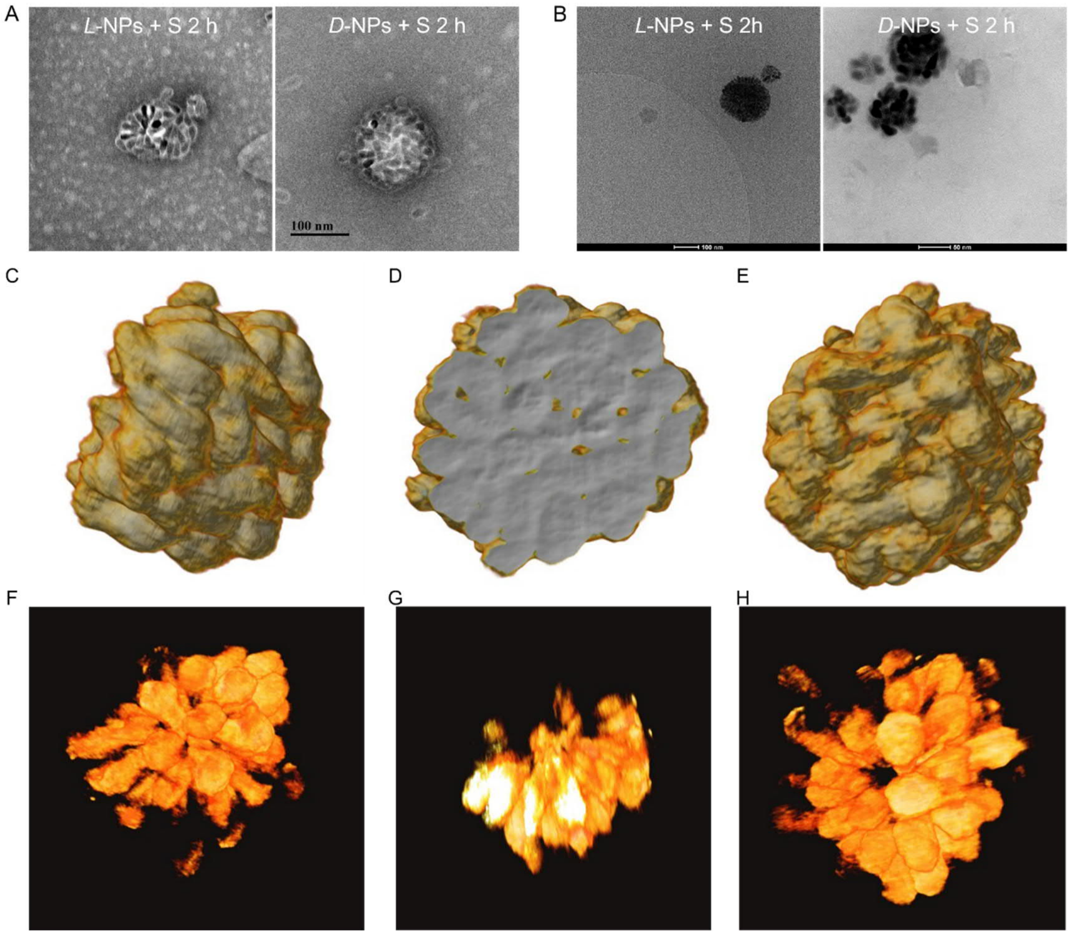

Fig. S10. (A) Native staining TEM of S incubated with L-NPs and D-NPs for 2 h. **(B)** Cryo-TEM images of S incubated with L-NPs and D-NPs for 2 h. TEM tomography images of SPs comprised D-NPs and S at main view **(C)**, cross section **(D)** and side view **(E)**. TEM tomography images of SPs comprised L-NPs and S at main view **(F)**, cross section **(G)** and side view **(H)**.

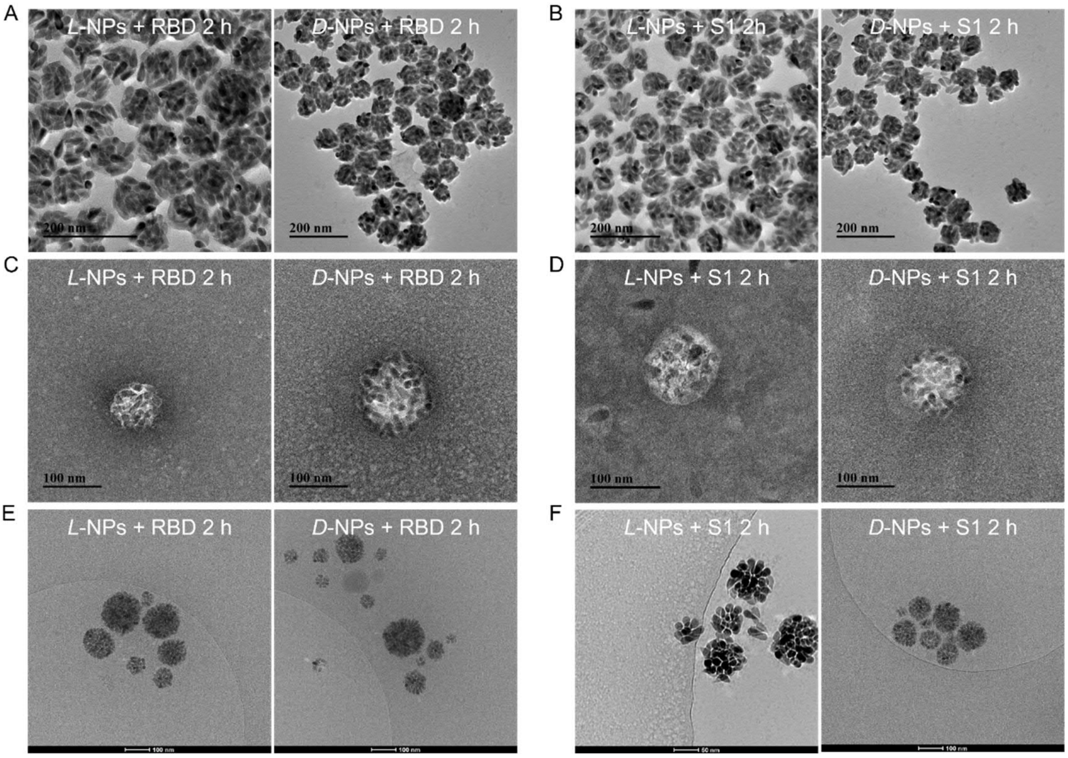

Fig. S11. TEM images of RBD **(A)** or S1 **(B)** incubated with L-NPs and D-NPs for 2 h. Native staining TEM images of RBD **(C)** or S1 **(D)** incubated with L-NPs and D-NPs for 2 h. Cryo-TEM images of RBD **(E)** or S1 **(F)** incubated with L-NPs and D-NPs for 2 h.

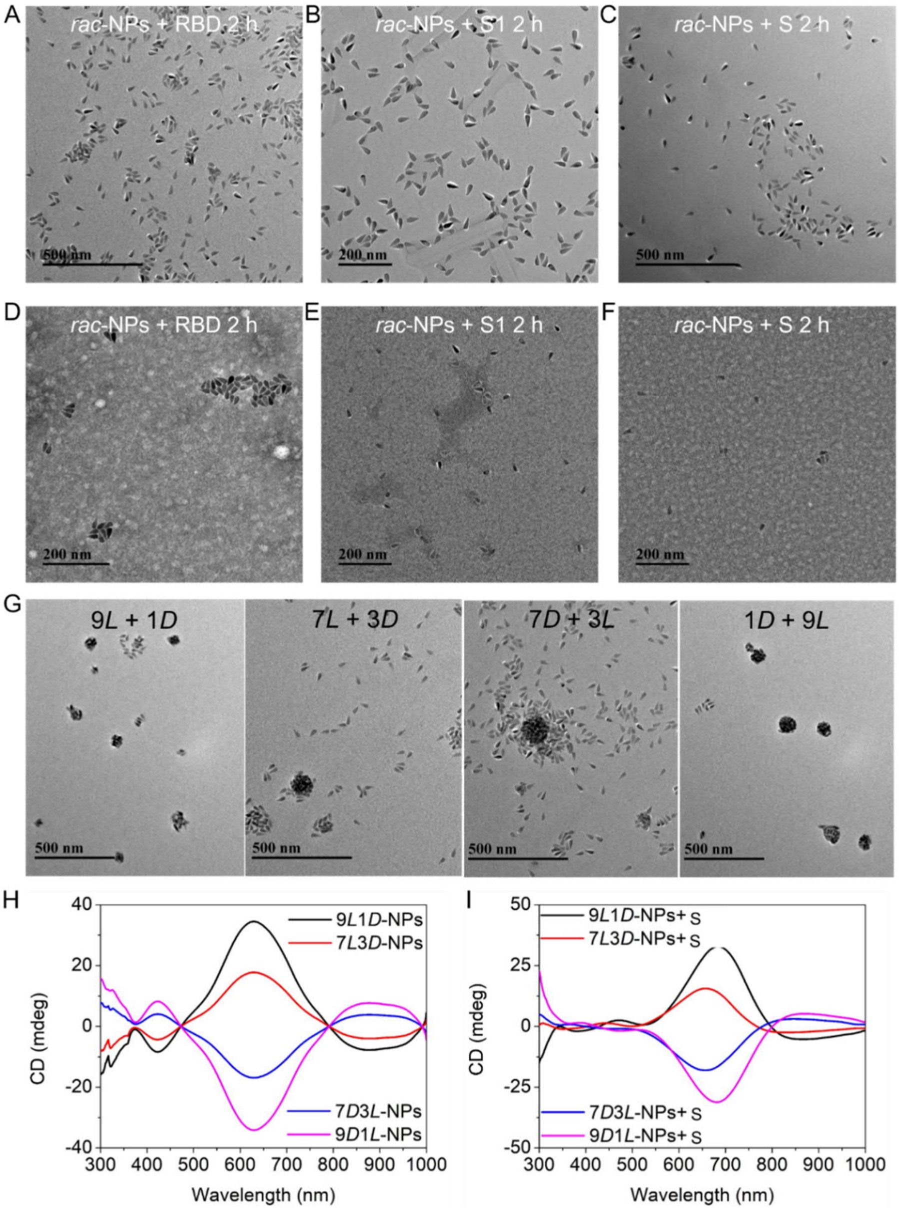

Fig. S12. TEM image of RBD **(A)**, S1 **(B)**, and S **(C)** incubated with rac-NPs for 2 h. **(D)** Native staining TEM image of RBD **(D)**, S1 **(E)** and S **(F)** incubated with rac-NPs for 2 h. **(G)** TEM image of different ratios of L- and D-Pen stabilized NPs incubated with S for 2 h. **(H)** CD spectra of non-racemic NPs. **(I)** CD spectra of non-racemic NPs incubated with S for 6 h.

Comments: The 9L1D means the mixture contains 90% of L-Pen and 10% of D-Pen. 7L3D means the mixture contains 70% of L-Pen and 30% of D-Pen. 7D3L means the mixture contains 70% of D-Pen and 30% of L-Pen. 9D1L means the mixture contains 90% of D-Pen and 10% of L-Pen.

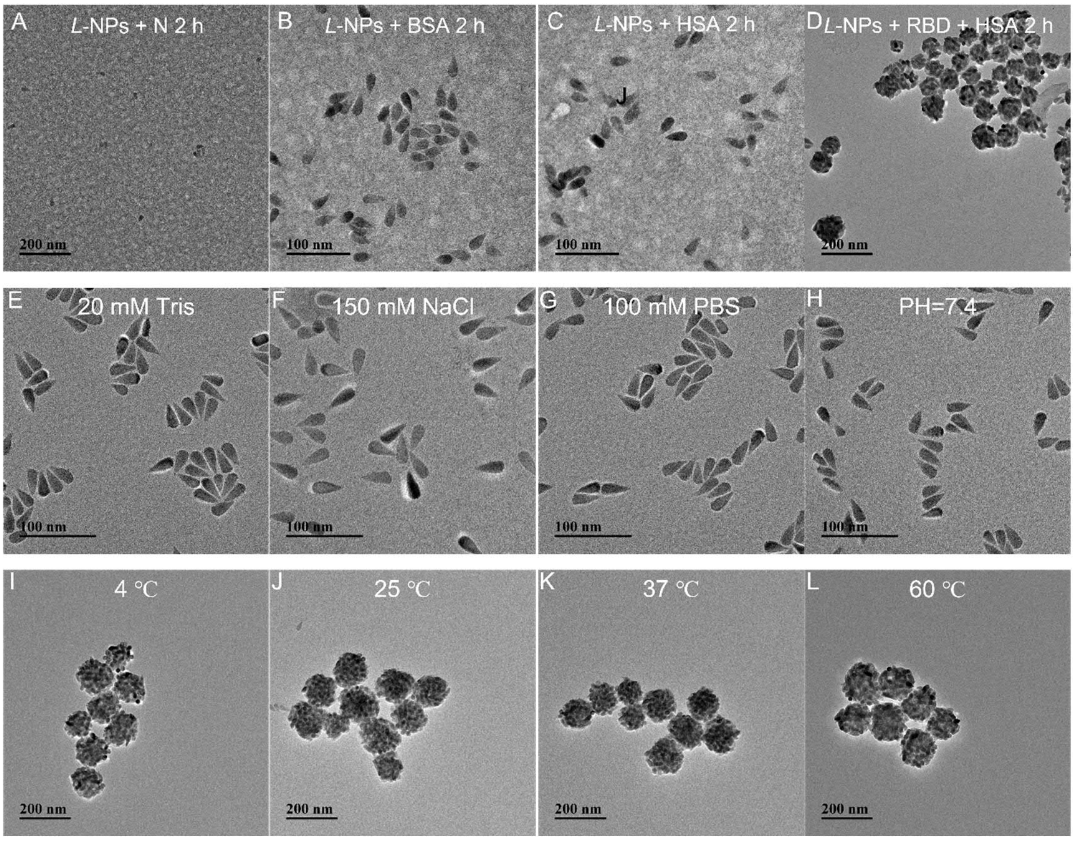

Fig. S13. TEM image of L-NPs was mixed with nucleocapsid protein of SARS-CoV-2 **(A)**, BSA **(B)**, and HSA **(C)** for 2 h. **(D)** TEM image of L-NPs interacted with RBD in the presence of 1 mg/mL HSA to mimic complex biological environment. TEM images of L-NPs were under 20 mM Tris buffer **(E)**, NaCl solution **(F)**, 100 mM PBS buffer **(G)**, and pH 7.4 **(H)**, respectively. TEM images of L-NPs stored at 4 ℃ **(I)**, 25 ℃ **(J)**, 37 ℃ **(K)**, and 60 ℃ **(L)** for 24 h, then incubated with RBD for 2 h, respectively.

Comments: The recombinant S protein, S1 protein, RBD and nucleocapsid were labelled with 10×His tag. The SPs were formed after chiral NPs interacted with recombinant S protein, S1 protein and RBD. On the contrary, no SPs were observed after chiral NPs incubated with nucleocapsid. This indicated that the 6×His tag would not affect the binding between L-NPs and recombinant proteins.

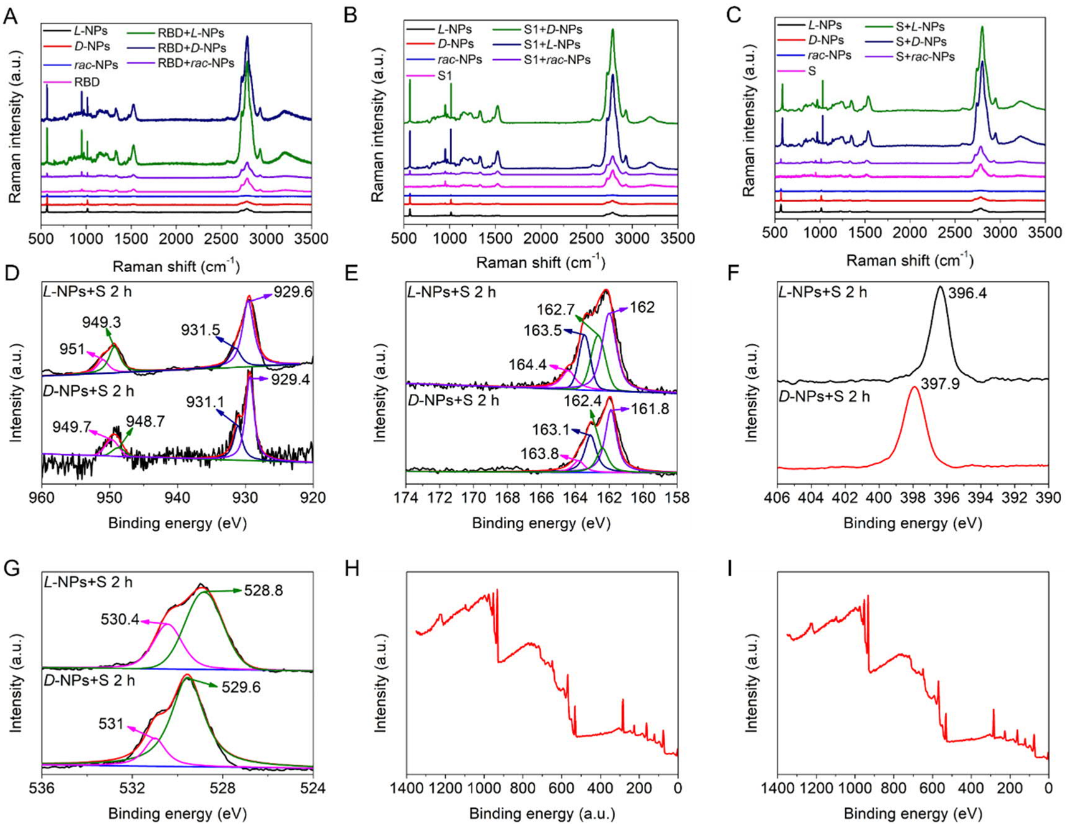

Fig. S14. **(A)** Raman scattering spectra of RBD, and RBD incubated with NPs for 2 h. **(B)** Raman scattering spectra of S1, and S1 incubated with chiral NPs for 2 h. **(C)** Raman scattering spectra of S and S incubated with chiral NPs for 2 h. The binding energy of Cu **(D)**, S **(E)**, N **(F)** and O **(G)** element of two kinds of SPs that comprised chiral NPs and S. The XPS survey of L-NPs **(H)** and D-NPs **(I)** incubated with S.

Comments: 521 cm-1 represents Cu-S bond; 987 cm-1 represents COOH group; 1056 cm-1 represents C-O; 1367 cm-1 and 1601 cm-1 represent amide bond; 2800 cm-1 to 3300 cm-1 represents -C-H and hydrogen bond (41).

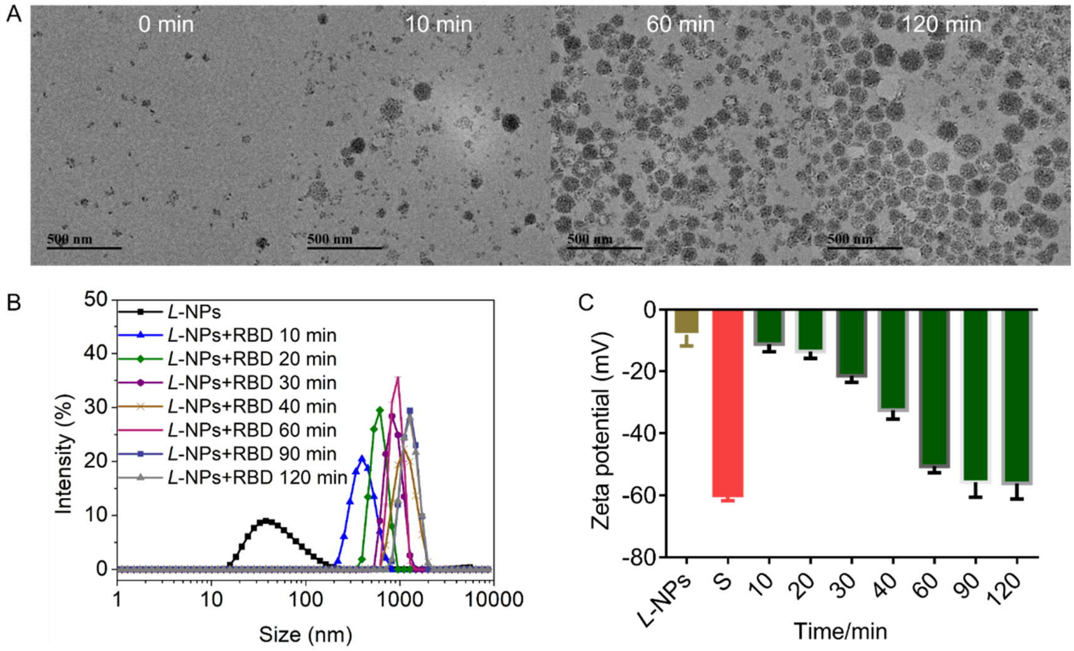

Fig. S15. (A) The TEM images of L-NPs interacted with RBD at different time points. **(B)** The size distribution of L-NPs and L-NPs interacted with RBD at different time points. **(C)** The zeta potential of chiral NPs, RBD and chiral NPs interacted with RBD at different time points. Data are presented as mean ± s.d. (n = 3). The error bars in **Fig. S15C** calculated from standard deviation from three independent experiments.

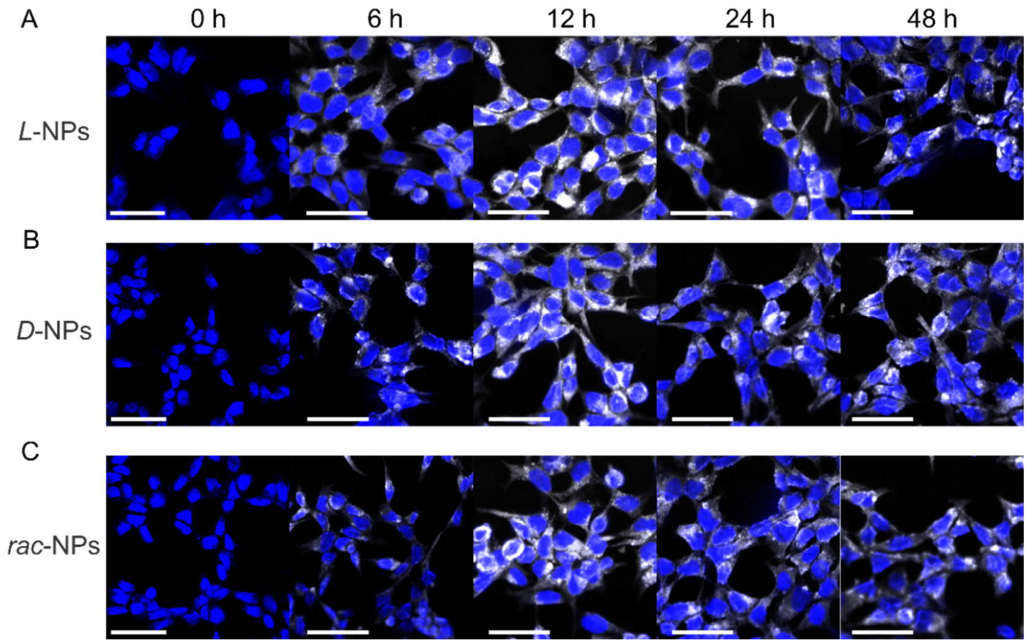

Fig. S16. Confocal images of L-NPs **(A)**, D-NPs **(B)** and rac-NPs **(C)** incubated with HEK293 cells for different times. This result showed that the maximum concentration of chiral NPs in HEK293 cells was observed at 12 h (Scale bar 50 μm).

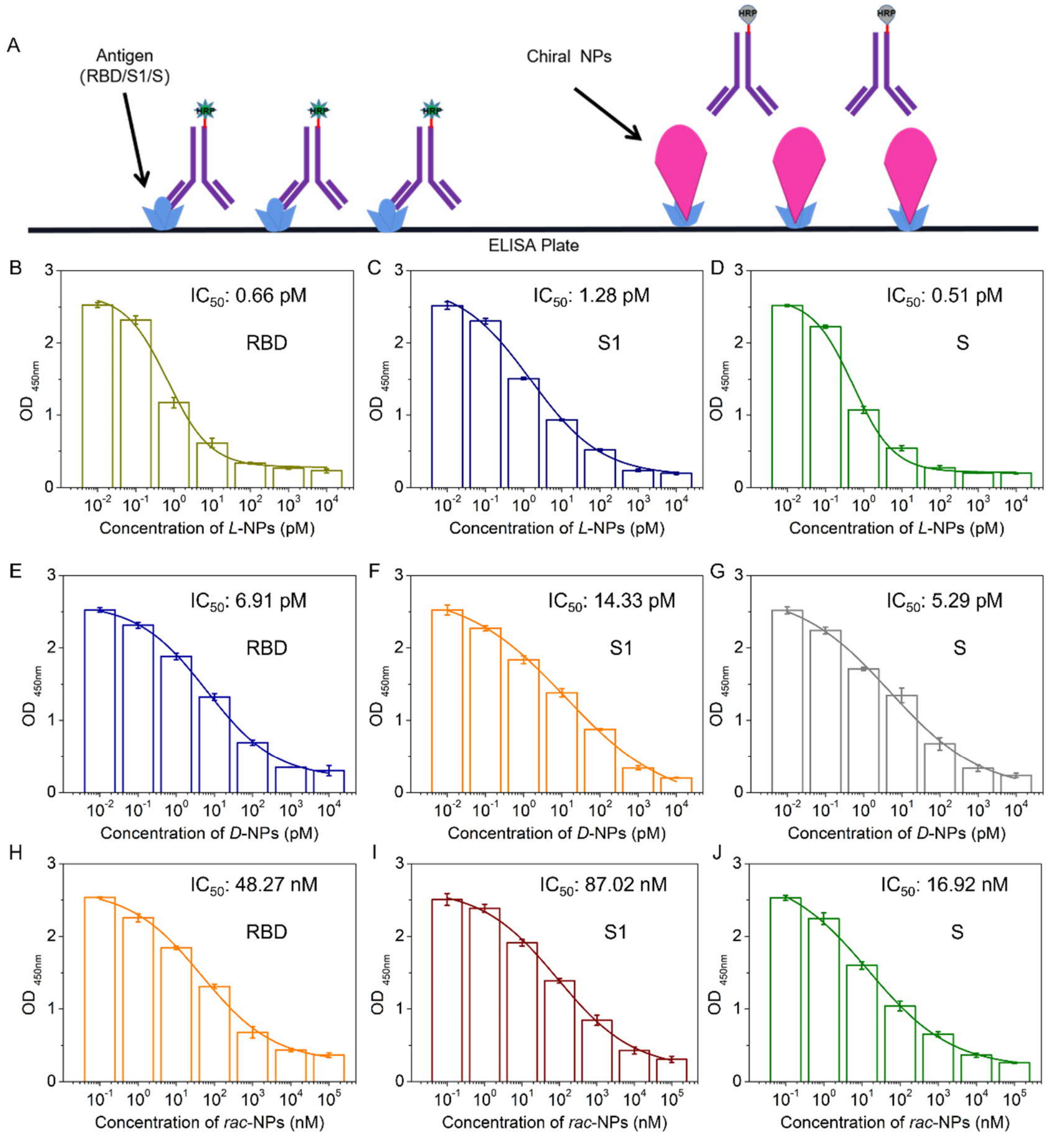

Fig. S17. **(A)** Schematic illustration of neutralizing experiments for SARS-CoV-2 mimics (RBD, S1 and S) by chiral NPs. The bind between chiral NPs and SARS-CoV-2 mimics was assessed by ELISA using anti-SARS-CoV-2 mimics antibody. OD450nm, optical density measurement using a microplate reader with a 450-nm filter. The color titer and curve fitting of RBD/anti-RBD antibody complex, treated with different concentrations of L-NPs **(B)**, D-NPs **(E)**, and rac-NPs **(H)**. The color titer and curve fitting of S1/anti-S1 antibody complex, treated with different concentrations of L-NPs **(C)**, D-NPs **(F)** and rac-NPs **(I)**. The color titer and curve fitting of S/anti-S antibody complex, treated with different concentrations of L-NPs **(D)**, D-NPs **(G)** and rac-NPs **(J)**. Data are presented as mean ± s.d. (n = 3). The error bars in **Figs. S17B-J** calculated from standard deviation from three independent experiments.

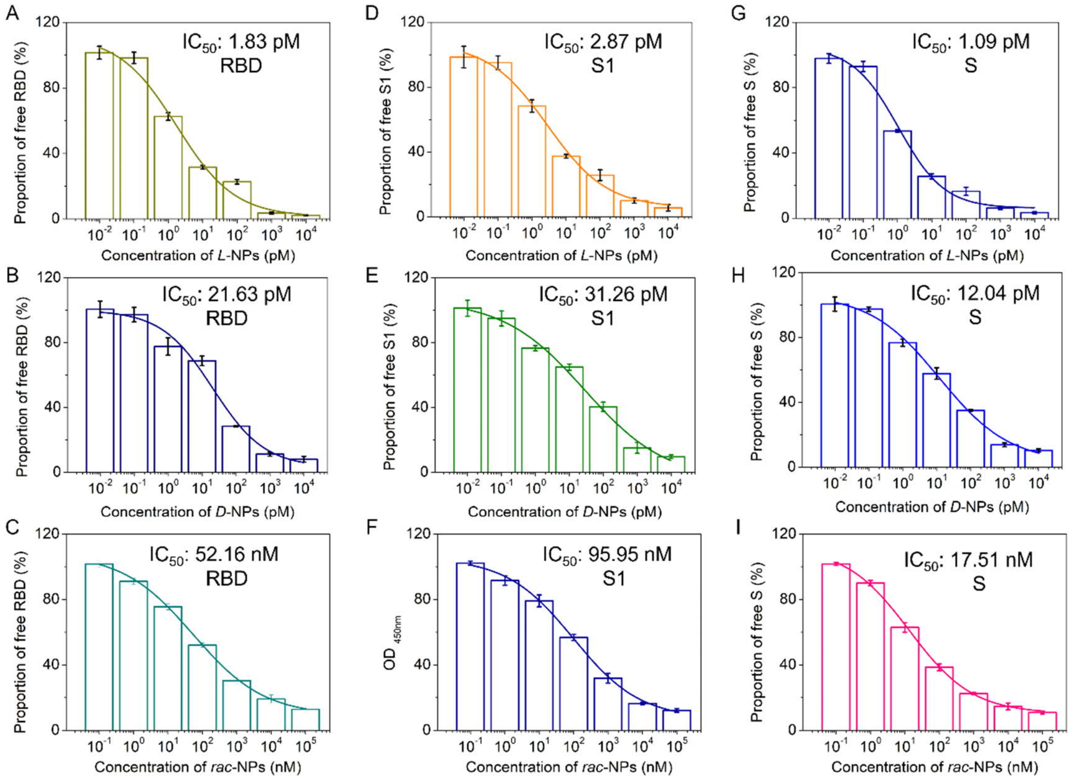

Fig. S18. NP binding to viral components overexpressed in human HEK293 cells. Fitting curves of L-NPs **(A)**, D-NPs **(B)** and rac-NPs **(C)** to RBD. Fitting curves of L-NPs **(D)**, D-NPs **(E)** and rac-NPs **(F)** to S1. Fitting curves of L-NPs **(G)**, D-NPs **(H)** and rac-NPs **(I)** to S. Data are presented as mean ± s.d. (n = 3). The error bars in **Fig. S18** calculated from standard deviation from three independent experiments.

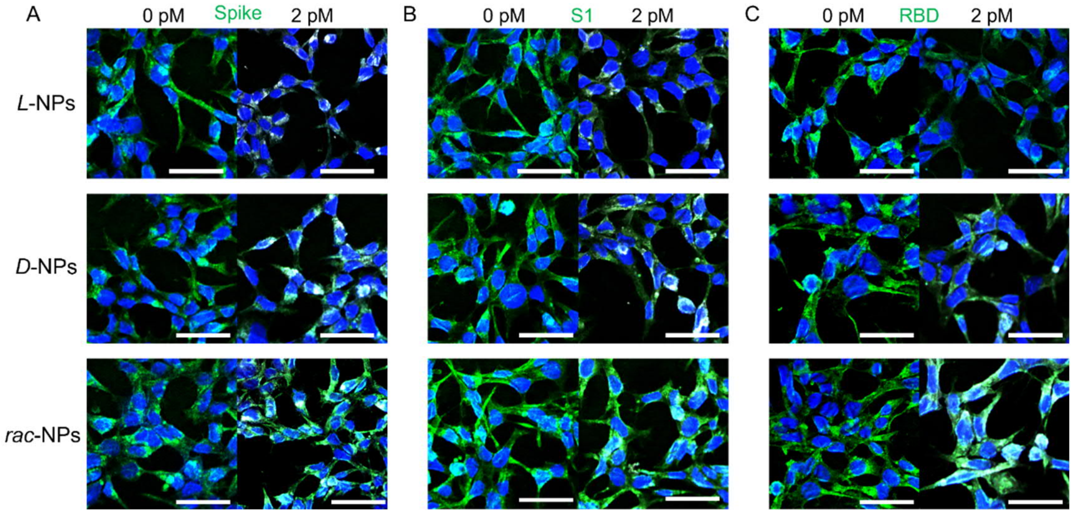

Fig. S19. Confocal images of three kinds of HEK293 cells stably expressing RBD, S1 and S incubated with chiral NPs for 12 h. **(A)** The confocal images of chiral NPs or achiral NPs incubated with Spike-expressed HEK 293 cells for 12 h. The green represents Spike protein using FITC labeled anti-S antibody to imaging. **(B)** The confocal images of chiral NPs or achiral NPs incubated with S1-expressed HEK 293 cells for 12 h. The green represents S1 protein using FITC labeled anti-S1 antibody to imaging. **(C)** The confocal images of chiral NPs or achiral NPs incubated with RBD-expressed HEK 293 cells for 12 h. The green represents RBD using FITC labeled anti-RBD antibody to imaging.

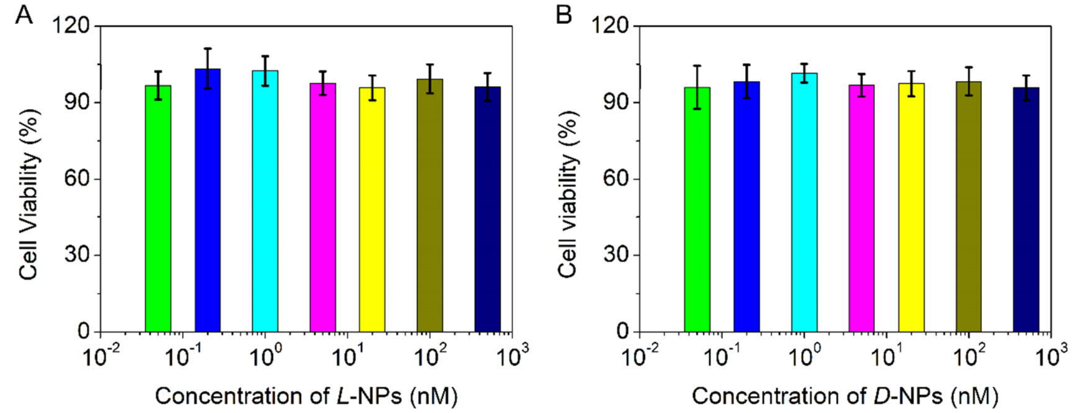

Fig. S20. Cytotoxicity assessment of L-NPs **(A)** and D-NPs **(B)**. Data are presented as mean ± s.d. (n = 3). The error bars in **Fig. S20** calculated from standard deviation from three independent experiments.

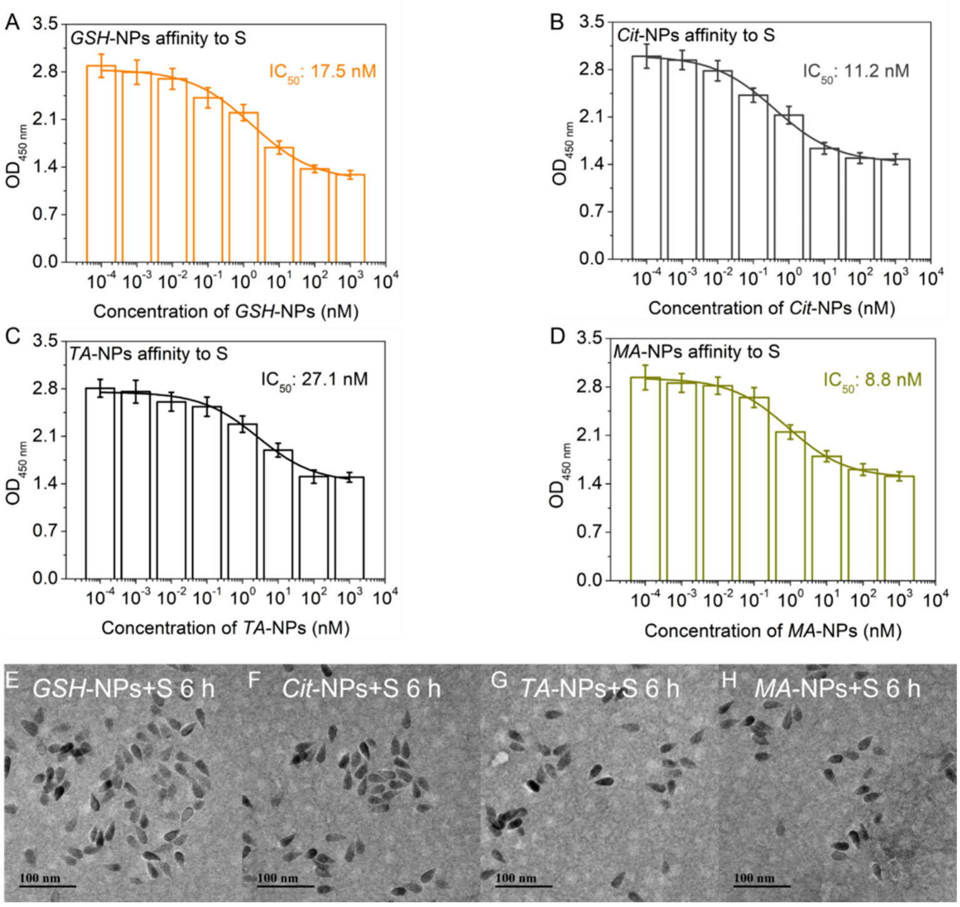

Fig. S21. **(A)** The affinity between Cit-NPs and S evaluated by ELISA-based methods. **(B)** The affinity between GSH-NPs and S evaluated by ELISA-based methods. **(C)** The affinity between TA-NPs and S evaluated by ELISA-based methods. **(D)** The affinity between MA-NPs and S evaluated by ELISA-based methods. TEM images of S incubated with GSH-NPs **(E)**, Cit-NPs **(F)**, TA-NPs **(G)**, and MA-NPs **(H)** for 6 h. Data are presented as mean ± s.d. (n = 3). The error bars in **Figs. S21A-D** calculated from standard deviation from three independent experiments.

Comments: CuS NPs carrying other ligands on their surface such as citric acid, GSH, tartaric acid, and malic acid stabilized showed limited affinity to S with IC50 values 11.2 nM, 17.5 nM, 27.1 nM, and 8.8 nM, respectively. These results showed ca. four orders of magnitude lower than the affinity between L-NPs and S.

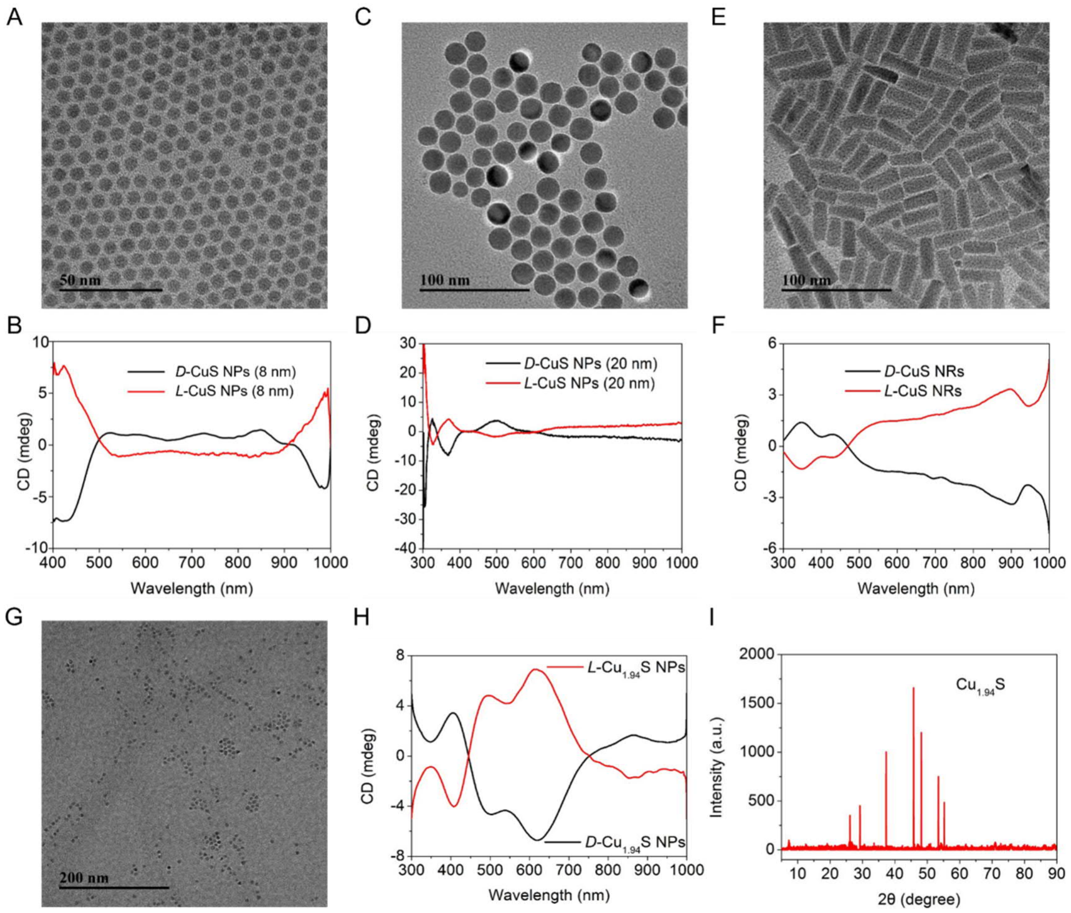

Fig. S22. (A) The TEM image of L-Pen modified the CuS NPs (8 nm). **(B)** The CD spectra of Pen modified the CuS NPs (8 nm). **(C)** The TEM image of L-Pen modified the CuS NPs (20 nm). **(D)** The CD signal of Pen modified the CuS NPs (20 nm). **(E)** The TEM image of L-Pen modified the CuS NRs. **(F)** The CD signal of Pen modified the CuS NRs. **(G)** The TEM image of L-Pen modified the Cu1.94S NPs. **(H)** The CD spectra of Pen modified the Cu1.94S NPs. **(I)** The XRD pattern of L-Pen modified the Cu1.94S NPs.

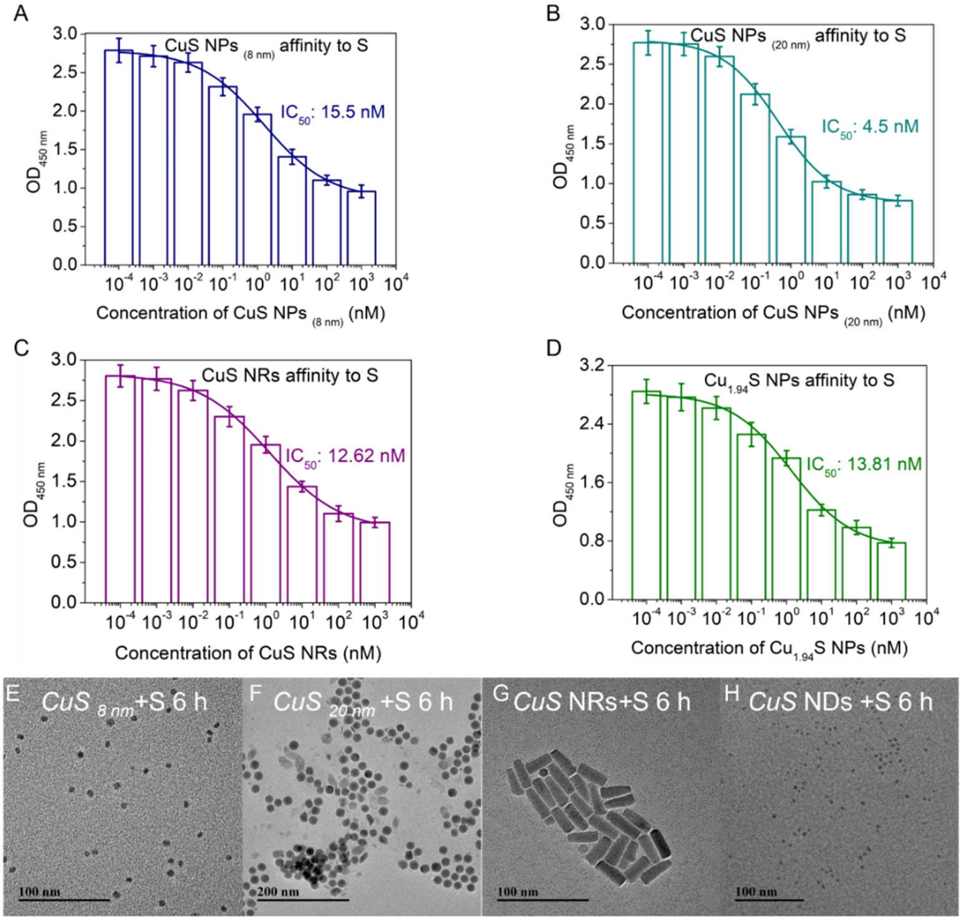

Fig. S23. (A) The affinity between L-Pen modified CuS NPs (8 nm) and RBD evaluated by ELISA-based methods. **(B)** The affinity between L-Pen modified CuS NPs (20 nm) and RBD evaluated by ELISA-based methods. **(C)** The affinity between L-Pen modified CuS NRs and RBD evaluated by ELISA-based methods. **(D)** The affinity between L-Pen modified Cu1.94S NPs and RBD evaluated by ELISA-based methods. TEM images of Spike protein incubated with L-Pen modified CuS NPs (8 nm) **(E)**, L-Pen modified CuS NPs (20 nm) **(F)**, L-Pen modified CuS NRs **(G)**, and L-Pen modified Cu1.94S NPs **(H)** for 6 h. Data are presented as mean ± s.d. (n = 3). The error bars in **Figs. S23A**-D calculated from standard deviation from three independent experiments.

Comments: Cu2-xS NPs with different morphology and functionalized with L-Pen such as CuS NPs with average size of 8 nm and 20 nm, respectively, CuS NRs, and Cu1.94S NPs with average size of 3 nm showed limited affinity to RBD with IC50 values 15.5 mM, 4.5 mM, 12.62 mM, and 13.81 mM, respectively. These results showed four orders of magnitude lower than the affinity between L-NPs and RBD.

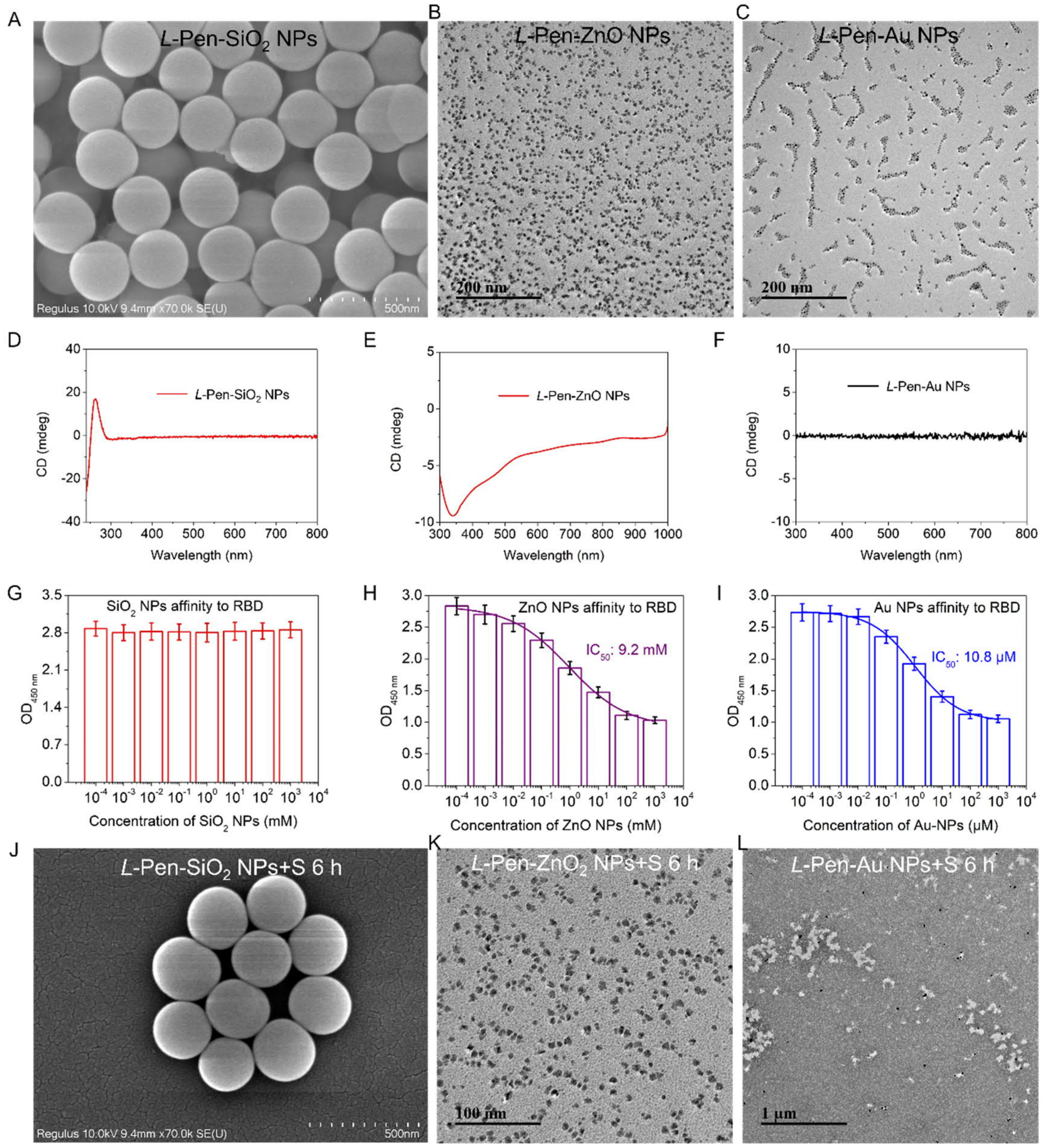

Fig. S24. (A) SEM image of L-Pen stabilized SiO2 NPs. **(B)** TEM image of L-Pen stabilized ZnO NPs. **(C)** TEM image of L-Pen stabilized Au NPs. **(D)** CD spectra of L-Pen stabilized SiO2 NPs. **(E)** CD spectra of L-Pen stabilized ZnO NPs. **(F)** CD spectra of L-Pen stabilized Au NPs. **(G)** The affinity between L-Pen modified SiO2 NPs and RBD evaluated by ELISA. **(H)** The affinity between L-Pen modified ZnO NPs and RBD evaluated by ELISA. **(I)** The affinity between L-Pen modified Au NPs and RBD evaluated by ELISA. **(J)** SEM images of Spike protein incubated with L-Pen stabilized SiO2 NPs, TEM images of Spike protein incubated with L-Pen stabilized ZnO NPs **(K)**, L-Pen stabilized Au NPs **(L)** for 6 h. Data are presented as mean ± s.d. (n = 3). The error bars in **Figs. S24G-I** calculated from standard deviation from three independent experiments.

Comments: Other nanomaterials with different morphology and functionalized with L-Pen such as ZnO NPs and Au NPs showed limited affinity to RBD with IC50 value 9.2 mM and 10.8 mM, respectively, which is about seven orders of magnitude lower affinity than that between L-NPs and RBD. Noted that SiO2 NPs with L-Pen modified showed no affinity to RBD even at very high concentrations.

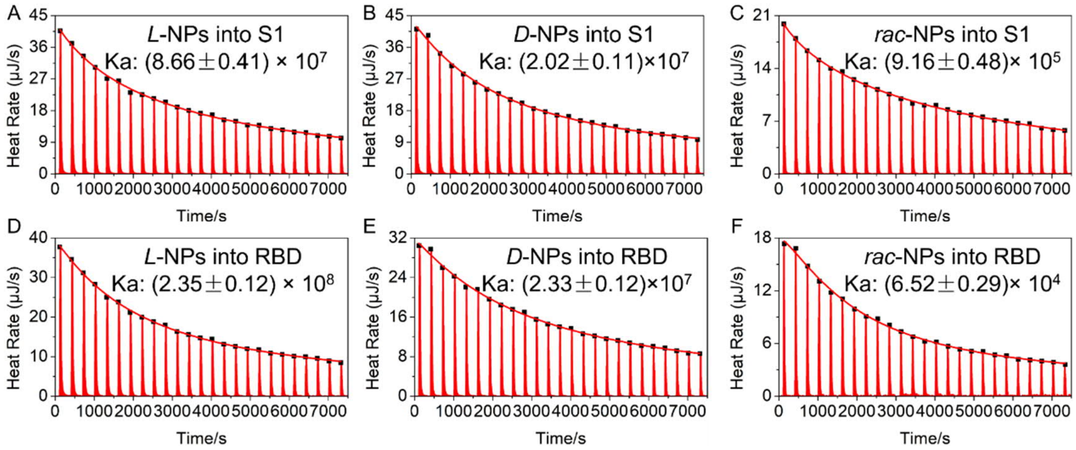

Fig. S25. The ITC data and fitting with thermodynamic models between **(A)** L-NPs and S1; **(B)** D-NPs and S1; **(C)** rac-NPs and S1; **(D)** L-NPs and RBD; **(E)** D-NPs and RBD; and **(F)** rac-NPs and RBD. All the experiments were performed in triplicate.

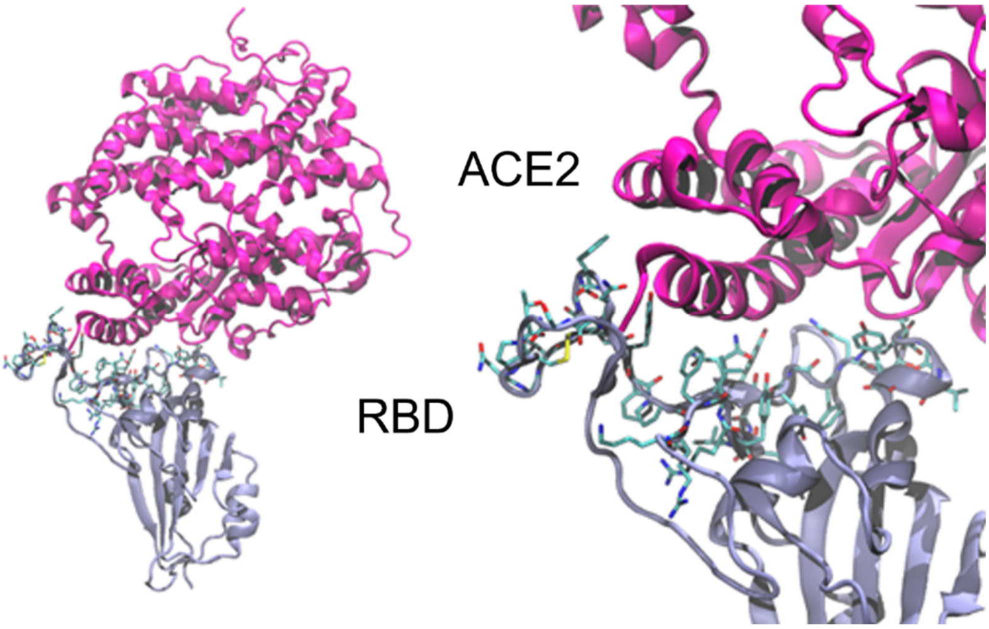

Fig. S26. Structure of the ACE-2 (purple chain) – RBD (blue grey chain) complex (left) from 6m0j structure. RBD residues in direct contact with ACE-2 are highlighted in licorice representation (right).

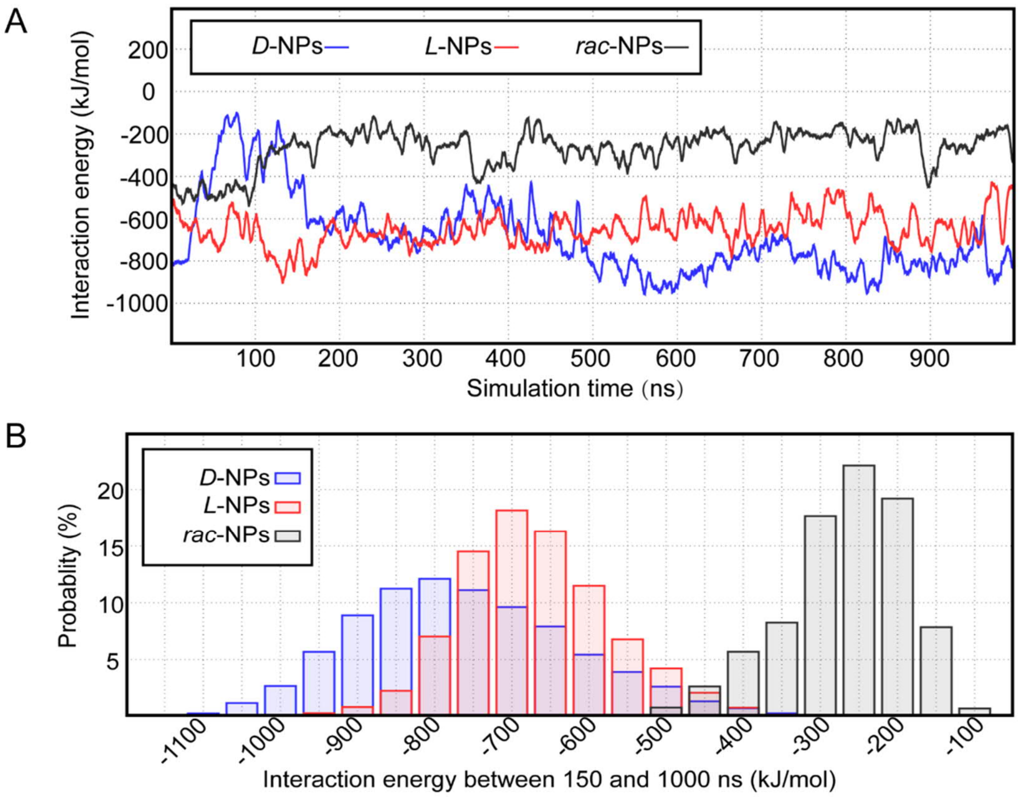

Fig. S27. (A) Interaction energy between one model NP and one RBD unit. **(B)** Distribution of interaction energies after structural relaxation.

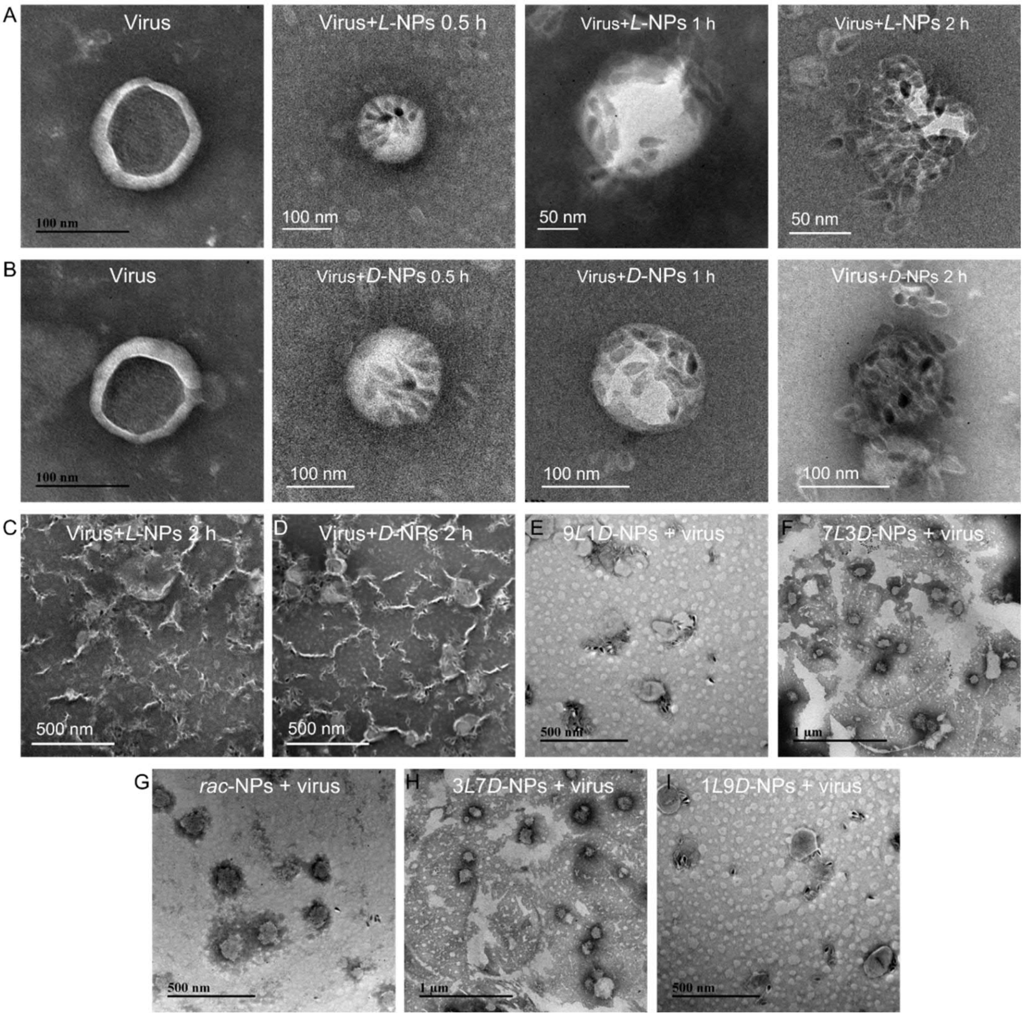

Fig. S28. **(A)** The negative-stained TEM images of SARS-CoV-2 pseudovirus incubated with L-NPs for different time points. **(B)** The negative-stained TEM images of SARS-CoV-2 pseudovirus incubated with D-NPs for different time points. **(C)** Representative negative-stained TEM image of SARS-CoV-2 pseudo-virus incubated with L-NPs for 2 h. **(D)** Negative-stained TEM image of SARS-CoV-2 pseudovirus incubated with D-NPs for 2 h. TEM images of chiral NPs with different L/D ratios of Pen surface ligands 9L1D-NPs **(E)**, 7L3D-NPs **(F)**, rac-NPs **(G)**, 3L7D-NPs **(H)**, and 1L9D-NPs **(I)** incubated with SARS-CoV-2 pseudovirus for 2 h.

Comments: SARS-CoV-2 pseudovirus is covered by chiral NPs when the incubation time was 2 h. The envelope protein is being destroyed after the first hour of incubation. As the co-incubation time reached 2 h, the structure of the pseudovirus was severely disrupted indicating both virustatic activity and virucidal activity of chiral NPs.

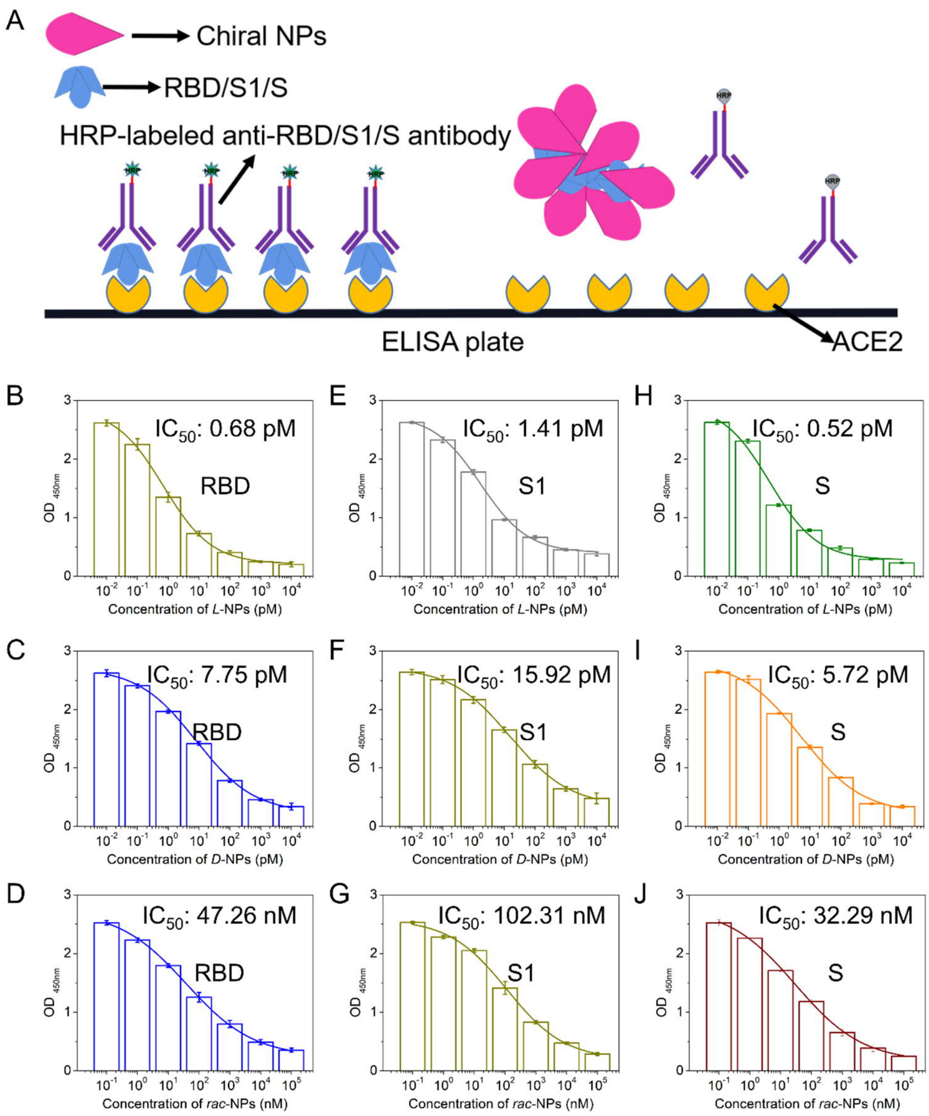

Fig. S29. **(A)** Schematic illustration of chiral NPs inhibiting the binding between RBD/S1/S and ACE2. The binding experiment in vitro between RBD/S1/S and ACE2. The three proteins, RBD **(B)**, S1 **(E)**, and S **(H)** were first incubated with different concentrations of L-NPs for 2 h and then pre-treated RBD, S1 and S proteins were added into ELISA plate that coated ACE2. The three proteins, RBD **(C)**, S1 **(F)**, and S **(I)** were first incubated with different concentration of D-NPs for 2 h and then pre-treated RBD, S1 and S proteins were added into ELISA plate that coated ACE2. The three proteins, RBD **(D)**, S1 **(G)** and S **(J)** were first incubated with different concentrations of rac-NPs for 2 h and then pre-treated RBD, S1 and S proteins were added into ELISA plate that coated ACE2. Data are presented as mean ± s.d. (n = 3). The error bars in **Figs. S29B-J** calculated from standard deviation of three independent experiments.

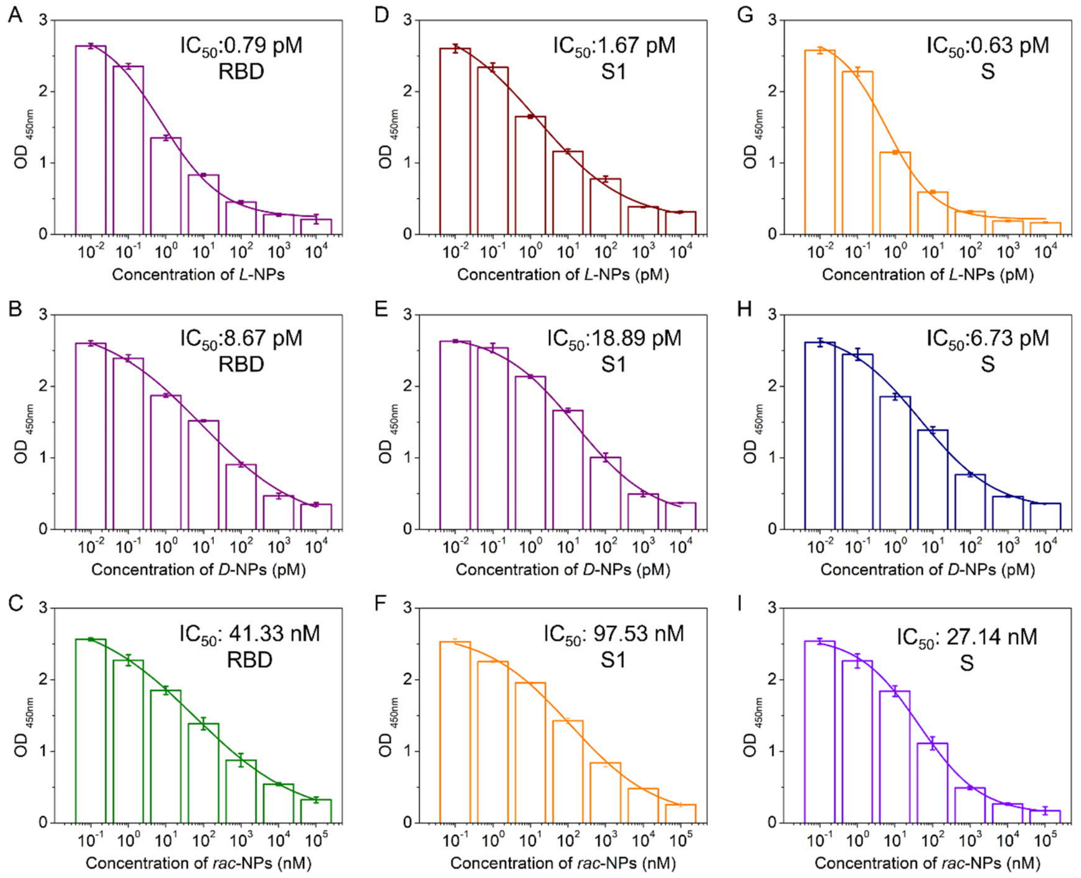

Fig. S30. The in vitro binding experiment between RBD/S1/S and ACE2. (A-I) The mixture of RBD and L-NPs **(A)**, RBD and D-NPs **(B)**, RBD and rac-NPs **(C)**, S1 and L-NPs **(D)**, S1 and D-NPs **(E)**, S1 and rac-NPs **(F)**, S and L-NPs **(G)**, S and D-NPs **(H)**, S and rac-NPs **(I)** were added into ELISA plate that coated ACE2. The binding reactivity was assessed by ELISA using anti-RBD, anti-S1, and anti-S antibody, respectively. OD450, optical density measurement using a microplate reader with a 450-nm filter. Data are presented as mean ± s.d. (n = 3). The error bars in **Fig. S30** calculated from standard deviation from three independent experiments.

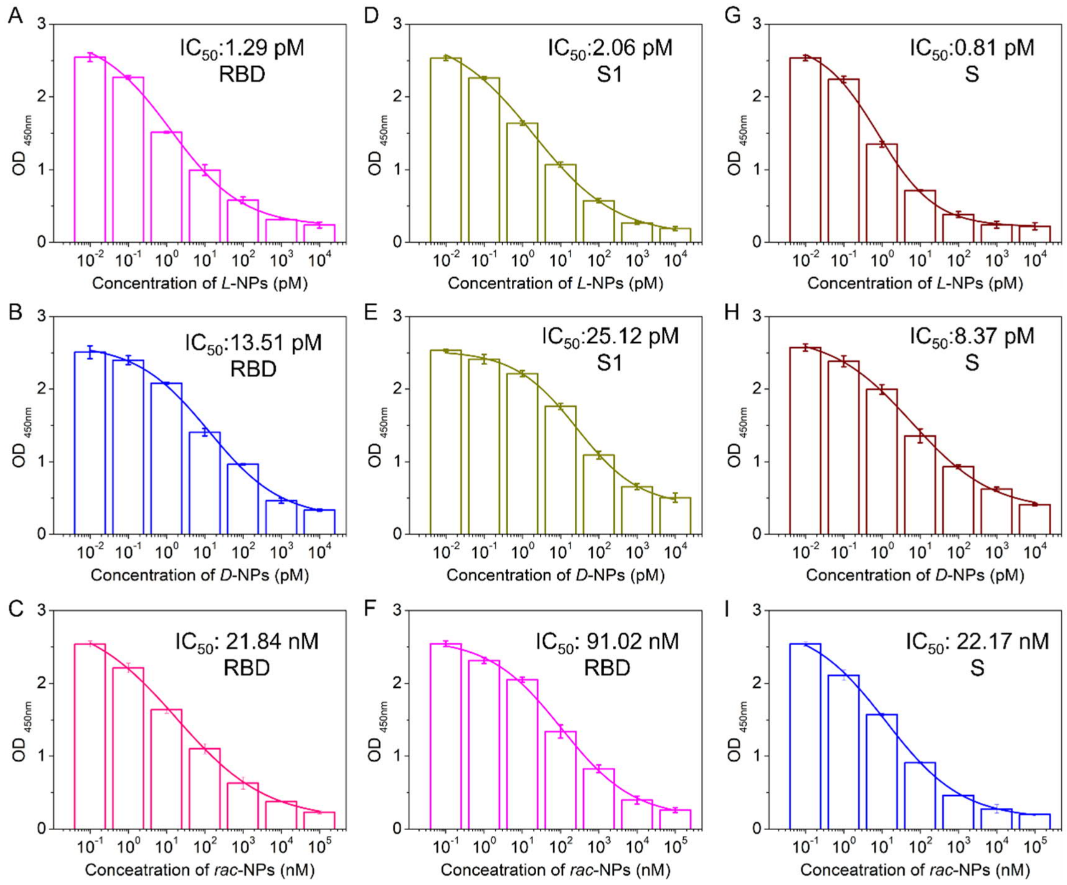

Fig. S31. The binding experiment in vitro between RBD/S1/S and ACE2. (A-I) The three proteins, RBD, S1 and S protein were first added into ELISA plate that coated ACE2 to form RBDACE complex (A-C), S1 and ACE2 complex (D-F) and S and ACE2 complex (G-I) and then different concentration of L-NPs or D-NPs or rac-NPs was added into ELISA plate that contained RBD and ACE complex, S1 and ACE2 complex and S and ACE2 complex, respectively. The binding reactivity was assessed by ELISA using anti-RBD, anti-S1 and anti-S antibody, respectively. OD450, optical density measurement using a microplate reader with a 450-nm filter. Data are presented as mean ± s.d. (n = 3). The error bars in **Fig. S31** calculated from standard deviation from three independent experiments.

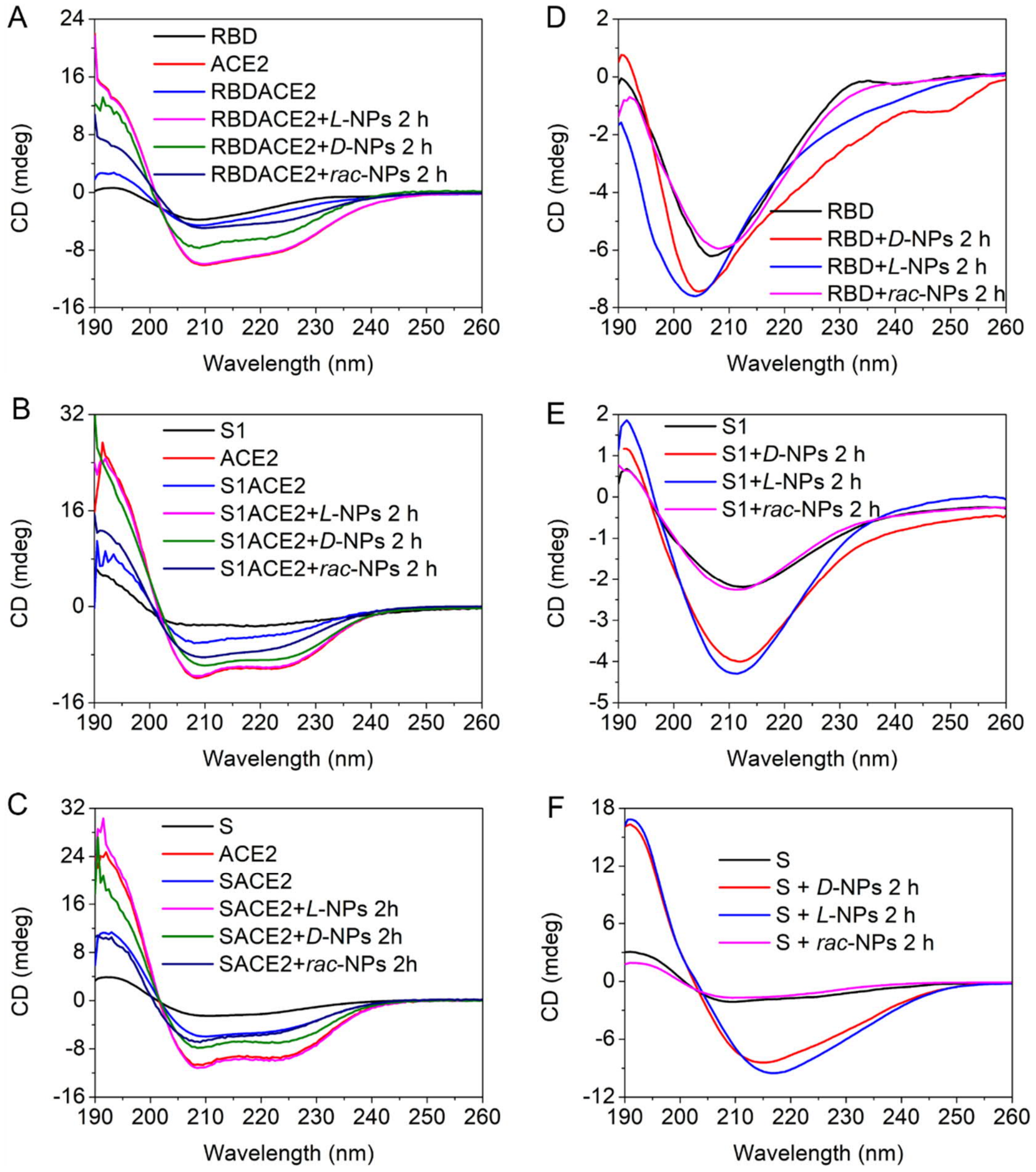

Fig. S32. The chiral NPs inhibited the binding between RBD/S1/S and ACE2. **(A)** The CD spectra of RBD, RBDACE2 complex before and after they interacted with L-NPs, D-NPs and rac-NPs for 2 h. **(B)** The CD spectra of S1, S1ACE2 complex before and after they interacted with L-NPs, D-NPs and rac-NPs for 2 h. **(C)** The CD spectra of S, SACE2 complex before and after they interacted with L-NPs, D-NPs and rac-NPs for 2 h. **(D)** CD spectra of RBD before and after it interacted with L-NPs, D-NPs and rac-NPs for 2 h. **(E)** The CD spectra of S1 before and after it interacted with L-NPs, D-NPs and rac-NPs for 2 h. **(F)** The CD spectra of S before and after it interacted with L-NPs, D-NPs and rac-NPs for 2 h.

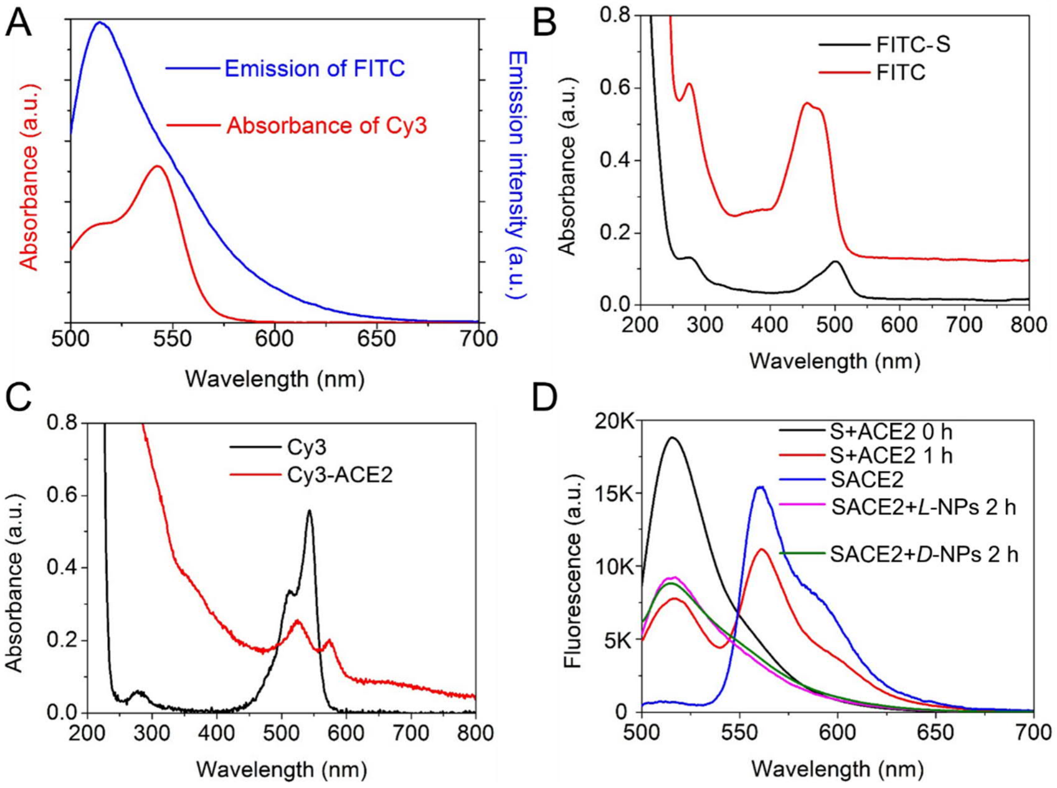

Fig. S33. (A) The absorbance of FITC and FITC labeled S protein. **(B)** The absorbance of Cy3 and Cy3 labeled ACE2. **(C)** The emission of FITC, and the absorbance of Cy3. **(D)** The FRET between S and ACE2, and the FRET was inhibited by chiral NPs.

Comments: We labeled the S with FITC (FITC-S), and the ACE2 was labeled with Cy3 (Cy3-ACE2) to from a FRET pair. After the FITC-S mixed with Cy3-ACE2 at 37 ℃ for 2 h, the fluorescence of FITC was decreased significantly and Cy3 signal was observed under FITC excitation wavelength due to the binding between S and ACE2. But when the chiral NPs were added into the SACE2 complex solution. The fluorescence of FITC was recovered and no Cy3 signal was monitored. Those results further proved chiral NPs could block the binding between SARS-CoV-2 mimics and ACE2.

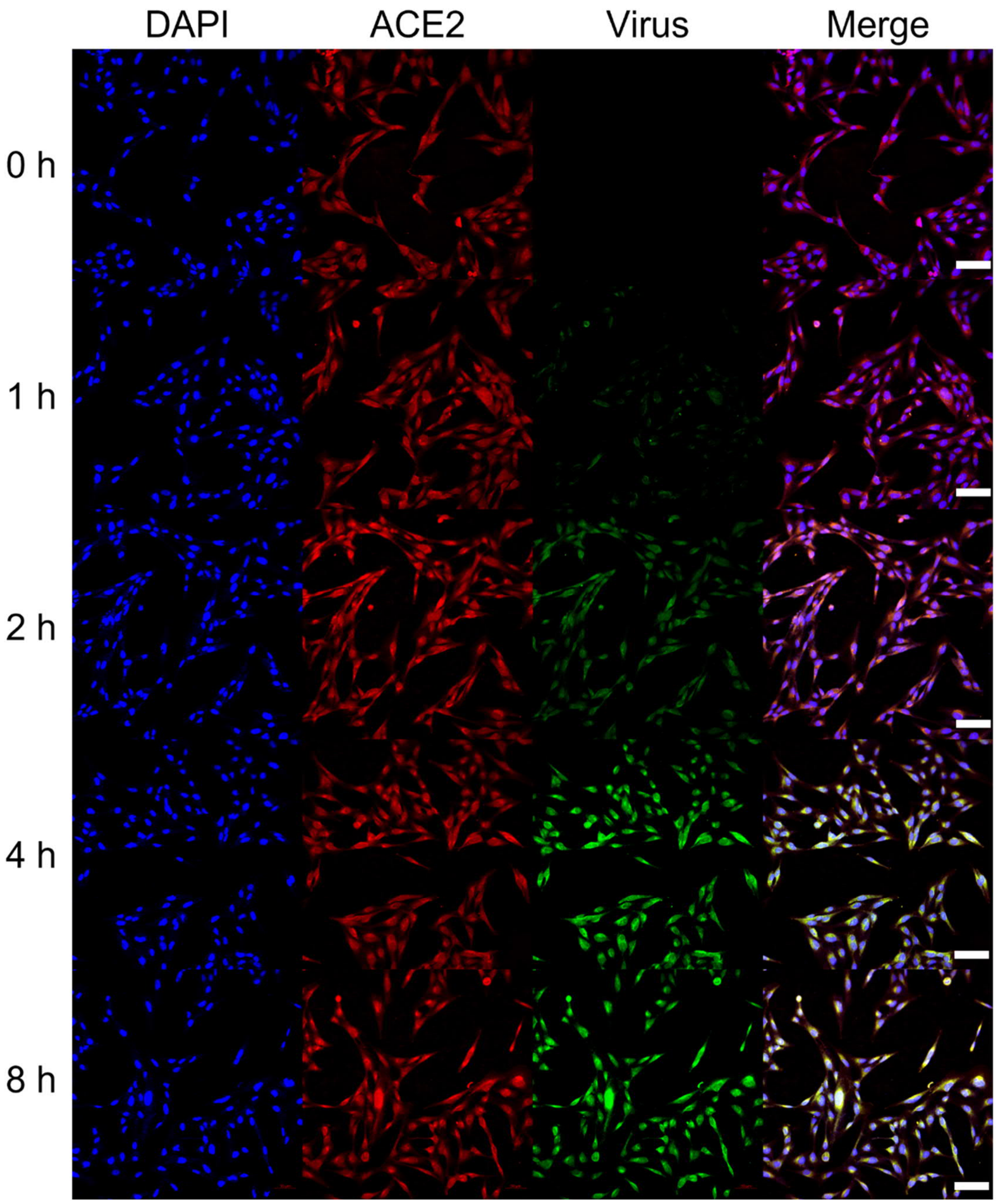

Fig. S34. Confocal images of BEAS-2B cells incubated with pseudovirus for different time (scale bar: 50 μm).

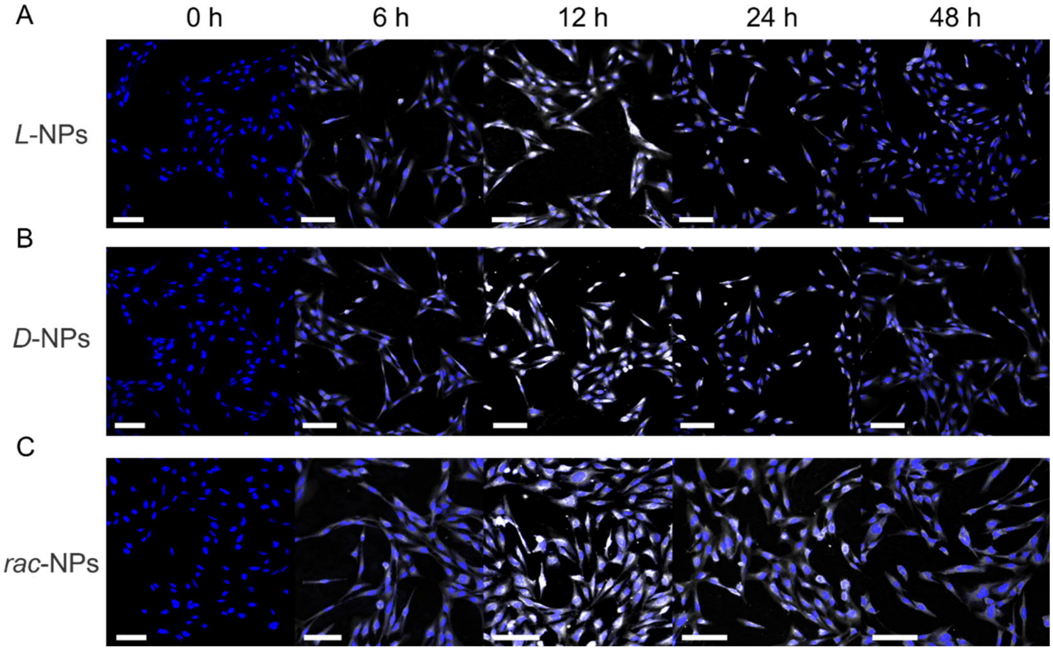

Fig. S35. Confocal images of BEAS-2B cells incubated with L-NPs **(A)**, D-NPs **(B)** and rac-NPs **(C)** for different time (scale bar: 50 μm).

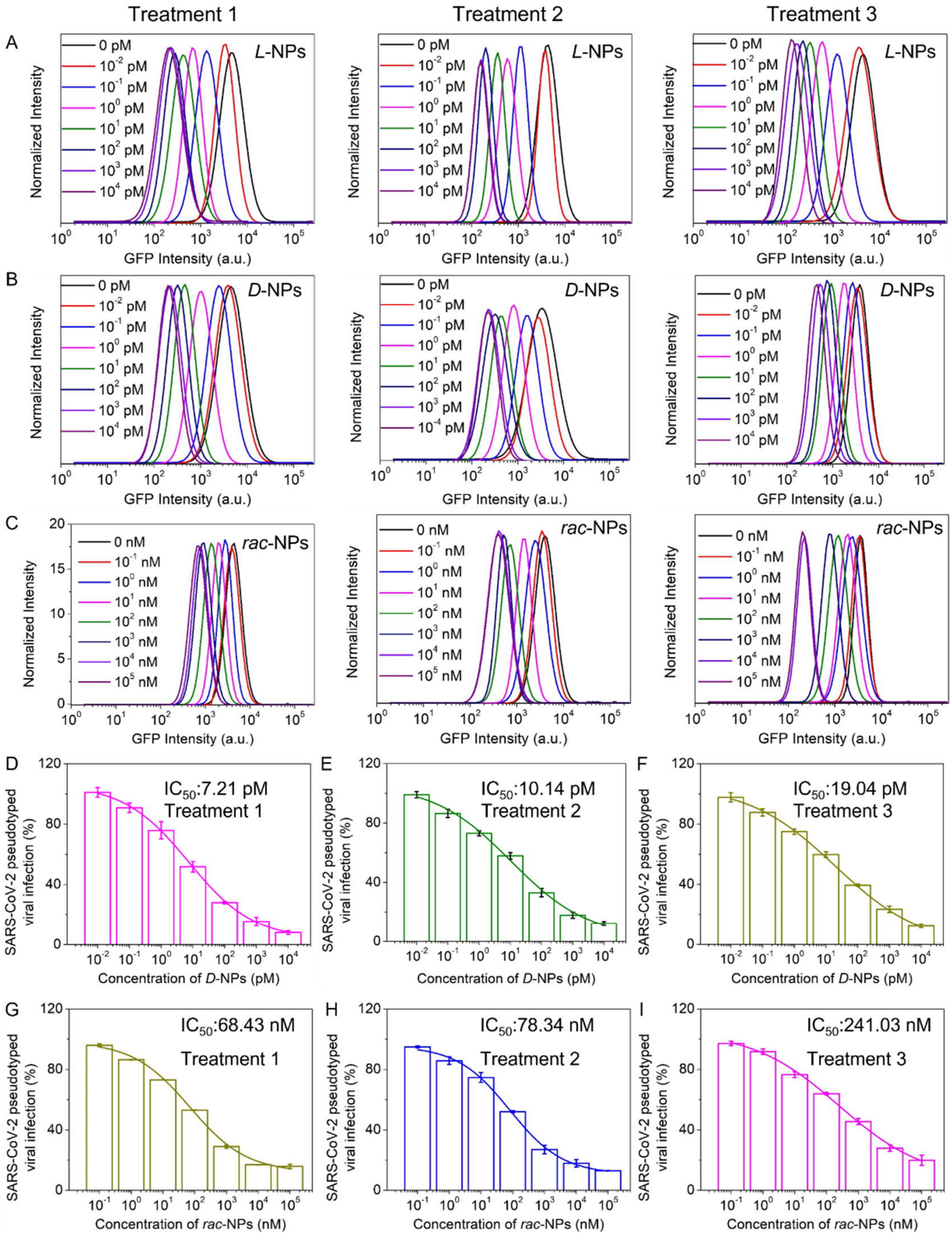

Fig. S36. Chiral NPs neutralize pseudotype SARS-CoV-2. The pseudovirus neutralization results for L-NPs **(A)**, D-NPs **(B)**, and rac-NPs **(C)** for different treatments reflecting either prophylactic or treatment modalities. Pseudovirus neutralization when D-NPs **(D)** or rac-NPs **(G)** pre-incubated with pseudovirus and then incubated with BEAS-2B cells. Pseudovirus neutralization results when D-NPs **(E)** or rac-NPs **(H)** co-incubated with pseudovirus and then were incubated with BEAS-2B cells. Pseudovirus neutralization when D-NPs **(F)** or rac-NPs **(I)** were incubated with BEAS-2B cells that were pre-incubated with pseudovirus. Data are presented as mean ± s.d. (n = 3). The error bars in **Figs. S36D-I** calculated from standard deviation from three independent experiments.

Comments:

Treatment 1. The pseudovirus (20 μL, 1.0×107) first incubated with different concentration of chiral or achiral NPs for 2 h and then pre-treated pseudovirus was added into BEAS-2B cells (4×105) for 24 h. After that the cells were collected via centrifuge (1200 rpm, 3 min) for quantification by flow cytometry (Signals were collected from 1×104 cells).
Treatment 2. The mixture contained pseudovirus (20 μL, 1.0×107) and different concentration of chiral or achiral NPs were added into BEAS-2B cells (4×105) for 24 h. After that the cells were collected via centrifuge (1200 rpm, 3 min) for quantification by flow cytometry (Signals were collected from 1×104 cells).
Treatment 3. The pseudovirus (20 μL, 1.0×107) were first added into BEAS-2B cells (4×105) for 1 h and then different concentration of chiral or achiral NPs were added into infected BEAS-2B cells for 24 h. After that the cells were collected via centrifuge (1200 rpm, 3 min) for quantification by flow cytometry (Signals were collected from 1×104 cells).

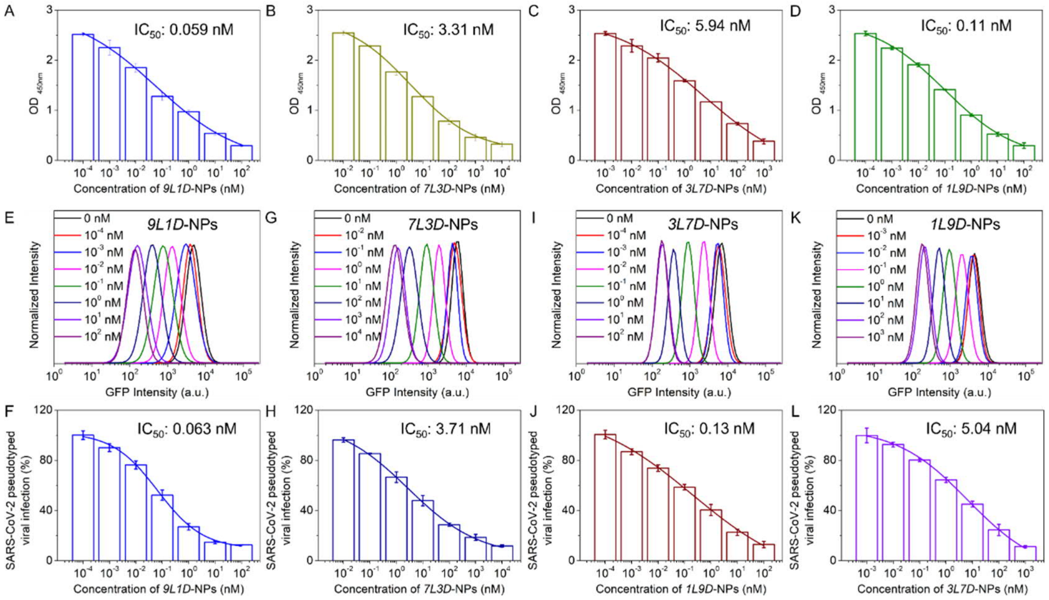

Fig. S37. The inhibitory effect of binding event between ACE2 and SARS-CoV-2 pseudovirus by 9L1D-NPs **(A)**, 7L3D-NPs **(B)**, 3L7D-NPs **(C)**, 1L9D-NPs **(D)**, respectively in buffer using ELISA. GFP intensity of BEAS-2B cells after being infected by GFP-SARS-CoV-2 pseudovirus treated with 9L1D-NPs **(E)** and fitting curve result **(F)** between GFP intensity and 9L1D-NPs concentration. GFP intensity of BEAS-2B cells after being infected by GFP-SARS-CoV-2 pseudovirus treated with 7L3D-NPs **(G)** and fitting curve result **(H)** between GFP intensity and 7L3D-NPs concentration. GFP intensity of BEAS-2B cells after being infected by GFP-SARS-CoV-2 pseudovirus treated with 3L7D-NPs **(I)** and fitting curve result **(J)** between GFP intensity and 3L7D-NPs concentration. GFP intensity of BEAS-2B cells after being infected by GFP-SARS-CoV-2 pseudovirus treated with 1L9D-NPs **(K)** and fitting curve result **(L)** between GFP intensity and 1L9D-NPs concentration. Data are presented as mean ± s.d. (n = 3). The error bars in **Fig. S37** calculated from standard deviation from three independent experiments.

Comments: The 9L1D means that a mixture for NP ligand exchange contained 90% of L-Pen and 10% of D-Pen. 7L3D means that a mixture for NP ligand exchange contained 70% of L-Pen and 30% of D-Pen. 3L7D means that a mixture for NP ligand exchange contained 30% of L-Pen and 70% of D-Pen. 1L9D means that a mixture for NP ligand exchange contained 10% of L-Pen and 90% of D-Pen. As the proportion of D-Pen mixed with L-Pen increases, the IC50 value for inhibition of SARS-CoV-2 pseudovirus has increased.

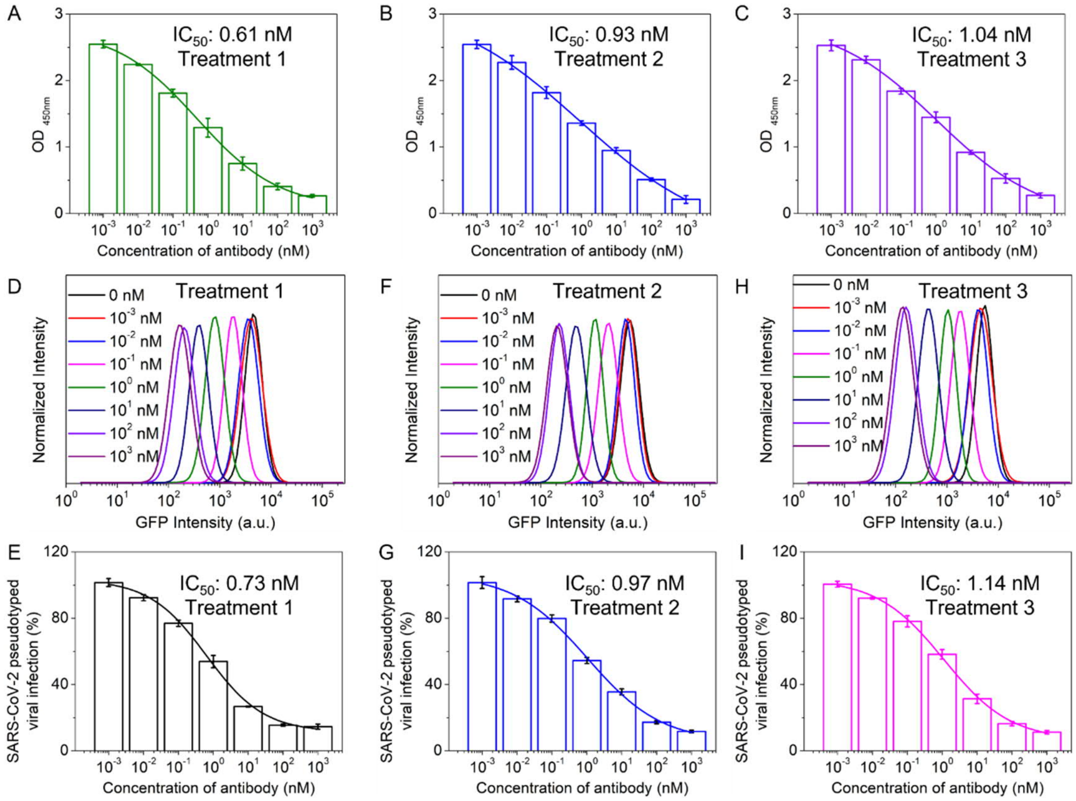

Fig. S38. The ELISA experiment in vitro of antibody to inhibit binding between pseudovirus and ACE2 under Treatment 1 **(A)**, Treatment 2 **(B)** and Treatment 3 **(C)**. The pseudovirus was treated with different conditions and then added into ELISA plate that coated ACE2. The binding reactivity was assessed by ELISA using anti-S antibody. OD450, optical density measurement using a microplate reader with a 450-nm filter. GFP intensity of BEAS-2B cells after being infected by GFP-SARS-CoV-2 pseudovirus treated with antibody under Treatment 1 protocol **(D)** and fitting curve result **(E)** between GFP intensity and antibody concentration. GFP intensity of BEAS-2B cells after being infected by GFP-SARS-CoV-2 pseudovirus treated with antibody under Treatment 2 protocol **(F)** and fitting curve result **(G)** between GFP intensity and antibody concentration. GFP intensity of BEAS-2B cells after being infected by GFP-SARS-CoV-2 pseudovirus treated with antibody under Treatment 3 protocol **(H)** and fitting curve result **(I)** between GFP intensity and antibody concentration. Data are presented as mean ± s.d. (n = 3). The error bars in **Fig. S38** calculated from standard deviation from three independent experiments.

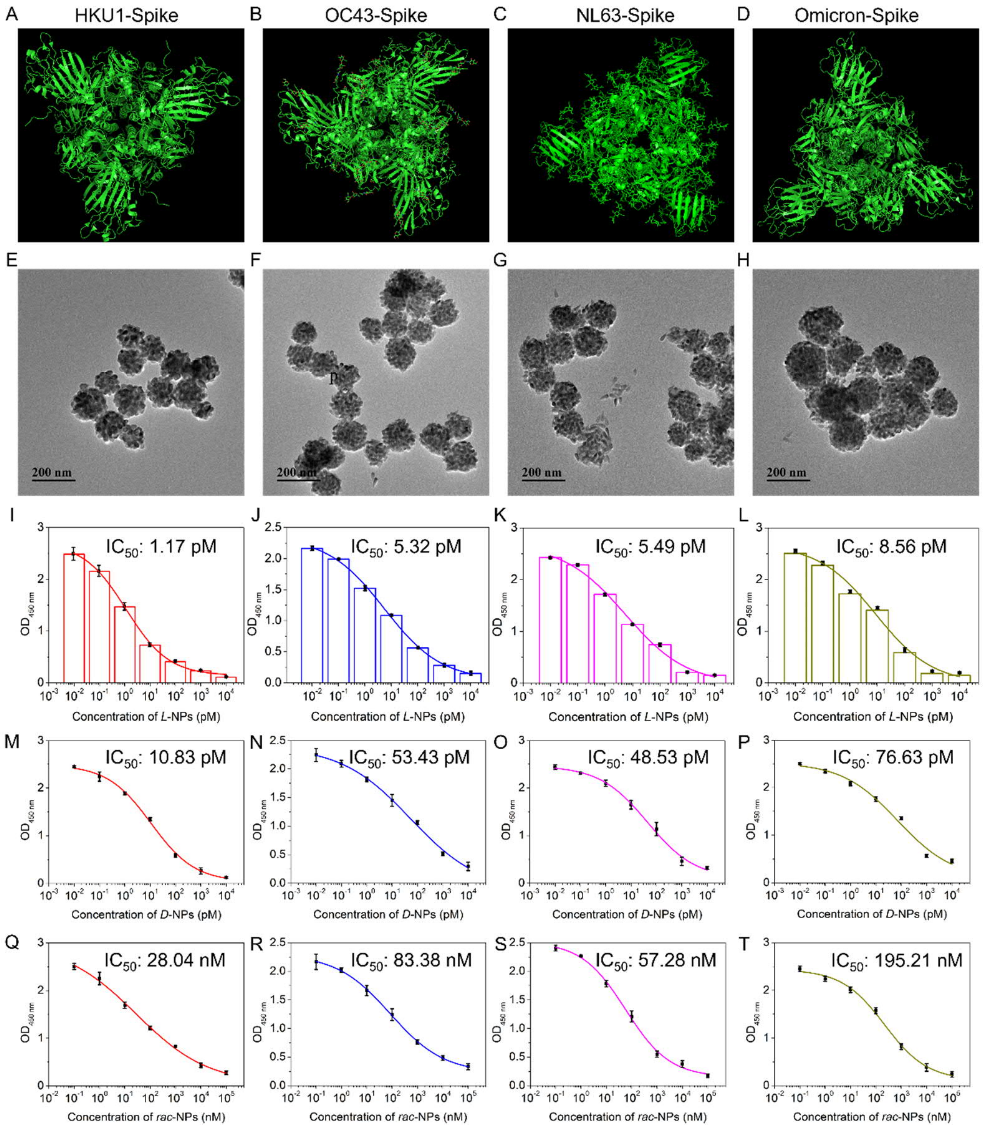

Fig. S39. Crystal structures of HCoV-HKU1 Spike **(A)**, HCoV-OC43 Spike **(B)**, HCoV-NL63 Spike **(C)** and SARS-CoV-2 omicron (B.1.1.529) variant spike protein **(D)**. TEM images of L-NPs incubated with HCoV-HKU1 spike protein **(E)**, HCoV-OC43 Spike **(F)**, HCoV-NL63 spike protein **(G)** and SARS-CoV-2 omicron variant spike protein for 2 h **(H)**. The half-maximal inhibitory concentration (IC50) value of L-NPs against HCoV-HKU1 spike **(I)**, HCoV-OC43 spike **(J)**, HCoV-NL63 spike **(K)** and SARS-CoV-2 omicron variant spike **(L)** evaluated by ELISA-based methods. The IC50 value of D-NPs against HCoV-HKU1 spike **(M)**, HCoV-OC43 spike **(N)**, HCoV-NL63 spike **(O)** and SARS-CoV-2 omicron variant spike **(P)** evaluated by ELISA-based methods. The IC50 value of rac-NPs against HCoV-HKU1 spike **(Q)**, HCoV-OC43 spike **(R)**, HCoV-NL63 spike **(S)** and SARS-CoV-2 omicron variant spike **(T)** evaluated by ELISA-based methods. Data are presented as mean ± s.d. (n = 3). The error bars in **Figs. S39 I-T** calculated from standard deviation by three independent experiments.

Comments: The crystal structures of HCoV-HKU1 spike protein, HCoV-OC43 spike protein, HCoV-NL63 spike protein and SARS-CoV-2 omicron (B.1.1.529) variant spike protein were obtained from PDB:5I08, 6OHW, 6OHW and 7WVN, respectively. L-NPs showed outstanding broad-spectrum for HCoV-HKU1 spike protein, HCoV-OC43 spike protein, HCoV-NL63 S spike protein and SARS-CoV-2 omicron variant spike protein with IC50 value as low as 1.17 pM. 5.32 pM. 5.49 pM and 8.56 pM, respectively.

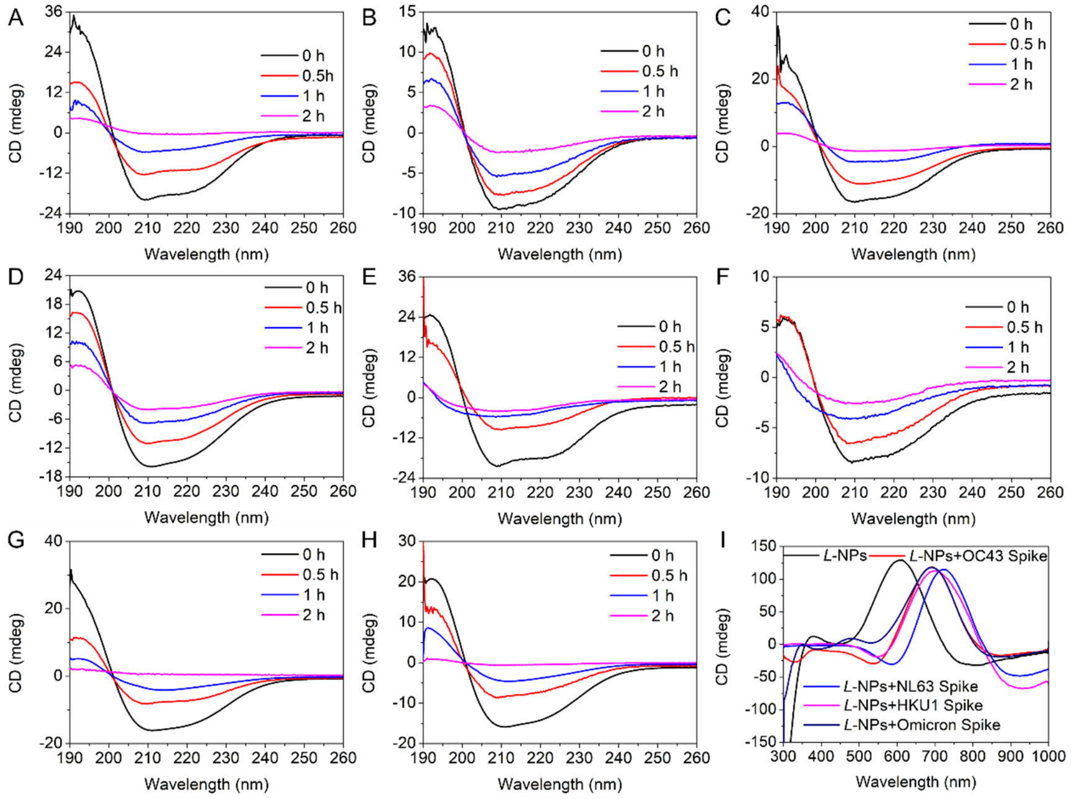

Fig. S40. The CD spectra of supernatant obtained from HCoV-HKU1 Spike **(A)**, HCoV-OC43 Spike **(B)**, HCoV-NL63 Spike **(C)** and SARS-CoV-2 omicron variant spike protein **(D)** incubated with L-NPs at different time points. The CD spectra of precipitate obtained from HCoV-HKU1 spike protein **(E)**, HCoV-OC43 spike protein **(F)**, HCoV-NL63 spike protein **(G)** and SARS-CoV-2 omicron variant spike protein **(H)** incubated with L-NPs at different time points. **(I)** The CD spectra of L-NPs after interacted with four Spike proteins, respectively.

Comments: The four spike proteins were incubated with L-NPs, the supernatant and precipitation were separated by centrifugation (8000 rpm, 10 min), and the CD spectrum of supernatant and precipitation were tested at different time points.

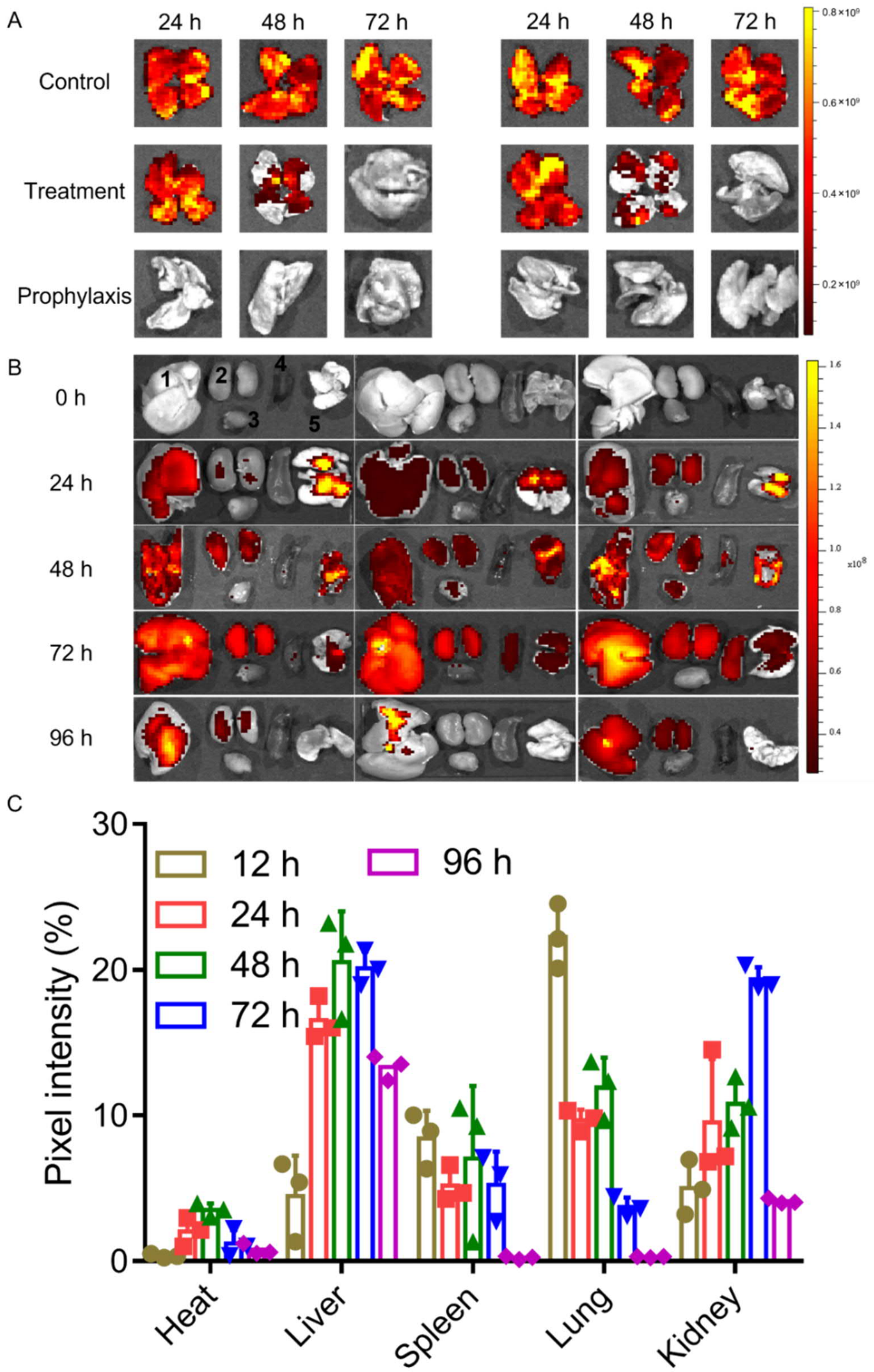

Fig. S41. (A) Raw ex vivo IVIS imaging of lung tissues from mice with various treatments (n=3). **(B)** Ex vivo fluorescent images of lung organs at various time points after inhalation of L-NPs. 1, 2, 3, 4, and 5 correspond to the liver, kidney, heart, spleen and lung, respectively (n = 3). **(C)** Corresponding pixel intensity from B of chiral NPs in heart, lung, liver, kidney, and spleen tissues. Data are presented as mean ± SD (n = 3) with the error bar representing the standard deviation.

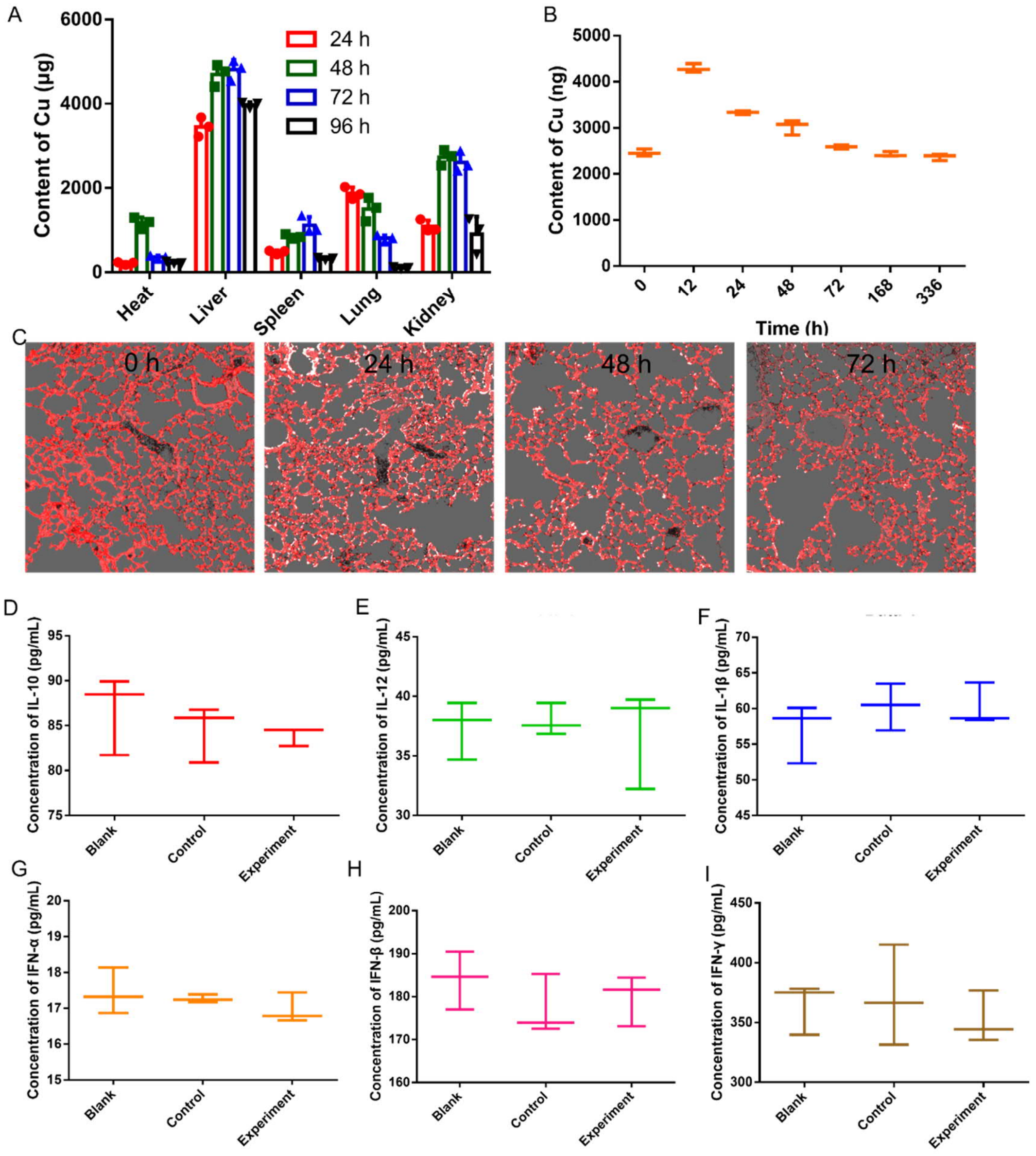

Fig. S42. (A) Results from ICP-MS of chiral NPs in heart, lung, liver, kidney, and spleen tissues. **(B)** Metabolic dynamics after inhalation of L-NPs in blood. **(C)** Representative confocal images of L-NPs (white) in lung tissue section. (D-I) Toxicologic studies on the L-NPs inhalation therapy. Inflammatory cytokine detection. **(D)** IL-10, **(E)** IL-12, **(F)** IL-1β, **(G)** IFN-α, **(H)** IFN-β, and **(I)** IFN-γ levels in the mouse sera were measured by ELISA. The data points represent mean ± SD (n = 3).

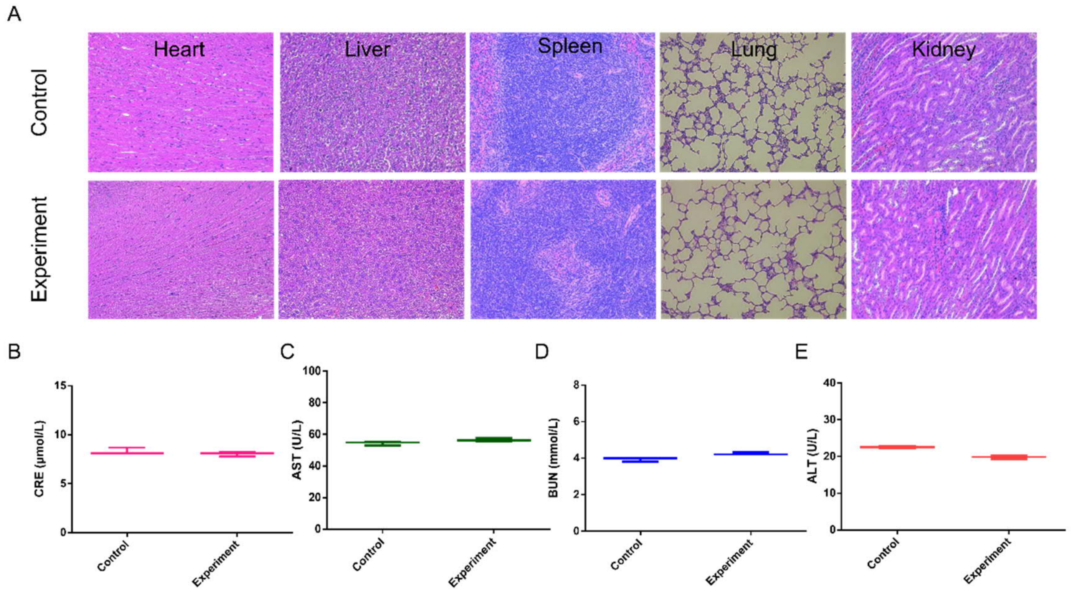

Fig. S43. (A) Toxicologic studies on the chiral NPs inhalation therapy. H&E staining of major organs after chiral NPs treatment (n = 3). (E-H) Blood biochemistry analysis. **(E)** CRE: creatinine, **(F)** AST: aspartate aminotransferase, **(G)** BUN: blood urea nitrogen, **(H)** ALT: alanine transaminase. The data points represent mean ± SD (n = 3).

Tab. S1. The zeta-potential values of NPs and their mixtures with RBD. Data are presented as three independent experimental results (n = 3).

Type of NPs	Zeta potential (mV) (Mean±s.d.)
L-NPs	-8.00 ± 0.23
L-NPs + RBD	-39.90 ±1.20
D-NPs	-9.37 ± 0.31
D-NPs + RBD	-26.87 ± 1.02
rac-NPs	-17.90 ± 0.12
rac-NPs + RBD	-15.50 ± 1.41
Cit-NPs	-60.03 ± 1.04
Cit-NPs + RBD	-56.73 ± 8.11
MA-NPs	-26.87 ± 1.03
MA-NPs + RBD	-22.80 ± 0.32
TA-NPs	-15.50 ± 1.43
TA-NPs + RBD	-11.83 ± 0.14
GSH-NPs	-8.54 ± 0.31
GSH-NPs + RBD	-5.86 ± 0.81
CuS NPs (8 nm)	-11.37 ± 0.92
CuS NPs (8 nm) + RBD	-11.60 ± 2.40
CuS NPs (20 nm)	-23.53 ± 1.42
CuS NPs (20 nm) + RBD	-20.87 ± 0.22
CuS NRs	7.24 ± 1.12
CuS NRs + RBD	3.36 ± 1.51
$Cu_{1.94}S$ NPs	-22.20 ± 0.81
$Cu_{1.94}S$ NPs + RBD	-17.00 ± 1.21
SiO_2 NPs	5.27 ± 0.91
SiO_2 NPs + RBD	5.73 ± 0.34
ZnO NPs	2.52 ± 0.13
ZnO NPs + RBD	1.01 ± 0.05
Au NPs	-3.08 ± 1.21
Au NPs + RBD	-2.98 ± 0.42
RBD	2.60 ± 0.09
S1	-12.34±0.64
S	-59.94 ±1.45

Tab. S2. The IC_{50} values of chiral NPs to three viral proteins in PBS and HEK293 cells.

Groups	IC_{50}/pM (ng/mL)		Affinity Constant (K_a)
	PBS	Cells	
L-NPs to S	0.51 (1.11)	1.09 (2.37)	$(5.31 \pm 0.28) \times 10^8$
L-NPs to S1	1.28 (2.78)	2.87 (6.24)	$(8.66 \pm 0.41) \times 10^7$
L-NPs to RBD	0.66 (1.44)	1.83 (3.98)	$(2.35 \pm 0.12) \times 10^8$
D-NPs to S	5.29 (11.51)	12.04 (26.19)	$(5.22 \pm 0.26) \times 10^7$
D-NPs to S1	14.33 (31.17)	31.26 (68.00)	$(2.02 \pm 0.11) \times 10^7$
D-NPs to RBD	6.91 (15.03)	21.63 (47.05)	$(2.33 \pm 0.12) \times 10^7$
rac-NPs to S	16920 (36810)	17510 (38090)	$(3.16 \pm 0.16) \times 10^4$
rac-NPs to S1	87020 (189310)	95950 (208730)	$(9.16 \pm 0.48) \times 10^5$
rac-NPs to RBD	48270 (105010)	52160 (113470)	$(6.52 \pm 0.29) \times 10^4$

Tab. S3. IC50 values of chiral NPs inhibiting the binding between viral protein and ACE2 under different treatments.

Different groups	IC_{50}/pM (ng/mL)		
	Treatment 1	Treatment 2	Treatment 3
L-NPs to Spike	0.52 (1.13)	0.63 (1.38)	0.81 (1.76)
L-NPs to S1	1.14 (2.48)	1.67 (3.63)	2.06 (4.48)
L-NPs to RBD	0.68 (1.48)	0.79 (1.72)	1.29 (2.81)
D-NPs to Spike	5.72 (12.44)	6.73 (14.64)	8.37 (18.21)
D-NPs to S1	15.92 (34.63)	18.89 (41.09)	25.12 (54.65)
D-NPs to RBD	7.75 (16.86)	8.67 (18.86)	13.51 (29.39)
rac-NPs to Spike	32,290 (70,245)	27,140 (59,041)	22,170 (48,229)
rac-NPs to S1	102,310 (222,569)	97,530 (212170)	91,020 (198,008)
rac-NPs to RBD	47,260 (102,811)	41,330 (89,911)	21,840 (47,512)

Movie S1 (separate file). 3D reconstruction of achiral and chiral tapered NPs from ADF - STEM images. The solid spheres are locations of atomic columns based on image intensities in ADF-STEM images.

Movie S2 (separate file). 3D tomography reconstruction of L-NPs incubated with Spike proteins of SARS-CoV-2 for 2 h.

Movie S3 (separate file). 3D tomography reconstruction of D-NPs incubated with Spike proteins of SARS-CoV-2 for 2 h.

SI References

1. Li W*, et al.* (2011) Morphology evolution of Cu(2-x)S nanoparticles: from spheres to dodecahedrons. *Chem Commun (Camb)* 47(37):10332-10334.
2. Hsu SW, Ngo C, & Tao AR (2014) Tunable and directional plasmonic coupling within semiconductor nanodisk assemblies. *Nano Lett* 14(5):2372-2380.
3. Huang F, Wang X, Xu J, Chen D, & Wang Y (2012) A plasmonic nano-antenna with controllable resonance frequency: Cu1.94S–ZnS dimeric nanoheterostructure synthesized in solution. *J Mater Chem* 22(42): 16326-16334.
4. Fenton JL, Steimle BC, & Schaak RE (2018) Exploiting Crystallographic Regioselectivity To Engineer Asymmetric Three-Component Colloidal Nanoparticle Isomers Using Partial Cation Exchange Reactions. *J Am Chem Soc* 140(22):6771-6775.
5. Chen Y*, et al.* (2014) Hollow mesoporous organosilica nanoparticles: a generic intelligent framework-hybridization approach for biomedicine. *J Am Chem Soc* 136(46):16326-16334.
6. Yang M, Sun K, & Kotov NA (2010) Formation and assembly-disassembly processes of ZnO hexagonal pyramids driven by dipolar and excluded volume interactions. *J Am Chem Soc* 132(6):1860-1872.
7. Liu J*, et al.* (2016) Luminescent Gold Nanoparticles with Size-Independent Emission. *Angew Chem Int Ed Engl* 55(31):8894-8898.
8. Yeom J*, et al.* (2018) Chiromagnetic nanoparticles and gels. *Science* 359(6373):309-314.
9. Ching-Lin Hsieh JAG, Jeffrey M. Schaub, Andrea M. DiVenere, Hung-Che Kuo, Kamyab Javanmardi, Kevin C. Le, Daniel Wrapp, Alison G. Lee, Yutong Liu, Chia-Wei Chou, Patrick O. Byrne, Christy K. Hjorth, Nicole V. Johnson, John Ludes-Meyers, Annalee W. Nguyen, Juyeon Park, Nianshuang Wang, Dzifa Amengor, Jason J. Lavinder, Gregory C. Ippolito, Jennifer A. Maynard, Ilya J. Finkelstein, Jason S. McLellan (2020) Structure-based design of prefusion-stabilized SARS-CoV-2 spikes. *Science* 369(6510):1501-1505.
10. Kirchdoerfer RN*, et al.* (2016) Pre-fusion structure of a human coronavirus spike protein. *Nature* 531(7592):118-121.
11. Wang C*, et al.* (2022) Antigenic structure of the human coronavirus OC43 spike reveals exposed and occluded neutralizing epitopes. *Nat Commun* 13(1):2921.
12. Walls AC*, et al.* (2016) Glycan shield and epitope masking of a coronavirus spike protein observed by cryo-electron microscopy. *Nat Struct Mol Biol* 23(10):899-905.
13. He C*, et al.* (2022) Spike protein of SARS-CoV-2 Omicron (B.1.1.529) variant have a reduced ability to induce the immune response. *Signal Transduct Target Ther* 7(1):119.
14. Cubuk J*, et al.* (2021) The SARS-CoV-2 nucleocapsid protein is dynamic, disordered, and phase separates with RNA. *Nat Commun* 12(1):1936.
15. Yang J*, et al.* (2020) Molecular interaction and inhibition of SARS-CoV-2 binding to the ACE2 receptor. *Nat Commun* 11(1):4541.
16. Whitman JD*, et al.* (2020) Evaluation of SARS-CoV-2 serology assays reveals a range of test performance. *Nat Biotechnol* 38(10):1174-1183.
17. De Backer A, van den Bos KHW, Van den Broek W, Sijbers J, & Van Aert S (2016) StatSTEM: An efficient approach for accurate and precise model-based quantification of atomic resolution electron microscopy images. *Ultramicroscopy* 171(1):104-116.
18. Van Aert S, Batenburg KJ, Rossell MD, Erni R, & Van Tendeloo G (2011) Three-dimensional atomic imaging of crystalline nanoparticles. *Nature* 470(7334):374-377.
19. Van Aert S*, et al.* (2013) Procedure to count atoms with trustworthy single-atom sensitivity. *Physical Review B* 87(6):064107.
20. De Backer A, Martinez GT, Rosenauer A, & Van Aert SJU (2013) Atom counting in HAADF STEM using a statistical model-based approach: methodology, possibilities, and inherent limitations. 134(1):23-33.
21. J. M. COWLEY AFM (The scattering of electrons by atoms and crystals. I. A New Theoretical Approach. *Acta Cryst* 10(609):609-619.
22. Momma K & Izumi F (2011) VESTA 3for three-dimensional visualization of crystal, volumetric and morphology data. *Journal of Applied Crystallography* 44(6):1272-1276.
23. Evans HTJ & Konnert JAJAM (1976) Crystal structure refinement of covellite. *American Mineralogist* 61(1):996-1000.

24. Grimme S, Bannwarth C, & Shushkov P (2017) A Robust and Accurate Tight-Binding Quantum Chemical Method for Structures, Vibrational Frequencies, and Noncovalent Interactions of Large Molecular Systems Parametrized for All spd-Block Elements (Z = 1-86). *J Chem Theory Comput* 13(5):1989-2009.
25. Gao R, *et al.* (2022) Site-selective proteolytic cleavage of plant viruses by photoactive chiral nanoparticles. *Nature Catalysis* 5(8):694-707.
26. Huang J & MacKerell AD, Jr. (2013) CHARMM36 all-atom additive protein force field: validation based on comparison to NMR data. *J Comput Chem* 34(25):2135-2145.
27. Fuchs JF, *et al.* (2006) New model potentials for sulfur-copper**(I)** and sulfur-mercury**(II)** interactions in proteins: from ab initio to molecular dynamics. *J Comput Chem* 27(7):837-856.
28. Zoete V, Cuendet MA, Grosdidier A, & Michielin O (2011) SwissParam: a fast force field generation tool for small organic molecules. *J Comput Chem* 32(11):2359-2368.
29. Lan J, *et al.* (2020) Structure of the SARS-CoV-2 spike receptor-binding domain bound to the ACE2 receptor. *Nature* 581(7807):215-220.
30. Berendsen HJC, Postma JPM, van Gunsteren WF, DiNola A, & Haak JR (1984) Molecular dynamics with coupling to an external bath. *The Journal of Chemical Physics* 81(8):3684-3690.
31. Nosé S (1984) A unified formulation of the constant temperature molecular dynamics methods. *Journal of Chemical Physics* 81:511-519.
32. Hoover WG (1985) Canonical dynamics: Equilibrium phase-space distributions. *Phys Rev A Gen Phys* 31(3):1695-1697.
33. Parrinello M & Rahman A (1981) Polymorphic transitions in single crystals: A new molecular dynamics method. *Journal of Applied Physics* 52(12):7182-7190.
34. Darden T, York D, & Pedersen L (1993) Particle mesh Ewald: AnN·log**(N)** method for Ewald sums in large systems. *The Journal of Chemical Physics* 98(12):10089-10092.
35. Essmann U, *et al.* (1995) A smooth particle mesh Ewald method. *The Journal of Chemical Physics* 103(19):8577-8593.
36. Hess B, Bekker H, Berendsen HJC, & Fraaije JGEM (1997) LINCS: A linear constraint solver for molecular simulations. *Journal of Computational Chemistry* 18(12):1463-1472.
37. Abraham MJ, *et al.* (2015) GROMACS: High performance molecular simulations through multi-level parallelism from laptops to supercomputers. *SoftwareX* 1-2:19-25.
38. Páll S, Abraham MJ, Kutzner C, Hess B, & Lindahl E (2015) Tackling Exascale Software Challenges in Molecular Dynamics Simulations with GROMACS. *Solving Software Challenges for Exascale*, eds Markidis S & Laure E (Springer International Publishing), pp 3-27.
39. Van Der Spoel D, *et al.* (2005) GROMACS: fast, flexible, and free. *J Comput Chem* 26(16):1701-1718.
40. Cha M, *et al.* (2022) Unifying structural descriptors for biological and bioinspired nanoscale complexes. *Nature Computational Science* 2(4):243-252.
41. Mistek-Morabito E & Lednev IK (2020) Discrimination between human and animal blood by attenuated total reflection Fourier transform-infrared spectroscopy. *Communications Chemistry* 3(1):178.
